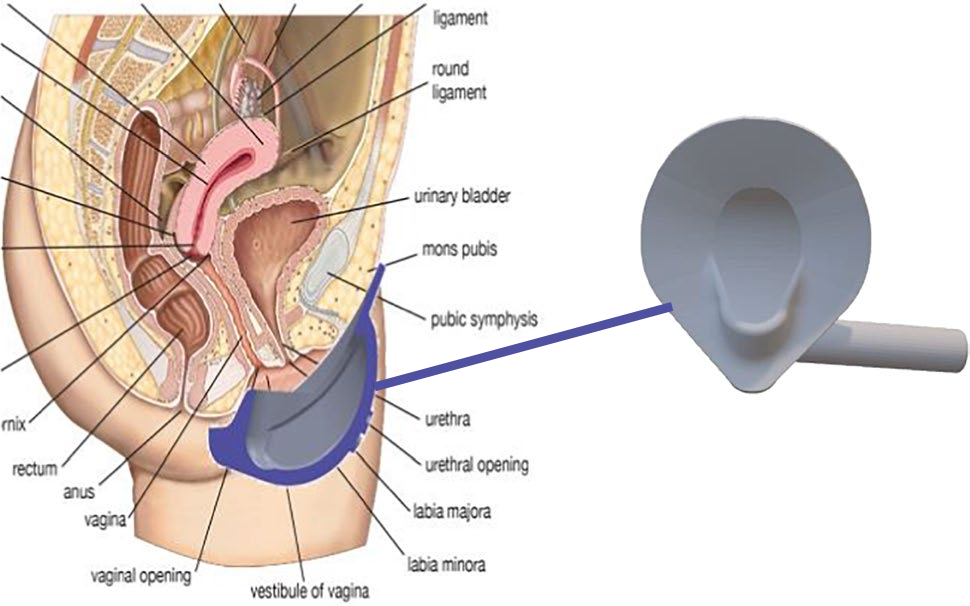# 4th International Symposium on Acute Kidney Injury in Children

**DOI:** 10.1007/s00467-022-05843-4

**Published:** 2023-01-30


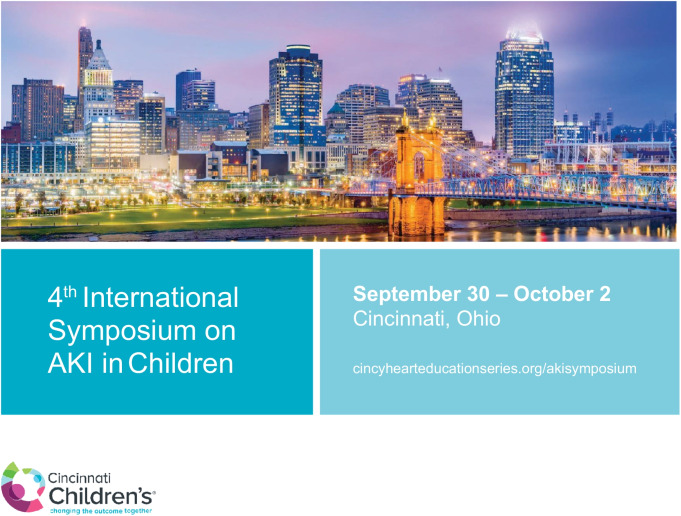
**Agenda**

**FRIDAY, SEPTEMBER 30**

7:00–7:15pm Welcome and Introductions | *S. Goldstein*

7:15–8:00 pm Dialy-Trauma in the ICU | *R. Bellomo*

**SATURDAY, OCTOBER 1**

7:45–8:00 am Welcome–Overview | *S. Goldstein*

**8:00–10:15 am AKI – The Current State of Practice**

8:00–8:20 am Lessons from an AKI Survivor | *L. Meigs*

8:20–8:40 am The Pediatric ADQI – Overview | *AA. Arikan*

8:40–9:00 am pADQI Group 1 - AKI Epidmeiology | *S. Gorga*

9:00–9:20 am AKI, AKD and CKD – The Canary Died in the Coal Mine, What the Heck Should I do Now? | *M. Zappitelli*

9:20–9:40 am What is New in Adult AKI Studies | *S. Bagshaw*

9:40–10:00 am Panel

10:00–10:30 am Break

**10:30 am–12:30 pm AKI IN THE PICU**

10:30–10:50 am pADQI Work Group 2 - Risk Stratification and Diagnostics | *D. Fuhrman*

10:50–11:10 am pADQI Work Group 3 - Fluid Management *M. Barhight*

11:10–11:30 am Pathophysiology of Sepsis Associated AKI | *R. Basu*

11:30–11:50 am Sepsis and Sepsis Associated AKI Prediction Models | *N. Stanski*

11:50 am–12:10 pm pADQI Work Group 4 - Renal Support *T. Neumayr*

12:10–12:30 pm Panel

1:00–2:00 pm Lunch

**2:00–2:20 pm Top Abstracts – Oral Presentations**

2:00–2:10 pm Top Abstract #1

2:10–2:20 pm Top Abstract #2

**2:20–3:30 pm AKI in the NICU**

2:20–2:40 pm The Current State of the Art | *J. Jetton*

2:40–3:00 pm Neonatal AKI RRT Technology | *C. Slagle*

3:00–3:20 pm From Burrito to Toddler – Can Injured Neonatal Beans Be REPaIReD? *D. Askenazi*

3:20–3:30 pm Panel

3:30–4:00 pm Break

**4:00–5:10 pm AKI in the CICU**

4:00–4:20 pm The Whole Enchilada - Risk, Prevention, Diagnosis, Support and Follow Up *D. Cooper*

4:20–4:40 pm Pre-operative and Post-Operative Biomarkers to Predict Post-Operative AKI *K. Gist*

4:40–5:00 pm Mechanical Support: LVADs and ECMO and AKI | *Z. Ricci*

5:00–5:10 pm Panel

**5:10–5:30 pm The Other Metabolic Organ - AKI in Liver Failure**

5:10–5:30 pm CRRT and Anticoagulation | *A. Deep*

5:30–5:50 pm Liver Dialysis and TPE | *AA. Arikan*

5:50–6:00 pm Panel

**SUNDAY, OCTOBER 2**

7:45–8:00 am Welcome Overview | *A. Deep*

**8:00–9:45 am Innovation in Pediatric AKI and CRRT Therapies**

8:00–8:30 am pADQI Group 5 - Pathobiology, Nutrition and Pharmacology of pAKI | *D. Soranno*

8:30–8:50 am Cutting Edge AKI Outside the Ivory Tower | *A. Conroy*

8:50–9:10 am AKI Therapeutics | *P. Devarajan*

9:10–9:30 am Novel Supoprtive Therapies--It’s not your Grandmother’s CRRT | *S. Goldstein*

9:30–9:45 am Panel

9:45–10:15 am Break

**10:15 am–1:00 pm AKI – Where We Need to Be**

10:15–10:40 am From Bench to Bedside - Do we Know what AKI really is? *D. Soranno*

10:35–11:00 am pADQI Group 6 - AKI Education and Advocacy | *K. Dolan*

11:00–11:20 am What is Needed for AKI/CRRT QA/QI *T. Mottes*

11:20–11:50 am What Are the Important AKI Studies That Need to Be Done? | *S. Bagshaw*

11:50 am–12:20 pm pADQI - Research Agenda | *R. Basu*

12:20–12:40 pm Putting it All Together | *S. Goldstein*

12:40–12:55 pm Panel

12:55–1:00 pm Closing Remarks *A. Deep and S. Goldstein*

**Abstract 01 – Oral Presentation**

**Assessment of Renin-Angiotensin-Aldosterone System Derangement in Pediatric Septic Shock**

Naomi Pode Shakked^1,2^, Giovanni Ceschia^3^, James Rose^1^, Stuart L. Goldstein, MD^1^, Natalja L. Stanski^1^

^*1*^ *Cincinnati Children’s Hospital Medical Center;* ^*2*^*Sackler School of Medicine, Tel Aviv University, Israel;* ^*3*^*University of Pauda, Italy*

**Background:** Elevated serum renin concentrations are associated with poor outcomes in critically ill adults. Data suggest that these associations may be stronger in children with septic shock, in whom endothelial damage is hypothesized to lead to angiotensin-converting enzyme (ACE) dysfunction and derangements in the renin-angiotensin-aldosterone system (RAAS) (**Figure 1**). However, no studies support this hypothesis. We examined the relationship between renin levels, ACE levels, ACE activity and outcomes in children with septic shock.

**Methods:** A pilot study using subjects from a multicenter observational study of pediatric septic shock. 72/379 children were selected based on the availability of residual Day 1 (D1) serum. On D1, serum renin concentrations were measured by Luminex® assay (normal <59 pg/ml), serum ACE levels via ELISA assay (median in healthy children: 200 ng/ml), and ACE activity by high-sensitivity enzymatic assay (normal adult range: 16-85 U/L; expected 20-50% higher in children). The associations between each RAAS component and outcomes, and their relationship to each other, were assessed.

**Results:** Median D1 renin concentration was 3940 pg/ml (IQR 1749-11671); D1 renin >median was associated with higher D1 vasoactive-inotrope score (VIS), increased odds for severe persistent AKI and 28-day mortality, and fewer PICU-free and vasoactive-free days (**Table 1**). Median D1 ACE level was 104 ng/ml (IQR 78-149), and patients with D1 renin levels >median had *higher* ACE levels (**Table 1**). D1 ACE levels >median were associated with increased odds of severe persistent AKI and renal replacement therapy (RRT) requirement (**Table 1**). 68/72 children had ACE activity <12.050 U/L, with 50/72 (69%) measuring undetectable (<2.41 U/L). Compared to those with detectable ACE activity, ACE activity <2.41 U/L was associated with higher D1 renin concentrations (median 4533 [IQR 2782-21834] *vs*. 2227 [IQR 1053,6284], *p*=0.017); ACE levels did not differ between these 2 groups.

**Conclusions:** Elevated serum renin concentrations are associated with poor outcomes in pediatric septic shock, and ACE levels and activity measure below normal ranges in these children. Though elevated renin concentrations are associated with *lower* ACE activity, they appear to be associated with paradoxically *higher* ACE levels. Further study is needed to examine mechanisms of RAAS dysfunction in these children. 
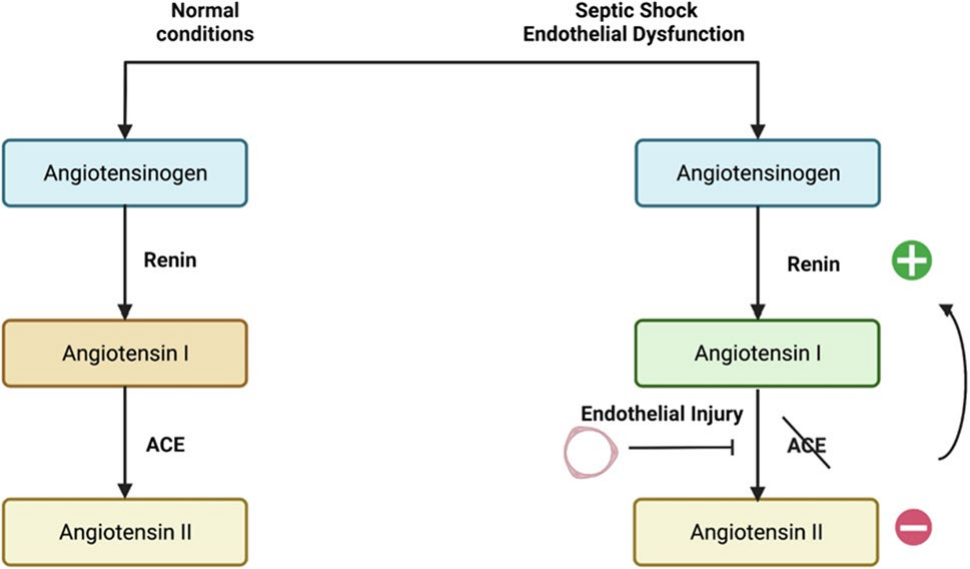


**Figure 1: Hypothesized Mechanism of RAAS Derangement in Septic Shock**

**Table 1: Patient demographics, clinical characteristics and outcomes stratified by Day 1 renin concentration and ACE levels above and below the cohort median.**

**Table:** 

	**All Patients**	**Renin**		**Comparison**	**ACE Level**		**Comparison**
		**<3940 pg/ml**	**>3940 pg/ml**		**<104 ng/ml**	**>104 ng/ml**	
N (% cohort)	72	36 (50)	36 (50)	--	36 (50)	36 (50)	--
Age, years	12.1 (3.2,17.7)	14.3 (7.4,18)	7.2 (2.1,17)	0.15	12.7 (3.6,18)	11 (2.3,17)	0.67
Sex, n (% male)	27 (38)	14 (39)	13 (36)	0.81	16 (44)	11 (31)	0.22
PRISM III	11.5 (8.3,15)	10.6 (7.3, 15)	12 (9.3,16)	0.50	10.7 (8.3,15)	12 (8.3,15.8)	0.86
D1 Vasoactive, n (%)	63 (88)	30 (83)	33 (92)	0.48	32 (89)	31 (86)	1.0
D1 VIS	12.5 (5,30)	9.5 (2.5,25)	18.5 (10,40)	0.028	10 (5,25)	18.6 (8.3,45)	0.11
D1 Renin, pg/ml	3940 (1749,11671)	--	--	--	3288 (1267,7557)	6096 (2539,19903)	0.092
D1 ACE, ng/ml	104 (78, 149)	94 (79,118)	133 (75,164)	0.07	--	--	--
D1-7 Severe Persistent AKI, n (%)	18 (25)	4 (11)	14 (39)	OR 5.1 (95%CI 1.5-17.5, *p*=0.006)	3 (8)	15 (42)	OR 7.9 (95%CI 2.0-30.5, *p*=0.001)
D1-7 RRT, n (%)	12 (17)	3 (8)	9 (25)	OR 3.7 (95%CI 0.9-14.9, *p*=0.058)	2 (6)	10 (28)	OR 6.5 (95%CI 1.3-32.4, *p*=0.011)
PICU-Free Days (28-day)	13.5 (0,24)	20 (9.8,25)	0 (0, 24)	0.005	17.5 (0,24)	12 (0,24)	0.36
Vasoactive-Free Days (7-day)	4 (2,5)	5 (3,5)	4 (0.25,5)	0.05	4 (3.25,5)	3.5 (1,5)	0.17
Mortality, n (%)	19 (26)	5 (14)	14 (39)	OR 3.9 (95%CI 1.2-12.6, *p*=0.016)	7 (19)	12 (33)	OR 2.1 (95%CI 0.71-6.1, *p*=0.18)

Continuous variables reported as median (IQR)

**Abstract 02 - Oral Presentation**

**Food Insecurity Is Associated with an Increased Risk of Neonatal Acute Kidney Injury and Abnormal Kidney Function at 2 weeks of age**

Michelle C. Starr^1*,2^, Abigail C. Delbridge^1^, Samantha W. Wallace^1^, Paulomi Chaudhry^1^, Danielle E. Soranno^1^

^*1*^ *Indiana University School of Medicine, Indianapolis, IN, USA;* ^*2*^*Center for Pediatric and Adolescent Comparative Effectiveness Research, Indiana University, Indianapolis, IN, USA*

**Background:** Food insecurity (FI) is an important social determinant of health which negatively affects both maternal and infant health. Maternal malnutrition, often due to FI, leads to decreased kidney mass in neonate and FI accelerates kidney disease progression. Therefore**,** the effects of FI on kidney outcomes may be more notable in neonates. We also have a pilot animal model demonstrating that maternal malnutrition leads to worse neonatal kidney outcomes after AKI. The aim of this clinical study was to determine the association between FI and kidney outcomes in neonates.

**Methods:** We conducted a single-site prospective cohort study. Neonates admitted to the NICU at Riley Children’s Health, Indianapolis, IN were enrolled. We collected demographic characteristics and FI status using the Hunger Vital Signs. Kidney outcomes (early AKI within first 14 days, recovery from AKI [creatinine within 0.2mg/dL of baseline within 7 days], and creatinine at day 14 of age) were compared by FI status.

**Results:** Of the 45 neonates in this study, 22% (10/45) lived in FI households. Infants from FI households were more likely to have early AKI (60% vs 17%, *p=*0.007) and were more likely to have slower recovery from AKI (40% vs 17%, *p=*0.035) than those from food secure households. Neonates from FI households were also more likely to have an abnormal serum creatinine at two weeks of age (70% vs 31%, *p=*0.028). While not statistically significant, neonates from FI households had a trend towards higher likelihood of IUGR (30% vs 14%).

**Conclusion:** Food insecurity is common in families with neonates admitted to the NICU. Neonates born into FI households were more likely to have early AKI, slower AKI recovery, and abnormal kidney function at 2 weeks of age. Future work includes further expansion of this prospective cohort and focus on long-term kidney outcomes. Our preclinical model of FI will enable translational investigations of the impact of FI on long-term kidney functional and histological outcomes. Our findings emphasize the importance of FI screening and recognition as part of prenatal care and as an essential social determinant of health in studies investigating kidney outcomes.

**Keywords:** Neonatal kidney disease, food insecurity, social determinants of health, disparities.

**Disclosures:** The authors declared no competing interests.

**Corresponding Author:** Michelle C. Starr, https://orcid.org/0000-0001-9412-8950

**References:** None

**Table 1.** Maternal and infant characteristics and kidney health outcomes by food insecurity status. Data presented as number (percentage) or as mean (SD)

**Table:** 

	Cohort (N=45)	Food Secure (N=35) (77.8%)	Food Insecure (N=10) (22.2%)	p-value
**Maternal Characteristics**				
Maternal age (years), mean (SD)	28 (5)	29 (4)	26 (4)	.21
White, n (%)	38 (84)	30 (86)	8 (80)	.66
Non-Hispanic, n (%)	42 (93)	32 (91)	10 (100)	.99
Highest educational level completed				.005
High school graduate/GED, n (%)	24 (53)	14 (40)	10 (100)	
Associates degree or higher, n (%)	21 (47)	21 (60)	0 (0)	
Average Household Income				.08
Greater than $50,000 per year, n (%)	26 (60)	25 (76)	1 (10)	
Multiple gestations, n (%)	9 (20)	8 (23)	1 (10)	.37
Oligohydramnios, n (%)	1 (2)	0 (0)	1 (10)	.89
Polyhydramnios, n (%)	6 (13)	3 (9)	3 (30)	.079
IUGR, n (%)	8 (18)	5 (14)	3 (30)	.12
**Infant Characteristics**				
Male, n (%)	25 (56)	20 (57)	5 (50)	.86
Gestational age (weeks), mean (SD)	32 (5)	32 (5)	32 (4)	.84
Gestational age, n (%)				.45
22 – 27 6/7 weeks	12 (27)	11 (31)	1 (10)	
28 – 36 weeks	20 (44)	14 (40)	6 (60)	
>37 weeks	13 (29)	10 (29)	3 (30)	
Birthweight (grams), mean (SD)	1894 (921)	1892 (982)	1902 (768)	.98
**Kidney Health**				
Early Acute Kidney Injury, n (%)	12 (27)	6 (17)	6 (60)	.007
Abnormal kidney function at 14 days, n (%)^1^	20 (44)	11 (31)	7 (70)	.028
Slow recovery from AKI, n (%)	10 (22)	6 (17)	4 (40)	.035
Nephrology consultation, n (%)	10 (22)	7 (20)	3 (30)	.50

^1.^Determined by serum creatinine >0.5mg/dL at 2 weeks of age

^2.^Determined by serum creatinine >0.2mg/dL from baseline 7 days after AKI episode

**Abstract 03**

**Multi-Center Study to Assess the Safety of a Selective Cytopheretic Device (SCD) for treatment of immunomodulatory dysregulation due to AKI in children ≥10 and ≤ 20kg: Report from the First 6 Patients**

Angela J. Westover^2*^, Chris Pino^2^, H. David Humes^2^*, Kelli A. Krallman^1^, David J. Askenazi^3^, Lenar T. Yessayan^2^, Michaela Collins^1^, Jessica Potts^3^, Stuart L. Goldstein^1^

^*1*^ *Cincinnati Children's Hospital Medical Center, Cincinnati, OH, United States;* ^*2*^*University of Michigan Medical School, Ann Arbor, MI, United States;* ^*3*^*Benjamin Russell Hospital for Children, Birmingham, AL, United States*

**Background:** Acute kidney injury (AKI) that requires continuous kidney replacement therapy (CKRT) is a highly lethal condition in critically ill patients. Despite improvements in acute care and dialysis therapies, the mortality rate from the past four decades has not improved. In a previous single treatment arm study of children >20kg on CKRT treated with SCD, 12/16 subjects survived to ICU discharge, all of whom were dialysis independent by day 60 [1]. We now report outcomes in findings the first 6 treated patients < 20 kg.

**Methods:** 6 center US study of SCD in children that weigh between ≥10 and ≤ 20kg, have a clinical diagnosis of AKI requiring CKRT and at least one non-renal organ failure. With these subjects the SCD was integrated post CKRT hemofilter, changed daily, and circuit ionized calcium (iCa) maintained <0.4 mmol/L. Subjects received SCD treatment for up to 10 days or CKRT discontinuation, whichever came first.

**Results:** 7 patients (2F/5M) have been enrolled since 07/2021. Six patients (Age 1.7 - 10.4 years. PRISM-2 Score range = 12-27) received SCD therapy. Patients received SCD on a PRISMAFLEX™ or on a PRISMAX™ machine. Circuit iCa was targeted at <0.4 mmol/L during 90% of therapy. All surviving patients demonstrated renal recovery after SCD therapy, coming off all forms of KRT within 5 days post SCD therapy. Of the patients that have reached day 60, all were dialysis independent. One patient died after SCD therapy had ended but before ICU discharge. An additional patient was enrolled but did not start SCD treatment because iCa was not in range from citrate intolerance. No SCD-related serious adverse events have been reported.

**Conclusion:** Our initial findings suggest that SCD is safe in critically ill pediatric patients weighing between 10-20kg and appears to have probable benefit.

**Disclosures:** Study was funded by the Frankel Innovation Initiative, University of Michigan. Devices supplied by SeaStar Medical, Inc. HDH, CJP, AJW have financial relationships to SeaStar Medical.

**Clinical Trial Registry:** NCT04869787; https://clinicaltrials.gov

**Corresponding Author:** H. David Humes, MD. dhumes@umich.edu

**References:**

1. Goldstein, SL, et.al. Kidney Int Rep (2021). https://doi.org/10.1016/j.ekir.2020.12.010

2. Goldstein, SL, et. al. Pediatric Nephrology (2022). https://doi.org/10.1007/s00467-022-05692-1

**TABLE**: Patient Information Summary

**Table:** 

Patient Number	Age (yrs)	Weight (kg)	Diagnosis	Final Outcome
1	1.9	14.1	Shock and pancytopenia from HLH [2]	Alive with renal recovery
2	1.7	12	Necrotizing pneumonia/ECMO	Deceased
3	10.4	16.5	Status epilepticus	Alive with renal recovery
4	3.5	14.3	Bowel perforation and septic shock	Alive with renal recovery
5	2.0	13.9	Hemolytic Uremic Syndrome/STEC	Alive with renal recovery
6	2.0	12.9	Hemolytic Uremic Syndrome/STEC	Alive with renal recovery

**Abstract 04**

**Urine NGAL and the Impact on Therapeutic Dose Monitoring (TDM) of Aminoglycosides and Vancomycin in a Neonatal Intensive Care Unit (NICU)**

Trina Hemmelgarn^1*^, Dawn Butler^1^, Bri Hemmann^1^, Cara Slagle^1^

^*1*^ *Cincinnati Children’s Hospital, Cincinnati, Ohio*

**Background:** Nephrotoxic medication exposure is associated with neonatal Acute Kidney Injury (AKI). Nephrotoxic Injury Negated by Just-in-time Action in neonates (Baby NINJA) is an automated screening process aimed to reduce nephrotoxic medication (NTM) exposure, AKI prevalence and intensity. Rather than daily serum draws for creatinine (sCr) screening, urine neutrophil gelatinase-associated lipocalin (uNGAL) is a less invasive alternative screening tool for neonatal AKI. The impact of elevated uNGAL values on therapeutic drug monitoring (TDM) is currently unknown.

**Methods:** This is a single center observational study following the introduction of uNGAL into the Baby NINJA screening algorithm in June of 2021. Urine NGAL is used unless serum labs are being obtained for other indications. The uNGAL is reviewed daily and if >150 ng/dL, then screening is transitioned to daily sCr monitoring until 2 days following the end of NTM exposure or 7 days. Infants less than 12 months of age who were admitted to the CCHMC NICU with uNGAL concentrations and on aminoglycosides and/or vancomycin from 6/15/2021 – 7/31/2022 were included in this study. The primary aims were to describe the current practice for TDM in patient with uNGAL concentrations >150 and to determine if there is a correlation between these values and incidence of supratherapeutic drug levels.

**Results:** A total of 15 episodes in 13 subjects met inclusion criteria. The median birth weight was 0.7 kg (IQR: 0.55, 1.365 kg) and median gestational age was 30 weeks (IQR: 23, 31 weeks). Elevated uNGAL values ranged from 175 to 8778 ng/mL. Three of 15 (20%) TDM levels were obtained prior to standard practice secondary to elevated uNGAL levels (221, 2088, and 8778 ng/mL). Out of the 15 TDM levels, seven levels were within goal range, two were supratherapeutic, and six were subtherapeutic. None of the TDM levels drawn early were supratherapeutic.

**Conclusions:** The results of this pilot study in a small patient population did not show a correlation between elevated uNGAL values and increased TDM. Although elevated uNGAL values did not predict supratherapeutic drug levels, further studies are warranted.

**Key Words: **Baby NINJA, Urine NGAL, Biomarkers, Neonatal AKI

**Disclosures: **None

**References: **None

**Abstract 05**

***In Vitro*** **Correlation of Cefepime and Meropenem Concentrations with a Novel Fluorescent Marker of GFR**

H. Rhodes Hambrick^1^, Jeng-Jong Shieh^2^, Richard B. Dorshow^2^, and Stuart L. Goldstein^1^

^*1*^ *Cincinnati Children’s Hospital Medical Center, Cincinnati, OH, USA;* ^*2*^*MediBeacon Inc., St. Louis, MO, USA*

**Background:** There is insufficient data to inform antimicrobial dosing for patients requiring continuous kidney replacement therapy (CKRT), a critically ill population for whom . MB-102 is a novel fluorescent marker of glomerular filtration rate (GFR) whose transdermally-detectable fluorescence decay is an accurate measurement of GFR in patients with GFR as low as 15 mL/min/1.73 m^2^. MB-102 (relmapirazin) concentrations and fluorescence have previously been shown to correlate with effluent meropenem concentrations in an *in vivo* model of CKRT using nephrectomized pigs, but the correlation of MB-102 concentrations with drugs with higher degrees of protein binding, such as cefepime, is unknown.

**Methods:** A mock continuous veno-venous hemofiltration (CVVH) circuit was constructed using a polyethersulfone filter with selectivity for molecules up to 12 kDa. A reservoir solution was prepared containing equimolar concentrations of MB-102 with meropenem or cefepime in a mixture of human plasma and phosphate-buffered saline. The ultrafiltrate was collected at intervals from 30 to 360 minutes while the retentate was re-circulated to the reservoir for refiltration. Concentrations of MB-102, meropenem, and cefepime in the ultrafiltrate were assessed by high performance liquid chromatography.

**Results:** MB-102, cefepime, and meropenem were all detectable in the ultrafiltrate throughout the experiment. The ratio of meropenem to MB-102 remained relatively constant throughout (range 0.97-1.20, mean 1.17, SD 0.097; r^2^ for linear correlation 0.99). The ratio of cefepime to MB-102 was initially low at 0.20 from 0-30 mins, then rose to 1.51 from 120-180 minutes before falling to 0.92 from 300-360 minutes.

**Conclusions:** A mock CVVH circuit was successfully designed for use in studying ultrafiltrate concentrations of antibiotics and MB-102. Meropenem, with negligible protein binding, was filtered at a similar rate to MB-102. Cefepime, which has 20% protein binding, was initially filtered less than, then more than, then at the same rate as MB-102, potentially due to initial variability in the local concentration of free cefepime followed by establishment of an equilibrium between cefepime filtration and protein unbinding. Future studies should compare MB-102 filtration to that of drugs with more protein binding to explore the utility of MB-102 in predicting drug concentrations for patients on CKRT.

**Keywords:** CKRT, pharmacokinetics, GFR, MB-102, fluorescent tracer agent

**Corresponding Author:** H. Rhodes Hambrick, MD 
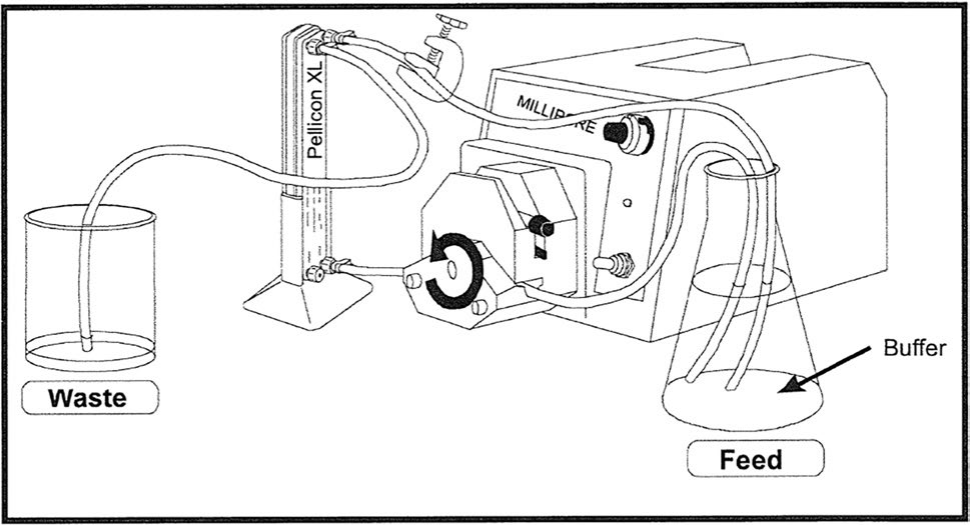


**Figure 1**. Schematic of the mock CVVH system, from the Pellicon XL operating manual. The “feed” solution was the reservoir containing MB-102, plasma, phosphate-buffered saline, and human plasma. This solution was pumped to the filter; the filtrate (here labeled “waste”) was collected, and its contents were analyzed serially as described, while the retentate was returned to the reservoir for recirculation.

**Abstract 06**

**Postnatal Steroid Exposure in Very Low Birthweight Neonates and Associations with Acute Kidney Injury**

Cassandra Coleman^1*^, MD, Jeffrey King^1^, MD, David T. Selewski^1^, MD, MSCR, Jill C. Newman^1^, MS, Heidi J. Steflik^1^, MD, MSCR

^*1*^ *Medical University of South Carolina, Charleston, SC, USA*

**Background:** In a small study investigating mineralocorticoid-responsive circulatory collapse and neonatal acute kidney injury (AKI), it was found that postnatal steroids (PNS) may impact AKI due to relative mineralocorticoid insufficiency [1]. We aimed to investigate associations between PNS exposure and AKI in a large cohort of very low birthweight (VLBW, BW <1500 grams) neonates and hypothesized VLBW neonates exposed to PNS will have fewer episodes of AKI than those without this exposure.

**Methods:** We conducted a single-center chart review of VLBW infants admitted to the neonatal intensive care unit within 48 hours of life between 1/1/2018-12/31/2020. The exposure was PNS receipt (prior to initial AKI if AKI occurred). AKI was diagnosed using the neonatal modified KDIGO creatinine criteria. Comparisons were made between those with and without PNS exposure and those with and without AKI using Chi-squared, Student’s t, and Wilcoxon Rank Sum tests as appropriate. Associations between PNS exposure and AKI were evaluated using generalized linear mixed modeling adjusted for potential confounders (see Table). Outcomes were expressed as adjusted relative risks (RR) with 95% CI’s.

**Results:** Of 567 neonates included, 97 (17.1%) were exposed to PNS, and 130 (22.9%) experienced AKI. Infants with PNS exposure had lower gestational age (GA), birth weight (BW), Apgar scores, were less frequently small for GA (SGA), and experienced more adrenal insufficiency (AI) compared to those without PNS exposure (all p<0.05). Similarly, infants with AKI had lower GA, BW, Apgar scores, were less frequently SGA, and experienced more AI compared to those without AKI (all p<0.05).

PNS exposure was associated with AKI (PNS: 37.1% AKI, no PNS: 20% AKI; p<0.001) as were all analyzed potential confounders. After controlling for GA, BW, Apgar scores, AI, and confounders, PNS exposure remained a significant predictor of AKI (RR 1.72, 95% CI 1.09 – 2.72) (Table).

**Conclusions:** AKI is common in VLBW infants in our cohort. In the current study we show that the need for PNS was associated with increased risk of AKI in VLBW neonates, after controlling for multiple confounders. Further analysis is needed to investigate this association and the role of the adrenal axis in neonatal AKI.

**Keywords:** steroid, acute kidney injury, glucocorticoid, mineralocorticoid

**Disclosures** HJS employer received a grant from Baxter. The authors have no conflicts of interest to disclose.

**Corresponding author:** Cassandra J. Coleman, https://orcid.org/0000-0002-2790-8057

**Reference:** Baserga M WK, Bergstrom B, Chan B. Late-Onset Glucocorticoid-Responsive Circulatory Collapse (LCC). Pediatric Academic Society; Baltimore, Abstract MD2019.

**Table.** Adjusted Relative Risk (RR) and Associated 95% Confidence Intervals (CI) for the Association between Post-natal Steroid Receipt and Acute Kidney Injury

**Table:** 

**Characteristic**		**Adjusted Relative Risk (RR)**	**95% Confidence Interval**	**p-value**
Post-natal Steroid Receipt		**1.72**	**(1.09, 2.72)**	**0.019**
Gestational Age (weeks)		0.92	(0.82, 1.05)	0.245
Birth weight (grams)		0.99	(0.99, 1.00)	0.521
Apgar	1 minute	0.98	(0.89, 1.08)	0.745
	5 minutes	1.02	(0.87, 1.11)	0.784
Adrenal Insufficiency		1.08	(0.66 1.76)	0.771
Caffeine		1.15	(0.40, 3.30)	0.799
Hypotension		1.61	(1.12, 2.32)	**0.011**
Patent ductus arteriosus		1.78	(1.19, 2.65)	**0.005**
Sepsis		1.48	(1.04, 2.11)	**0.030**
Mechanical Ventilation		3.95	(1.82, 8.58)	**<.001**
Necrotizing Enterocolitis		1.30	(0.78, 2.15)	0.316
Nephrotoxic Medication Exposure		1.74	(0.75, 4.07)	0.198

**Abstract 07**

**Urine Complement Factor Ba is an early biomarker for Acute Kidney Injury after pediatric cardiac surgery.**

Erin K. Stenson^1*^ MD, Zhiying You^2^ MD, PhD, Charles L. Edelstein^2^ MD, Shinobu Miyazaki-Anzai^2^, Joshua M. Thurman^2^ MD, Bradley P. Dixon^3^ MD, Prasad Devarajan^4^ MD, Jessica Kendrick^2^ MD, MPH

^*1*^ *Section of Pediatric Critical Care Medicine, University of Colorado School of Medicine, Aurora, CO;* ^*2*^*Division of Renal Disease and Hypertension, University of Colorado School of Medicine, Aurora, CO;* ^*3*^*Pediatric Renal Section, University of Colorado School of Medicine, Aurora, CO;* ^*4*^*Center for Acute Care Nephrology, Cincinnati Children’s Hospital Medical Center, Cincinnati, OH*

**Background:** Children suffer from acute kidney injury (AKI) and associated morbidity and mortality after cardiac surgery and lack treatment options other than supportive care. Complement activation is implicated in AKI pathogenesis and associated with AKI in adults after cardiac surgery^1^ and in a heterogeneous cohort of critically ill children^2^. We aimed to test the association between urine complement factor Ba and AKI development in children with and without AKI after cardiac surgery.

**Methods:** A biorepository of urine from children who underwent cardiac surgery was leveraged. Children who received nephrotoxins or had preexisting renal insufficiency were excluded. Pre-operative and post-operative urine samples were analyzed by ELISA for the Ba fragment of complement factor B. The primary outcome measure was AKI, defined as ≥50% increase in serum creatinine (sCr) from pre-operative baseline. Repeated measures analyses with mixed effects models were performed to compare urine Ba (log value) between those with and without AKI and assessed the difference at each timepoint.

**Results:** 23 children had no AKI and 17 children developed AKI based on sCr diagnosis at 1-3 days after cardiac surgery. There was no difference in median pre-operative urine Ba levels between patients with AKI (median 24 ng/mL [IQR 12, 56]) and without AKI (34ng/mL [17,96]). Median urine Ba peaked at 6 hours after surgery in patients with AKI compared to patients without AKI (694 ng/mL [126, 1216] v. 84 ng/mL [47, 240], p 0.029). Median urine Ba remained significantly elevated at 12 hours (375 ng/mL [179,979] v. 135 ng/mL [89, 217], p 0.017), 24 hours (244 ng/mL [179, 656] vs. 127ng/mL [41, 203]; p 0.025), and 48 hours (248ng/mL [186, 1032] v. 95ng/mL [30, 319]; p 0.017) after surgery in patients with AKI compared to patients without AKI. Figure 1 shows urine Ba compared to previously measured AKI biomarkers of urine NGAL^3^ and urine IL-18^4^ in the same population.

**Conclusions:** Urine Ba is a novel biomarker that precedes the rise of sCr. Urine Ba may identify patients to optimally study complement targeted therapeutics in children at risk of AKI.

**Keywords:** Complement, acute kidney injury, cardiac surgery

**Disclosures:** This work was supported by National Institutes of Health Grants Eunice Kennedy Shriver Institute of Child Health & Human Development K12 HD 047349(EKS), R01DK076690 (JMT), R01DK125823 (JMT), and R01DK130255 (JMT and JK). Dr. Dixon is a consultant for Apellis and Alexion Pharmaceuticals, Inc. Dr. Thurman received royalties from Alexion Pharmaceuticals, Inc. and is a consultant for Q32 Bio, Inc., a company developing complement inhibitors. He also holds stock and will receive royalty income from Q32 Bio, Inc. Dr. Devarajan is a consultant for BioPorto. The remaining authors declare they have no financial interests.

**Corresponding Author:** Erin K Stenson, ORCID iD: 0000-0001-9878-5923

**References:**

1. Laskowski J, Thiessen Philbrook H, Parikh CR, Thurman JM. Urine Complement Activation Fragments are Increased in Patients with Kidney Injury After Cardiac Surgery. *Am J Physiol Renal Physiol.* 2019.

2. Stenson EK, You Z, Reeder R, et al. Complement activation fragments are increased in critically ill pediatric patients with severe acute kidney injury. *Kidney360.* 2021:10.34067/KID.0004542021.

3. Mishra J, Dent C, Tarabishi R, et al. Neutrophil gelatinase-associated lipocalin (NGAL) as a biomarker for acute renal injury after cardiac surgery. *Lancet.* 2005;365(9466):1231-1238.

4. Parikh CR, Mishra J, Thiessen-Philbrook H, et al. Urinary IL-18 is an early predictive biomarker of acute kidney injury after cardiac surgery. *Kidney Int.* 2006;70(1):199-203.

**Figure 1:** Pattern of elevation of urine biomarkers including urine Ba, urine NGAL, and urine IL-18 after cardiac surgery in children. AKI was defined as a ≥ 50% increase in serum creatinine (sCr) above baseline and developed at 48-72 hours in the patients with AKI. * = significance at p<0.05. 
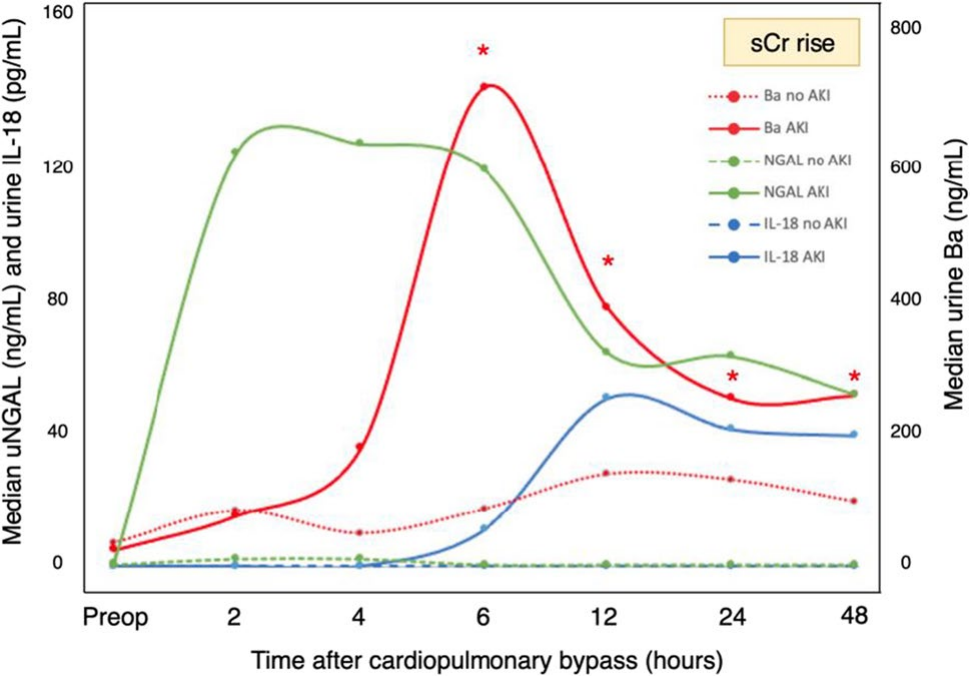


**Abstract 08**

**Epidemiology of Acute Kidney Injury Recovery Phenotypes in Non-ICU Pediatric Hospital Admissions**

Abby Basalely^1^ MD MS, Taylor Hill Horowitz^1^ BS, Meredith Akerman^2^ MS, Shupti Biswas^1^ BS, Matthew Schuchman^1^, Christine Sethna^1^ MD, Katja M Gist^3^ DO, MSc

^*1*^ *Cohen Children’s Medical Center of Northwell Health, New Hyde Park, NY;* ^*2*^*NYU Langone Hospital -Long Island, Mineola, NY;* ^*3*^*Cincinnati Children’s Hospital, Cincinnati, Ohio*

**Background:** Reports of AKI recovery patterns in adults and critically ill children demonstrate that longer AKI duration is associated with adverse short and long-term sequelae [1,2,3]. While 65% of pediatric AKI occurs outside of the ICU, AKI recovery patterns are unknown. The purpose of this study is to describe the epidemiology of AKI recovery phenotypes in non-critically ill children.

**Methods:** We performed a retrospective analysis of patients 1mo -21y with >2 serum creatinine values, admitted to 3 pediatric hospitals of the Northwell health system from January 2012-December 2020. Patients with AKI were included. AKI was defined using the KDIGO serum creatinine criteria. Only the first episode of AKI during the admission was evaluated. Patients with chronic kidney disease or dialysis dependence, kidney transplant and pregnancy were excluded. AKI recovery phenotypes were classified into four groups (1) Unclassified, unresolved AKI - AKI <48 hours without follow-up creatinine to define resolution (2) Transient AKI (tAKI), resolved within 48hrs, (3) Persistent AKI (pAKI), AKI >48hours & < 7 days (4) Acute Kidney Disease (AKD), AKI > 7days. Patients were censored at time of discharge.

**Results:** 9,515 unique patients with 14,600 admissions were analyzed. Median age was 11.1 years (IQR 3.8, 16.3) and half (N = 4,759) were female. AKI occurred in 26.5% of admissions (N=3,873), (Figure 1). Recovery pattern was unclassified in 20% (N=847). tAKI occurred in 42% (N=1627), pAKI in 28% (N=1,085) with a median duration of 73.8 hours (IQR 57.7, 105.9). AKD occurred in 8% (N=314). with a median duration of 255.2hrs (IQR 202.9, 383). Of all AKI 61% remained unresolved (N=2393) at the end of admission.

**Conclusions:** This is the first study to characterize pediatric AKI recovery phenotypes in non-ICU hospitalizations. AKI in this cohort was common and like previous findings, patients are often discharged with unresolved AKI [1]. Future directions include evaluating the associations of patterns of recovery with short-term sequelae and identifying those who need close outpatient follow-up care.

**Keywords:** AKI, AKD, Recovery Patterns

**Disclosures:** The authors declared no competing interests.

**Corresponding Author:** Abby, M, Basalely, https://orcid.org/0000-0002-5994-3658

**References**

1. Bahatraju J, et al. *JAMA Netw Open*. 2020;3(4): e202682 http:// doi:10.1001/ jamanetworkopen.2020 .2682

2. Parikh RV, et al. *Pediatrics*. 2020;146(3):e20192821. http://10.1542/peds.2019-2821

3. Lobasso M, et al. *Pediatr Nephrol* **37,** 659–665 (2022) http://10.1007/s00467-021-05179-

**Figure 1.** Schematic of Cohort and AKI Recovery Patterns 
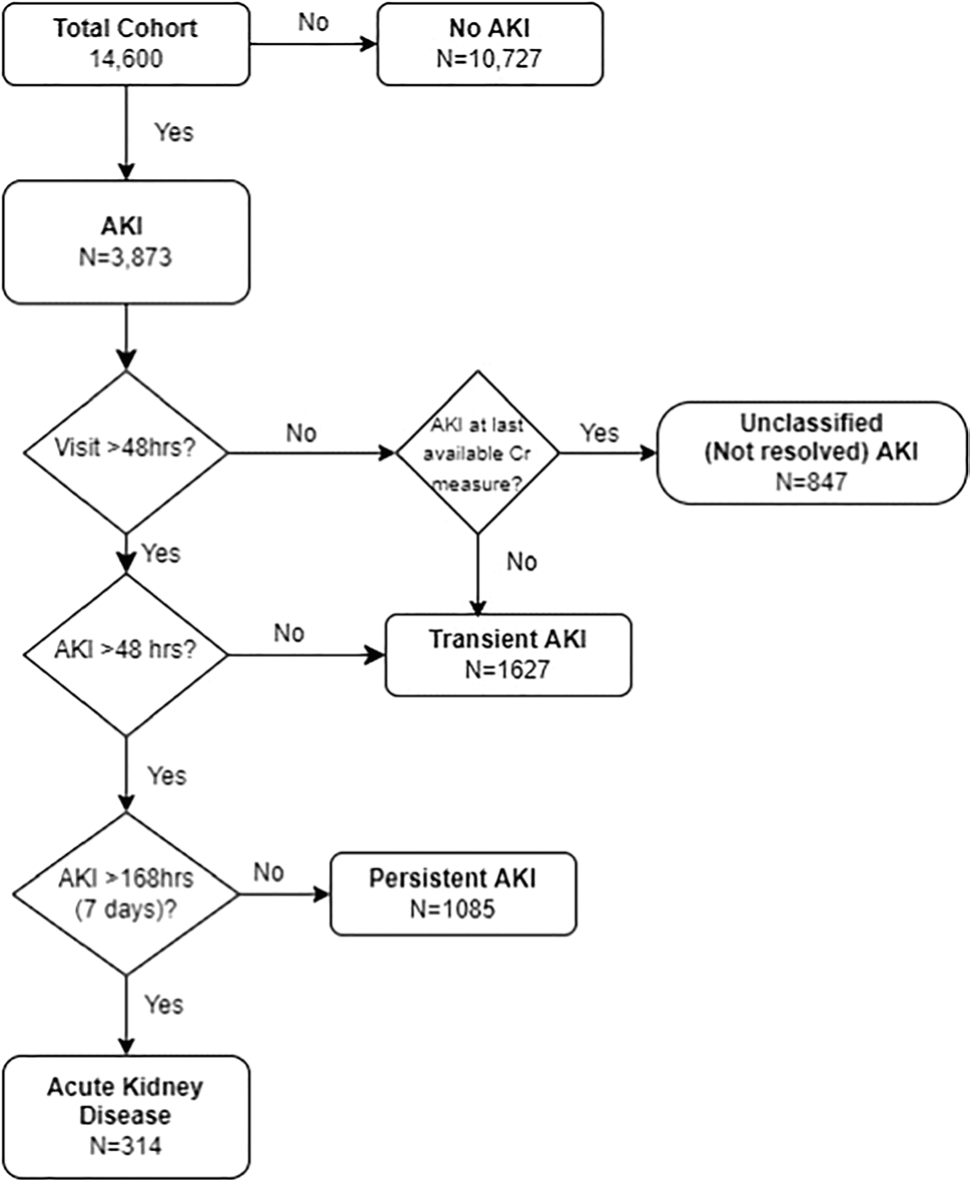


**Abstract 09**

**Peritoneal Catheters in Neonates after Complex Heart Surgery: A Multicenter Study**

David M. Kwiatkowski^1^, Jeffrey Alten^2^, Natasha S. Afonso^3^, Matthew T. Coghill^4^, David S. Cooper^2^, Joshua D. Koch^5^, Catherine D. Krawczeski^6^, Kenneth Mah^1^, Tara Neumayr^7^, Fazlur Rahman^4^, Tia T. Raymond^8^, Garret Reichle^9^, David Selewski^10^, Sarah Tabbutt^11^, Tennille Webb^4^, Santiago Borasino^4^

^*1*^ *Stanford University School of Medicine, CA;* ^*2*^*Cincinnati Children’s Hospital Medical Center, OH;* ^*3*^*Texas Children’s Hospital, TX;* ^*4*^*University of Alabama at Birmingham School of Medicine, AL;* ^*5*^*Phoenix Children’s Hospital, AZ;* ^*6*^*Nationwide Children’s Hospital, OH;* ^*7*^*Washington University School of Medicine, MO;* ^*8*^*Medical City Children’s Hospital, TX;* ^*9*^*University of Michigan C.S. Mott Children’s Hospital, MI;* ^*10*^*Medical University of South Carolina, SC;* ^*11*^*University of California San Francisco Benioff Children’s Hospital, CA*

**Introduction:** Prophylactic peritoneal dialysis (prophylactic dialysis) and passive peritoneal drainage (drainage) without dialysis are used to prevent fluid overload in neonates after complex cardiac surgery in some centers. The Neonatal and Pediatric Heart Renal Outcomes Network (NEPHRON) is a multicenter collaborative formed to investigate acute kidney injury (AKI) after neonatal cardiac surgery. We proposed a preliminary investigation comparing neonates following high-complexity cardiac surgery with no peritoneal catheter to those with prophylactic dialysis and those with drainage.

**Methods:** Twenty-two Pediatric Cardiac Critical Care Consortium (PC^4^) centers participated in the NEPHRON data module between 9/2015 and 1/2018. Among neonates undergoing Society of Thoracic Surgeons (STS) STAT 5 cardiopulmonary bypass surgery, variables and outcomes were compared among 3 cohorts: (1) prophylactic dialysis, (2) drainage, and (3) no peritoneal catheter placement. Neonates undergoing peritoneal dialysis to treat AKI were excluded. Univariate results are presented.

**Results:** Among the 378 identified neonates, 145 (38%) received a peritoneal catheter intraoperatively, of whom 53 (14%) underwent prophylactic dialysis and 92 (24%) passive drainage. The median time to prophylactic dialysis initiation was 1.9 (0, 3.5) hours with a duration of 83 (51,130) hours. The timeframe of passive drainage was not captured. Table 1 demonstrates univariate comparisons between prophylactic dialysis, passive drainage, and no peritoneal catheter. Peak percent fluid overload (mL/kg) was not different among cohorts: PPD: 4 (-1, 8); drainage: 5 (1, 8); no catheter: 3 (-1, 8). The prophylactic dialysis cohort had significantly lower duration of postoperative mechanical ventilation compared to the drainage cohort (120 vs. 160 hours, p=0.009), but not compared to the no catheter cohort (140 hours). There was no significant difference among the cohorts in duration of hospital stay or time to first negative daily fluid balance. Aggregate serum creatinine on POD 4-6 was significantly lower for both the PPD and passive drainage cohorts when compared to the no catheter cohort. There was no difference in recorded adverse events.

**Conclusions:** In the NEPHRON collaborative, in neonates undergoing STS STAT 5 surgery, peritoneal catheters were placed commonly. Prophylactic dialysis was associated with a shorter time to extubation compared to passive drainage. Otherwise, no difference in strategies was noted. Next steps include a risk adjusted multivariable analysis including center as a variable and mortality as an outcome.

**Abstract 010**

**Neonatal Nephrotoxic Medication Exposure and Early Acute Kidney Injury: Results from the AWAKEN Study**

Heidi J. Steflik^1*^, MD, MSCR, Jennifer Charlton^2^, MD, Meagan Briley^3^, MD, David T. Selewski^1^, MD, MSCR, Katja M. Gist^4^, DO, MSC, Mina H. Hanna^5^, MD, David Askenazi^6^, MD, MSPH, Russell Griffiin^6^, PhD on behalf of the Neonatal Kidney Collaborative

^*1*^ *Medical University of South Carolina, Charleston, SC, USA;* ^*2*^*University of Virginia, Charlottesville, VA, USA;* ^*3*^*Vanderbilt University, Nashville, TN, USA;* ^*4*^*University of Cincinnati, Cincinnati Children’s Hospital Medical Center, Cincinnati, OH, USA;* ^*5*^*University of Kentucky, Lexington, KY, USA;* ^*6*^*University of Alabama at Birmingham, Birmingham, AL, USA*

**Background:** Nephrotoxic medications (NTXm) exposures and associations with acute kidney injury (AKI) in critically ill neonates remain understudied. We aimed to describe the epidemiology of NTXm exposure and investigate associations between NTXm and AKI in the first postnatal week (early AKI) in the neonatal intensive care unit (NICU) utilizing a large, multicenter cohort.

**Methods:** In a secondary analysis of the AWAKEN cohort [1], NTXm receipt (acyclovir, amphotericin B, aminoglycosides (AG), piperacillin-tazobactam, vancomycin, ibuprofen, indomethacin) during the first postnatal week was recorded. NTXm exposure was quantified: no NTxm, NTXm excluding AG, AG alone, AG with another NTXm. Associations with early AKI (stage 1 or severe (stage 2 or 3) AKI), defined by the modified neonatal KDIGO creatinine criteria, were evaluated using time-varying Cox proportional hazard regression models.

**Results:** Of 2162 neonates, 75% were exposed to NTXm within the first postnatal week (no NTXm 25.3%, NTXm excluding AG 2.9%, AG alone 60.8%, AG with another NTXm 11.1%). AG’s were the most commonly administered NTXm (72%). When examining NTXm exposure by gestational age (22-28 weeks, 29-35 weeks, >36 weeks), neonates born at 22-28 weeks of gestation were most frequently exposed to each NTXm (except acyclovir and piperacillin-tazobactam).

Early AKI occurred in 21% and was associated with NTXm exposure (no NTXm 19.2%, NTXm excluding AG 35.5%, AG alone 19.5%, AG with another NTXm 30.5%; p<0.01). Stage 1 AKI (80.3%) was most common. After adjusting for confounders, NTXm excluding AG receipt was associated with increased risk of any early AKI (HR 3.14, 95% CI 1.31-7.55); AG with another NTXm receipt was associated with increased risk of severe early AKI (HR 4.79, 95% CI 2.19-10.50). In neonates with data available beyond day 3 of life, all NTXm exposure groups were independently associated with increased risk of any early AKI; AG alone and AG with another NTXm receipt were both independently associated with increased risk of severe early AKI (Table).

**Conclusions:** During the first postnatal week, NTXm exposure in the NICU was frequent. AG receipt was most common. Specific NTXm exposure, principally AG with an additional NTXm, was independently associated with early AKI in critically ill neonates.

**Keywords:** nephrotoxic medication, acute kidney injury, renal failure

**Disclosures:** HJS receives grant funding from Baxter. JC is a Medtronic consultant. DA consults and/or receives funding for education/research from Baxter, Nuwellis, Medtronic, Seastar, Bioporto, Portero, CSO and founder of Zorro-Flow, Inc. The other co-authors declare no conflicts of interest or disclosures.

**Corresponding author:** Heidi J. Steflik, https://orcid.org/0000-0003-2168-4926

**References**

1. Jetton JG, et al. *Lancet Child Adolesc Health* 2017 Nov;1(3):184-194. http://doi.org/10.1016/S2352-4642(17)30069-X

**Table.** Crude and Adjusted Hazards Ratios* (HRs) and Associated 95% Confidence Intervals (CIs) for the Association between Nephrotoxic Medication (NTXm) Exposure and Acute Kidney Injury (AKI)

**Table:** 

Outcomes	**No NTXm** **(n=546)**	**NTXm,** **excluding AG** **(n=62)**	**AG alone** **(n=1315)**	**AG and** **another NTXm** **(n=239)**
OVERALL				
Any Stage AKI				
N, %	105 (19.2)	22 (35.4)	256 (19.5)	73 (30.5)
Crude HR (95% CI)	Referent	2.65 (1.13-6.23)	1.26 (0.88-1.80)	**1.80 (1.03-3.13)**
Adjusted HR (95% CI)	Referent	**3.14 (1.31-7.55)**	1.25 (0.86-1.82)	1.68 (0.95-2.97)
Severe (Stage II or III) AKI				
N, %	15 (14.3)	4 (18.2)	52 (20.3)	19 (26.0)
Crude HR (95% CI)	Referent	2.28 (0.52-9.97)	1.57 (0.88-2.79)	**4.56 (2.14-9.72)**
Adjusted HR (95% CI)	Referent	2.51 (0.56-11.36)	1.74 (0.94-3.21)	**4.79 (2.19-10.50)**
NEONATES WITH ≥3 DAYS FOLLOW-UP				
Any Stage AKI				
N, %	91 (17.3)	19 (32.2)	203 (16.1)	49 (22.8)
Crude HR (95% CI)	Referent	**3.00 (1.03-8.78)**	**1.85 (1.14-3.01)**	**2.68 (1.37-5.24)**
Adjusted HR (95% CI)	Referent	**3.54 (1.18-10.64)**	**1.92 (1.16-3.19)**	**2.68 (1.35-5.33)**
Severe (Stage II or III) AKI				
N, %	5 (5.3)	3 (14.3)	36 (15.1)	12 (18.2)
Crude HR (95% CI)	Referent	1.58 (0.20-12.51)	2.00 (0.96-4.18)	**4.18 (1.66-10.53)**
Adjusted HR (95% CI)	Referent	2.12 (0.25-17.74)	**2.65 (1.16-6.06)**	**5.38 (1.99-14.56)**

* Estimated from a Cox proportional hazards regression stratified by gestational age category (22-28, 29-35, and ≥36 weeks) with NTXm exposure entered as a time-varying covariate

† Adjusted for 5-minute APGAR, continuous gestational age, birthweight, maternal hypertension, bacterial infection, out-born delivery, mode of delivery, and neonatal sex, race, and ethnicity

**Abstract 011**

**Characterization of C–C motif chemokine ligand 14 (CCL14) for prediction of persistent AKI in critically ill children.**

Katie Brandewie, Jeffrey Alten, Stuart Goldstein, Kat Gist

Acute kidney injury (AKI) is a common problem affecting more than 25% of critically ill children, including those following cardiac surgery. Until recently, there was limited data on the temporal associations of AKI with outcomes. Indeed, AKI lasting a brief period (24-48 hours) was considered the same as an AKI episode lasting more than 48 hours. Fortunately, most episodes of AKI in critically ill children resolve quickly. However, in approximately 5-10% of critically ill children, AKI persists. Persistent AKI is defined as AKI lasting longer than 72 hours and is a risk factor for long-term sequala, including chronic kidney disease, infection/sepsis, and death. It remains difficult to modify the clinical course of patients with AKI, which may be related to the inability to identify those at risk for persistent injury. Recent risk stratification tools such as the renal angina index and cardiac renal angina index have been developed to identify children at risk for severe day 3 AKI when assessed early in the intensive care unit course. Unfortunately, these tools do not identify children who are at risk for persistent AKI. **There is a significant knowledge gap in identifying patients who are at risk for persistent AKI.**

Urinary C-C motif chemokine ligand 14 (CCL14) is a small molecule member of the chemokine family that plays a role in leukocyte chemotaxis and is involved in tissue injury and repair processes. CCL14 is an important chemokine for monocyte and macrophage recruitment, both of which are believed to play important roles in kidney tissue damage and development of persistent kidney dysfunction. Furthermore, CCL14 has been shown to be an inflammatory marker identifying the risk of developing end-stage renal disease in diabetics. Recently, urinary C-C motif chemokine ligand 14 (CCL14) was identified as a biomarker predictive of persistent AKI in critically ill adults. CCL14 outperformed other biomarkers measured in the serum and urine for the prediction of stage 3 persistent AKI with an AUC of 0.83. Similarly, risk for renal replacement therapy and/or death at 90 days increased with tertiles of CCL14 concentration. **Whether CCL14 predicts persistent severe AKI in critically ill children is unknown.** The performance of CCL14 in critically ill children will be evaluated in the following specific aims:

**Aim 1.** To determine the performance of CCL14 for predicting persistent severe acute kidney injury in critically ill children undergoing cardiac surgery.

**Hypothesis 1.** CCL14 will be highly predictive of persistent severe AKI in children undergoing cardiac surgery.

**Approach:** All patients who have consented to the Heart Institute biorepository will be considered for inclusion. Study patients will be included if they have moderate to severe AKI (KDIGO stage 2 or 3 as defined by urine output or creatinine) based on a preoperative baseline for creatinine or urine output criteria. Control patients will be included as patients without AKI who are matched 1:1 by age and surgical complexity (by STAT category) to study patients. Patients without a urine sample within 36 hours of CS-AKI diagnosis will be excluded. One urine sample from within the first 36 hours for each included patient will be analyzed for CCL14. The performance of CCL14 will be evaluated by determining the sensitivity, specificity, NPV, PPV and AUC. Persistent severe AKI will be defined as: 1) stage 2 or 3 AKI which lasts ≥ 72 hours or 2) death with 30 days of cardiac surgery or 3) stage 2 or 3 with initiation of kidney replacement therapy within 7 days. These criteria have been modified from the original adult studies based on several factors: 1) mortality rates in pediatric cardiac surgery are low, even with AKI, 2) initiation of kidney replacement therapy in children is generally later than in adults, including that mortality rates are urine samples will be collected within 36 hours of stage 2 or 3 AKI. This study will serve as a pilot and feasibility study to provide preliminary data for future prospective studies.

**Aim 2.** To determine the performance of CCL14 for predicting persistent severe acute kidney injury in critically ill children admitted to the pediatric intensive care unit.

**Hypothesis 2.** CCL14 will be highly predictive of persistent severe AKI in critically ill children.

**Approach:** This will be a secondary analysis of the AKI-CHERUB (Acute kidney injury in children expected by renal angina and urinary biomarkers) study. Study patients will be included if they have moderate to severe AKI (KDIGO stage 2 or 3 as defined by urine output or creatinine) based on a preoperative baseline for creatinine or urine output criteria. Control patients will be included as patients without AKI who are matched 1:1 by age and surgical complexity (by STAT category) to study patients. Patients without a urine sample within 36 hours of moderate to severe AKI diagnosis will be excluded. One urine sample from within the first 36 hours for each included patient will be analyzed for CCL14. The performance of CCL14 will be evaluated by determining the sensitivity, specificity, NPV, PPV and AUC. Persistent severe AKI will be defined as: 1) stage 2 or 3 AKI which lasts ≥ 72 hours or 2) death with 30 days of cardiac surgery or 3) stage 2 or 3 with initiation of kidney replacement therapy within 7 days. Similar to aim 1, this is a pilot and feasibility aim, from which prospective studies can be developed and the findings validated.

**Abstract 012**

**Understanding potential social determinants of health in non-ICU children exposed to high nephrotoxic medication**

Jessica Hicks^1*^, MPH, Michelle Cooley^1^, MSHQS, CPHQ, John Andrew Young^1^, MPH, Amy Hobbs^1^, BSN, RN, Kara Short^1^, MSN, CRNP, David Askenazi^1,2^, MD, MsPH

^*1*^ *Children’s of Alabama, Birmingham, AL, USA;* ^*2*^ *University of Alabama at Birmingham, Birmingham, AL, USA*

**Background:** Social determinants of health (SDH) influence patient outcomes and can be more important than health care, clinical decisions, or lifestyle choices in impacting health. Nephrotoxic Injury Negated by Just-in-Time Action (NINJA) is an ongoing Quality Improvement initiative that aims to avoid serious harm from nephrotoxic medications (NTM). To our knowledge, the differences in the SDH have not been evaluated in the NINJA program.

**Methods:** Children’s of Alabama (COA) has participated in the NINJA collaborative since October 2014. For this study, we evaluated 1613 non-ICU children exposed to high NTM from January 2019 to June 2022 as part of our organization’s priority effort to identify disparities in SDH across Hospital Acquired Conditions. We examined 4 SDH: race, ethnicity, gender, and payor, additionally age. Chi-square, Student’s t-test, or Poisson Rate test were used as appropriate. A p-value <0.05 indicates statistical significance.

**Results:** Differences between groups were identified by comparing performance in a group of interest to that in a reference group (Table 1). Compared to Whites, African American/Blacks had higher NTM Exposure rate (p= 0.002). Compared to Non-Hispanics, Hispanics had lower SCr Adherence (p=0.001). Males had higher High NTM Exposure rates (p= 0.002).

Compared to patients with private insurance payor, those with non-Private Payor had lower High NTMx Rate (p<0.001), AKI Prevalence (p<0.001), NTM-AKI (p=0.003), AKI Days (p=0.003), and SCr Adherence (p<0.001).

Compared to 7-12 years, High NTM Exposure rate was lower in patients <1 year (p< 0.001) and >=13 years (p< 0.001) while AKI Prevalence was lower in <1 year (p<0.001), 1-2 years (p<0.001), and 3-6 years (p<0.05); The High NTM-AKI rate was lower in <1 year ( p<0.001), 1-2 years (p<0.001), 3-6 years (p= 0.011), and higher in >=13 years (p < 0.001). AKI Days were lower in <1 year (p<0.001). The SCr Adherence was lower in <1 year (p<0.001), 1-2 years (p= 0.012), and 3-6 years (p<0.001).

**Conclusion:** We report differences in outcomes and treatment of patients exposed to NTM that merit further exploration and action. Studies designed to uncover etiologies of these differences are needed to delineate equitable interventions necessary to address SDH as contributors to clinical outcomes.

**Keywords:** Social determinants of health, Nephrotoxic medication exposure

**Disclosures: **The authors report No COI directly associated with this abstract.

For full transparency, David J Askenazi reports consulting/grants from Baxter, Nuwellis, Medtronic, Seastar, Bioporto and Portero. He is the CSO and founder of Zorro-Flow Inc.

**Table 1. Baseline Characteristics of Population and Comparison of SHD NINJA Metrics** 
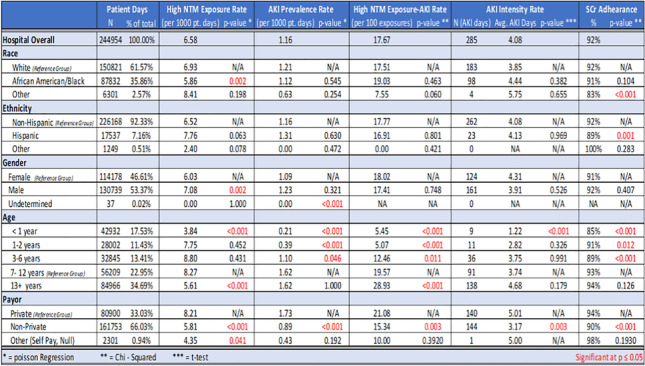


Other Race: American Indian or Alaskan Native, Asian or Pacific Islander, Hispanic, Multi Racial, Native Hawaiian Pacific Islander, NULL, Other Race, Patient Declined, Undetermined, Unknown.

**Abstract 013**

**Early postoperative weight-based fluid overload is associated with worse outcomes after neonatal cardiac surgery: a report from the multicenter NEPHRON collaborative**

Katie L Brandewie^1^, MD, David K Bailly^2^, DO, Priya N Bhat^3^, MD, MS, John W Diddle^4^, MD, Muhammad Ghbeis^5^, MD, Catherine D Krawczeski^6^, MD, Kenneth E Mah^7^, MD, MS, Tara M Neumayr^8^, MD, Tia T Raymond^9^, MD, Garrett Reichle^10^, MS, David T Selewski^11^, MD, Huaiyu Zang^1^, PhD, Jeffrey A Alten^1^, MD

^*1*^ *Division of Pediatric Cardiology, Heart Institute, Cincinnati Children’s Hospital Medical Center, Department of Pediatrics;* ^*2*^*Division of Pediatric Critical Care, University of Utah, Salt Lake City, Utahl* ^*3*^*Department of Pediatrics, Sections of Critical Care Medicine and Cardiology, Texas Children's Hospital and Baylor College of Medicine, Houston, Texas;* ^*4*^*Children’s National Hospital, Division of Cardiac Critical Care Medicine, Washington, DC;* ^*5*^*Division of Cardiovascular Critical Care, Department of Cardiology, Boston Children’s Hospital, Harvard Medical School;* ^*6*^*Nationwide Children’s Hospital, The Ohio State University College of Medicine;* ^*7*^*Stanford University School of Medicine, Palo Alto, CA;* ^*8*^*Washington University School of Medicine, Department of Pediatrics, Divisions of Pediatric Critical Care Medicine and Pediatric Nephrology, St. Louis, MO;* ^*9*^*Pediatric Cardiac Intensive Care, Medical City Children’s Hospital, Dallas, TX;* ^*10*^*CS Mott Children’s Hospital, University of Michigan, Ann Arbor, Michigan;* ^*11*^*Department of Pediatric, Medical University of South Carolina.*

Corresponding author: Katie L Brandewie, MD, Division of Pediatric Cardiology, Heart Institute, Cincinnati Children’s Hospital Medical Center, 3333 Burnet Avenue, Cincinnati, OH 45229, Phone: (859) 351-8409, Email: katie.brandewie@cchmc.org

Financial support: Cincinnati Children’s Hospital Medical Center Heart Institute Research Core, Castin’ ‘N Catchin’ Charity Organization

Financial disclosures and conflicts of interest: none

**ABSTRACT**

**Objectives:** Evaluate the association of postoperative day (POD) 2 weight-based fluid balance (FB-W) >10% with outcomes after neonatal cardiac surgery.

**Design:** Retrospective cohort study.

**Setting:** 22 hospitals in the NEonatal and Pediatric Heart and Renal Outcomes Network (NEPHRON) registry from September 2015 to January 2018.

**Patients:** Consecutive neonates (<30 days) undergoing index cardiac operation with/without cardiopulmonary bypass (CPB).

**Interventions:** None.

**Measurements and Main Results:** Of 2240 eligible patients, only 997 neonates (CPB n=658, non-CPB n=339) were weighed on POD2 and included in this analysis. Forty-five percent (n=444) of patients had FB-W >10%. In bivariable analysis, patients with POD2 FB-W >10% had higher acuity of illness, more resource utilization and worse outcomes. Hospital mortality was 2.8% (n=28) and not independently associated with POD2 FB-W >10% (OR 1.04, 95% CI 0.29-3.68). POD2 FB-W >10% was associated with all utilization outcomes, including duration of mechanical ventilation (multiplicative rate of 1.19, 95% CI 1.04-1.36), respiratory support (1.28, 95% CI 1.07-1.54), inotropic support (1.38, 95% CI 1.10-1.73), and postoperative hospital length of stay (1.15, 95% CI 1.03-1.27). In secondary analyses, POD2 FB-W as a continuous variable also demonstrated association with prolonged durations of all utilization outcomes, including duration of mechanical ventilation (OR 1.04, 95% CI 1.02-1.06], respiratory support (1.03, 95% CI 1.01-1.05), inotropic support (1.03, 95% CI 1.00-1.05), and postoperative hospital length of stay (1.02, 95% CI 1.00-1.04). POD2 intake-output based fluid balance (FB-IO) was not associated with any outcome.

**Conclusions:** POD2 weight-based fluid balance >10% occurs frequently after neonatal cardiac surgery and is associated with longer cardiorespiratory support and postoperative hospital length of stay. However, POD2 FB-IO was not associated with clinical outcomes. Initiatives to prevent and mitigate early postoperative fluid accumulation could provide opportunities to improve outcomes but would require efforts to increase the proportion of neonates safely weighed in the early postoperative period.

**Abstract 014**

**Fluid Balance and Short- and Long-Term Respiratory Outcomes in Extremely Premature Neonates: Results from the PENUT study**

Michelle C. Starr^1*,2^, Russell Griffin^3^, Katja M. Gist^4^, Jeffrey L. Segar^5^, Rupesh Raina^6^, Ronnie Guillet^7^, Saudamini Nesargi^8^, Shina Menon^9^, Nekayla Anderson^3^, David Askenazi^3^, David T. Selewski^10^ on behalf of the Neonatal Kidney Collaborative Research Committee

^*1*^ *Indiana University School of Medicine, Indianapolis, IN, USA;* ^*2*^*Pediatric and Adolescent Comparative Effectiveness Research, Indiana University School of Medicine, Indianapolis, IN, USA;* ^*3*^*University of Alabama at Birmingham, Birmingham, AL, USA;* ^*4*^*Cincinnati Children’s Hospital Medical Center, University of Cincinnati, Cincinnati, OH, USA;* ^*5*^*Medical College of Wisconsin, Milwaukee, Wisconsin, USA;* ^*6*^*Akron Children's Hospital, Akron, OH, USA;* ^*7*^*Golisano Children's Hospital, University of Rochester, NY, USA; St. Johns Medical College Hospital, Bangalore, Karnataka, India;* ^*9*^*University of Washington and Seattle Children's Hospital, Seattle, WA, USA;* ^*10*^*Medical University of South Carolina, Charleston, SC*

**Background:** Extremely low gestational age neonates (ELGANs) are at risk of acute kidney injury (AKI) and disorders of fluid balance (FB). Few data exist on the association between FB and respiratory outcomes in this population.

**Methods:** We evaluated neonates born 24–27 weeks gestation enrolled in the PENUT study, a Phase III randomized, placebo-controlled trial in 30 NICUs in the United States from 12/2013-9/2016. Primary exposure: peak FB in the first 14 postnatal days. Secondary exposures: FB at postnatal day 3 and 7. FB was calculated as percent change in weight from birthweight. Primary outcome: mechanical ventilation on postnatal day 14. Composite secondary outcome: severe bronchopulmonary dysplasia (BPD) or death.

**Results:** 923 preterm neonates were evaluated. 480/923 (53.5%) were mechanically ventilated on postnatal day 14 and 554/923 (60.0%) had severe BPD/death. Neonates with peak FB >5% had 1.75 higher odds (95% CI 1.33, 2.31, *p*<0.0001) of requiring mechanical ventilation on postnatal day 14, and 1.51 higher odds (95% CI 1.11, 2.06, *p*=0.009) of severe BPD/death. Neonates with FB >10% had 1.74 higher odds (95% CI 1.41, 2.15, *p*<0.0001) of requiring mechanical ventilation on postnatal day 14 and 1.51 higher odds (95% CI 1.11, 2.06, *p*=0.009) of BPD/death. After adjusting for confounding variables, for every 5% increase in peak fluid balance there was 2.21 higher odds of mechanical ventilation on postnatal day 14 (aOR 2.21, 95% CI: 1.61, 2.80; *p*<0.0001). Neonates requiring mechanical ventilation on postnatal day 14 had a less negative FB at 3 days (-5% vs.-8%, p<0.0001) than infants not requiring mechanical ventilation at postnatal day 14 (**Table 1**).

**Conclusions:** Peak FB was associated with mechanical ventilation on postnatal day 14, BPD and mortality. FB at DOL 3 is a potential early marker of poor respiratory outcomes. The extent to which FB is causative in development of poor outcomes remains unclear. Future work should determine if interventions including careful monitoring of weights and targeted fluid management improve patient outcomes.

**Keywords:** Neonatal kidney disease, fluid overload, mechanical ventilation, bronchopulmonary dysplasia.

**Disclosures:** The authors declared no competing interests.

**Corresponding Author:** Michelle C. Starr, https://orcid.org/0000-0001-9412-8950

**References:** None

**Table 1.** Fluid balance stratified by mechanical ventilation on postnatal day 14

**Table:** 

		Postnatal day 14 Mechanical Ventilation		p-value
Fluid Exposure	Cohort (n=874)	Yes (n=458)	No (n=415)	
Peak Fluid Balance First 14 d	11% (4%, 20%)	15% (8%, 24%)	8% (2%, 14%)	<0.0001
Fluid Balance at Postnatal Day 3	-7% (-12%, 0%)	-5% (-11%, 0%)	-8% (-12%, -3%)	<0.0001
Fluid Balance at Postnatal Day 7	-3% (-8%, 4%)	-4% (-9%, 2%)	-1% (-7%, 7%)	<0.0001

* Estimated from a Wilcoxon rank sums test

**Abstract 015**

**Acute kidney injury in preterm neonates is associated with lower cerebral tissue oxygenation**

Matthew W. Harer^1^, Kari Borowski^1^, Claudette Adegboro^1^

^*1*^ *University of Wisconsin School of Medicine and Public Health, Madison, WI, USA*

**Background:** The kidney and central nervous system are strongly interconnected [1]. In preterm neonates**,** acute kidney injury (AKI) has been independently associated with increased rates and severity of intraventricular hemorrhage (IVH) [2]. Similarly, children with chronic kidney disease (CKD) have been shown to have abnormal brain architecture and function [3]. Prior near-infrared spectroscopy (NIRS) tissue oxygenation studies in critically ill neonates have not evaluated differences in Cerebral regional Somatic Oxygenation (CrSO_2_) based on AKI status. The objective of this study was to evaluate CrSO_2_ trends in preterm neonates with and without AKI.

**Methods:** We retrospectively evaluated the CrSO_2_ values of 35 neonates born less than 32 weeks’ gestation who were prospectively enrolled in a continuous NIRS monitoring study in the first week [4]. AKI was determined by the modified neonatal Kidney Disease: Improving Global Outcomes (KDIGO) definition including urine output (UOP). Incidence of IVH and periventricular leukomalacia (PVL) were collected from head ultrasound or MRI imaging performed during the NICU hospitalization.

**Results:** Three patients developed AKI (Stage 1; n=1, Stage 2, n=2) at an average age of five days old. There was no difference in rates of IVH or PVL between the AKI and no AKI groups (AKI, n=1 (33.33%); No AKI, n=6 (18.75%), p=0.124). The median CrSO_2_ values for AKI patients were significantly lower for the first week compared to the no AKI patients (65.12% vs. 75.67%, p<0.0001). The fluctuations in median CrSO_2_ values for AKI vs. no AKI groups over time are shown in Figure 1 with the peak difference seen at 5 days of age.

**Conclusions:** In this small pilot analysis, neonates with AKI had lower cerebral oxygenation compared to those without AKI. This data highlights the possibility that cerebral tissue perfusion or oxygen utilization may be affected by AKI independent of brain injury. Larger prospective studies are needed to determine the extent to which AKI affects cerebral oxygenation as well as the timing of these changes. Future preterm neonatal NIRS and AKI studies should include short and long-term neurodevelopmental outcomes to evaluate if prevention of AKI may also protect the brain and cognitive function.

**Keywords:** Near infrared spectroscopy (NIRS), Cerebral oxygenation, Preterm, Neonate, Acute Kidney Injury (AKI)

**Disclosures:** The authors of no relevant financial disclosures. The authors had funding support from the UnityPoint Health Meriter Foundation and the Department of Pediatrics at the University of Wisconsin-Madison to support the purchase of NIRS machines and sensors for this study.

**Corresponding Author:** Matthew W. Harer, https://orcid.org/0000-0001-5493-3269

**References:**

1. Tanaka S, Okusa MD. Crosstalk between the nervous system and the kidney. Kidney Int. 2020 Mar;97(3):466-76.

2. Stoops C, Boohaker L, Sims B, Griffin R, Selewski DT, Askenazi D. The Association of Intraventricular Hemorrhage and Acute Kidney Injury in Premature Infants from the Assessment of the Worldwide Acute Kidney Injury Epidemiology in Neonates (AWAKEN) Study. Neonatology. 2019;116(4):321-30.

3. Steinbach EJ, Harshman LA. Impact of Chronic Kidney Disease on Brain Structure and Function. Front Neurol. 2022;13:797503.

4. Harer MW, Adegboro CO, Richard LJ, McAdams RM. Non-invasive continuous renal tissue oxygenation monitoring to identify preterm neonates at risk for acute kidney injury. Pediatr Nephrol. 2021 Jun;36(6):1617-25.

**Figure 1: Cerebral oxygenation by AKI Status**

In the following figure the x axis is time from birth in hours while the y axis represents cerebral oxygenation percentage. The lines represent the median while the shaded area around each line represents the interquartile range. The AKI group has the dotted line and dotted shaded region. The peak difference in the two groups occurs after 96 hours and before 144 hours. 
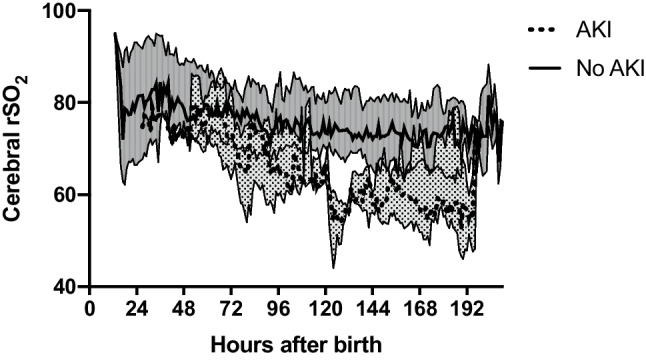


**Abstract 016**

**Investigating the association between renal tissue oxygenation and development of AKI in preterm neonates**

Matthew W. Harer^1^, Paige E. Condit^1^, Jennifer Chuck^2^, Michael R. Lasarev^1^, Valerie Chock^2^

^*1*^ *University of Wisconsin School of Medicine and Public Health, Madison, WI, USA;* ^*2*^*Stanford University, Palo Alto, CA, USA*

**Background:** Neonatal acute kidney injury (AKI) is classically defined by elevations in serum creatinine (SCr) and decreases in urine output and is associated with short- and long-term consequences [1]. Monitoring Renal regional Saturation of Oxygen (RrSO_2_) with near-infrared spectroscopy (NIRS) can potentially diagnose AKI noninvasively before changes in traditional markers of kidney function thereby creating a therapeutic window for intervention [2]. We sought to evaluate the relationship between RrSO_2_ changes and SCr during the first week of age for preterm neonates born at < 32 weeks gestational age (GA).

**Methods:** Prospectively measured RrSO_2_ collected during the first week of age in neonates at < 32 weeks GA were analyzed from two sites (A & B). Variables were compared between groups (AKI vs no AKI) using rank-sum or exact unconditional tests for continuous and categorical variables, respectively. Poisson regression was used to estimate the rate of AKI events over the duration of observation (t), which varied by patient.

**Results:** 109 neonates (32% from A and 68% from B) were included and 560 SCr values were obtained during the first week of age. Eight cases of AKI were observed in the cohort (all diagnosed with SCr) with a similar prevalence between the two sites (9% at A and 7% at B, p=0.767). For the 8 cases with AKI, the median [IQR] of their mean %RrSO_2_ was 46.2 [32.8,70.5] and for the non-AKI cases it was 67.1 [58.5, 74.0] (p=0.12). A decrease of 10 percentage points in mean %RrSO_2_ was associated with a 1.7-fold increase in AKI risk (95% CI: 1.1–2.6; p = 0.016). The association between AKI risk and mean %RrSO2 is shown in Figure 1.

**Conclusions:** Decreases in mean RrSO_2_ in neonates born at < 32 weeks GA were associated with an increased risk of AKI, like single center studies [3, 4]. Further prospective studies are necessary to determine whether RrSO_2_ changes can accurately detect AKI and correlate with urinary biomarkers of kidney injury. Future studies should focus on early interventions and therapies that can improve renal oxygenation and evaluate if these changes affect short- and long-term kidney outcomes.

**Keywords:** Near infrared spectroscopy (NIRS), Renal oxygenation, Preterm, Neonate, Acute Kidney Injury (AKI)

**Disclosures:** The authors of no relevant financial disclosures. The authors had funding support from the UnityPoint Health Meriter Foundation and the Department of Pediatrics at the University of Wisconsin-Madison to support the purchase of NIRS machines and sensors for this study.

**Corresponding Author:** Matthew W. Harer, https://orcid.org/0000-0001-5493-3269

**References:**

1. Starr MC, Charlton JR, Guillet R, Reidy K, Tipple TE, Jetton JG, et al. Advances in Neonatal Acute Kidney Injury. Pediatrics. 2021 Nov;148(5).

2. Harer MW, Chock VY. Renal Tissue Oxygenation Monitoring-An Opportunity to Improve Kidney Outcomes in the Vulnerable Neonatal Population. Frontiers in Pediatrics. 2020;8:241.

3. Bonsante F, Ramful D, Binquet C, Samperiz S, Daniel S, Gouyon J-B, et al. Low Renal Oxygen Saturation at Near-Infrared Spectroscopy on the First Day of Life Is Associated with Developing Acute Kidney Injury in Very Preterm Infants. Neonatology. 2019;115(3):198-204.

4. Harer MW, Adegboro CO, Richard LJ, McAdams RM. Non-invasive continuous renal tissue oxygenation monitoring to identify preterm neonates at risk for acute kidney injury. Pediatr Nephrol. 2021 Jun;36(6):1617-25.

**Figure 1: Renal Oxygenation and Probability of Acute Kidney Injury**

Association between mean %RrSO2 (horizontal axis) and probability of developing AKI (vertical axis). Individual mean %RrSO2 values are indicated by circular points for 8 infants who developed AKI (probability 1; top edge) and the other 101 who didn't (probability 0; bottom edge). Solid line shows increasing probability with decreasing mean %RrSO2 and 95% confidence interval as surrounding dashed lines. 
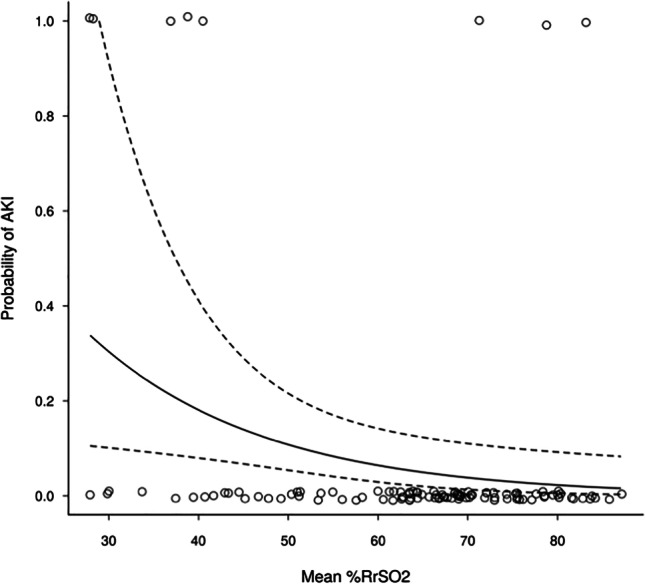


**Abstract 017**

**Development of ICONIC:** ***Improving*** ***Carpediem™*** ***Outcomes in*** ***Neonates and*** ***Infants through*** ***Collaboration***

Jolyn Morgan^1^, Kelli Krallman^1^, Amanda Snyder^1^, Shina Menon^2^, Katja Gist^1^, Catherine Joseph^3^, Jennifer Jetton^4^, Kera Luckritz^5^, Susan Martin^6^, Katie Plomaritas^5^, Cheryl L. Tran^7^, Cara L. Slagle^1*^ *on behalf of the ICONIC Collaborative*

^*1*^ *Cincinnati Children’s Hospital, Cincinnati, Ohio;* ^*2*^*Seattle Children’s Hospital, Seattle, Washington;* ^*3*^*Texas Children’s Hospital, Houston, Texas;* ^*4*^*Medical College of Wisconsin, Milwaukee, Wisconsin;* ^*5*^*University of Michigan, Ann Arbor, Michigan;* ^*6*^*Golisano Children’s Hospital, Rochester, New York;* ^*7*^*Mayo Clinic, Rochester, Minnesota*

**Background: **The CArdio-Renal PEdiatric DIalysis Emergency Machine (Carpediem™, Medtronic, United States) is a dialysis device designed to provide continuous kidney replacement therapy (CKRT) safely to neonates and infants. Understanding single center use and best practice will be incredibly challenging as we anticipate its use to be important, yet rare. Thus, we created a multicenter multidisciplinary quality improvement and research registry dedicated to informing best practices and treatment strategies to improve outcomes.

**Methods: **The collaborative was established with 7 sites that were the first to offer Carpediem™ therapy in the United States with plans to expand nationally and ultimately internationally. Specific aims of the collaborative include describing variations in education, CKRT prescription and delivery in neonates, and establishing a registry for use in quality improvement and benchmarking efforts. Multidisciplinary representation at each site is encouraged. Research aims include understanding short- and long-term outcomes with a focus on growth and development. Institutional Review Board approval was obtained with intent to enroll all patients receiving Carpediem™ at all participating sites.

**Results: **To date, 22 centers have joined ICONIC with representation including nursing leadership, pharmacists, dietitians, nephrologists, pediatric, cardiac and neonatal intensivists, advanced practice providers, and fellows. A subject database using Research Electronic Data Capture (REDCap) tools, and a web-based electronic real-time dashboard for observing collaborative and individual quality metrics have been created and are hosted by Cincinnati Children’s Hospital Medical Center. Prospective REDCap data being collected includes but is not limited to demographics and diagnosis, Carpediem™ prescription, daily fluid balance, and short- and long-term outcomes. Additionally, a biorepository is available for urine, serum, and effluent specimens. Alternating monthly meetings occur between the ICONIC steering committee composed of the initial 7 core sites and overall collaborative sites. Collectively, 10 centers have provided over 30 patients with Carpediem™ therapy. As centers become more established, we aim to analyze data for variation and identification of best practices.

**Conclusions: **Multidisciplinary, multicentered collaboration is critical to understanding Carpediem™ best practices secondary to its infrequency.

**Keywords: **Carpediem™, Neonatal CKRT

**Disclosures: ***Jolyn Morgan is a consultant for Medtronic*

*Shina Menon and Kat Gist (ICONIC Collaborative) are recipients of a Gerber Foundation Grant*

**References: **None

**Abstract 018**

**ICONIC (Improving Carpediem™ Outcomes in Neonates and Infants through Collaboration): A Survey to Understand Carpediem™ Education Practices and Care Delivery Models**

Jolyn Morgan^1*^, Amanda Snyder^1^, Katie Plomaritas^2^, Lauren Casey^3^, Pamela Heise^3^, Jennifer Jetton^4^, Susan Martin^5^, Shina Menon^6^, Melissa Muff-Luett^7^, David Ramirez^8^, Marta Suarez-Rivera^9^, Cheryl L. Tran^8^, Brynna Van Wyk^10^, Kim T. Vuong^3^, Larissa Yalon^6^, Cara L. Slagle^1^ *on behalf of the ICONIC Collaborative*

^*1*^ *Cincinnati Children’s Hospital, Cincinnati, Ohio;* ^*2*^*University of Michigan, Ann Arbor, Michigan;* ^*3*^*Texas Children’s Hospital, Houston, Texas;* ^*4*^*Medical College of Wisconsin, Milwaukee, Wisconsin;* ^*5*^*Golisano Children’s Hospital, Rochester, New York;* ^*6*^*Seattle Children’s Hospital, Seattle, Washington;* ^*7*^*Children’s Hospital and Medical Center, Omaha, Nebraska;* ^*8*^*Mayo Clinic, Rochester, Minnesota;* ^*9*^*University of Puerto Rico Hospital, San Juan, Puerto Rico;* ^*10*^*University of Iowa, Iowa City, Iowa*

**Background: **CArdio-Renal PEdiatric DIalysis Emergency Machine (Carpediem™, Medtronic, United States) is a dedicated infant continuous kidney replacement therapy (CKRT). The multidisciplinary collaborative, Improving Carpediem™ Outcomes in Neonates and Infants through collaboration (ICONIC), aims to understand best education and clinical practices. Nursing (RN) competency is critical for successful therapy. Often, the number of trained CKRT RNs exceeds the number of patients making it challenging for consistent exposure and skill acquisition. Training methods impact the RN’s ability to safely initiate, troubleshoot and resolve circuit issues, potentially influencing filter life and delay in care.

**Methods: **We designed and disseminated a survey to ICONIC sites that actively provide Carpediem™ as of August 1, 2022. Information collected related to intensive care units (ICUs) offered, care delivery models, staff roles, and education and training were uploaded into the Research Electronic Data Capture (REDCap) database.

**Results: **Ten of 22 sites reported ability to provide Carpediem™. Nine sites completed the survey. Carpediem™ is offered at all sites in the pediatric ICU with 5 sites additionally in the neonatal ICU. A collaborative nursing model was the most used (n=6), most commonly with dialysis and critical care (n=4). All, but two sites reported that bedside RNs had a role related to providing Carpediem™. Median ratio of Carpediem™ trained RNs for every unit bed was 1:1. Median ratio of Carpediem™ trained RNs to ICU total RNs was 1:4 (range 1:10 and 4:10). Nursing-to-patient ratios were predominantly 1:1, although 3 sites had at least one ICU with 2:1. Table 1 demonstrates initial nursing education requirements with 3 sites currently requiring annual competencies.

**Conclusions: **Standards for ideal nursing educational requirements and care delivery models are limited and vary amongst institutions. Understanding initial and ongoing educational requirements and how it influences the quality of delivered therapy and patient outcomes will help inform best practices related to CKRT in the future.

**Tables**

Table 1. Initial educational requirements to provide Carpediem™ Therapy

**Table:** 

**Type of Education**	**Bedside Nurse**		**CKRT Nurse**	
	# of Sites	Range of Hours required	# of Sites	Range of Hours required
**Didactic with Hands-on**	7	4-8	7	2-15
**Bedside Mentoring**	3	8-12	3	4-36
**Simulation**	1	4	2	2-34
**Online Modules**	0	N/A	1	2
**Exam**	3		7	

*Additional education not otherwise described- bedside just-in-time training and bedside champion resource support

**Keywords: **Education, Carpediem™, Neonatal CKRT

**Disclosures: ***Jolyn Morgan is a consultant for Medtronic*

*Shina Menon and Kat Gist (ICONIC Collaborative) are recipients of a Gerber Foundation Grant*

**References: **None

**Abstract 019**

**Ionized Magnesium Correlates with Total Magnesium in High-Risk Kidney Cohorts**

Denise C Hasson^1*^, Shruthi Mohan^1^, James E Rose^1^, Kyle A Merrill^1^, Charles Varnell^1^, Stuart L Goldstein^1^, Stefanie W Benoit^1^

^*1*^ *Cincinnati Children’s Hospital Medical Center, Cincinnati, OH*

**Background:** Serum magnesium (Mg) concentration abnormalities are common in critically ill children and kidney transplant (KTx) recipients, and abnormal values are associated with poor outcomes including mortality. Magnesium homeostasis is affected by kidney function, acid-base status, calcium-vitamin D alterations, and medications (eg, immunosuppressants, citrate). The active form, ionized Mg (iMg), is not measured, and studies conflict regarding total (tMg) and iMg correlation. We hypothesized iMg and tMg concentrations will be categorized differently (i.e., low, normal, high) in patients after KTx and on continuous kidney replacement therapy (CKRT) with citrate, but ionized calcium (iCa) would correlate with iMg.

**Methods:** We collected whole blood from patients in a single center for iMg and iCa measurement. Each CKRT pt could contribute multiple samples as our timepoints would reset with each circuit change. Total Mg and Ca were collected as standard of care. Demographic, lab, and outcome data were recorded from the electronic medical record. iMg and iCa were categorized using “normal ranges” of 0.44-0.65 mmol/L and 1.0-1.3 mmol/L, respectively, based on prior studies and clinical significance. Fisher’s Exact test and Pearson correlation studies were used for statistical analysis.

**Results:** In 9 KTx patients (n= 28 samples), iMg and tMg had similar categorization (p<0.001) and correlated well (R=0.811, p<0.001, Table 1A), but iCa did not differentiate normal from abnormal iMg concentrations (p=0.118). In 11 CKRT patients (n=70 samples), more time on CKRT resulted in more ionized hypomagnesemia despite 65 of 70 samples having normal tMgs: 17/30 (57%) of pre-CRRT, 15/19 (79%) of the 1-2 hour, and 15/16 (94%) of the 18-28 hour iMgs were low. On CKRT, there was category agreement (p=0.028, Table 1B) and moderate correlation between iMg and tMg (R=0.54, p<0.0001), but poor category agreement (p=0.50) and correlation between iMg and iCa (R=0.178, p=0.138).

**Conclusion:** iMg and tMg correlated in both groups, with greater category agreement in the KTx population; thus, it is likely that tMg represents active Mg in these patients. CKRT patients exposed to citrate had progressive ionized hypomagnesemia despite normal tMg and may benefit from supplementation. iCa should not be used as a surrogate for iMg concentrations.

**Keywords:** electrolyte disturbance, ionized magnesium, ionized calcium, continuous kidney replacement therapy

**Table 1 A, B.** Categorization of ionized magnesium (columns) and total magnesium (rows) in **A.** post-transplant (p<0.001) and **B.** CKRT samples.

**A.** iMg

**Table:** 

tMg		Low	Normal	High
	Low	8	2	0
	Normal	1	11	5
	High	0	0	1

**B.** iMg

**Table:** 

tMg		Low	Normal	High
	Low	3	0	0
	Normal	47	17	1
	High	0	1	1

**Disclosures:** SLG reports receiving personal fees from Baxter Healthcare, BioPorto Inc., CHF Solutions, Fresenius, MediBeacon, and Medtronic.

**Abstract 020**

**Utilization of urinary NGAL screening for acute kidney injury (AKI) in critically ill neonates exposed to nephrotoxic medications**

Christine Stoops^1*,4^, Hailey Gavigan^3^, Kelli Krallman^2^, Nekayla Anderson^1^, Russell Griffin^1^, Cara Slagle^2^, Scott House^4^, SL Goldstein^2^, DJ Askenazi^1,4^

^*1*^ *University of Alabama at Birmingham, Birmingham, AL, USA;* ^*2*^*Cincinnati Children’s Hospital, Cincinnati, OH, USA;* ^*3*^*Levine Children’s Hospital, Charlotte, NC, USA;* ^*4*^*Children’s of Alabama, Birmingham, AL, USA*

**Background:** Nephrotoxic medication (NTM) exposure is commonly associated with acute kidney injury (AKI) in the neonatal intensive care unit (NICU) [1]. A quality improvement (QI) initiative, Baby NINJA (Nephrotoxic Injury Negated by Just-in-time Action), has demonstrated significant reductions of NTM-AKI in the NICU [2]. The QI program systematically screens for AKI in those exposed to three or more NTM in a 24-hour period, or an intravenous aminoglycoside or intravenous vancomycin for ≥72 hours or ≥4 calendar days. Current protocol advises daily serum creatinine (SCr) levels. While the Baby NINJA program has demonstrated sustained results over multiple years, a venipuncture for daily SCr measurements is invasive and is associated with both personnel healthcare costs and increases in neonatal pain scale scores [3]. Urinary neutrophil gelatinase associated lipocalin (uNGAL) is a marker of renal tubular injury and has been associated with some NTM-AKI events [4,5]. We tested the hypothesis that uNGAL could reliably screen for AKI in Baby NINJA. If validated, uNGAL could decrease the need for daily venipuncture.

**Methods:** This two-center prospective study enrolled 174 NICU subjects, 148 who met criteria for the study from January 29, 2019, to September 18, 2020 (2.5-month enrollment paused due to the COVID pandemic). Daily urine samples were obtained for up seven days of qualifying NTM exposure plus two days after exposure ended or end of AKI, whichever occurred last. The primary outcome was AKI defined by the KDIGO SCr criteria. The maximum uNGAL was defined as the highest NGAL value during screening.

**Results:** Maximum urine NGAL thresholds of ≥400 ng/mL demonstrated strong negative predictive value (97.2%) for ruling out AKI with a ROC-AUC value of 0.72 and a positive likelihood risk of 2.76 (1.39-4.13) indicating that those above this uNGAL threshold are 2.76 times as likely to have AKI as those below threshold. Table 1 lists all predictive power tests for set thresholds of maximum uNGAL values.

**Conclusions:** We propose that uNGAL could replace some venipunctures needed for NTM-AKI screening in the Baby NINJA program. However, the ideal combination of SCr and uNGAL threshold requires further investigation in neonates, including gestational age stratification studies.

**Keywords:** acute kidney injury, nephrotoxic medication, neonate, infant

**Disclosures:** David Askenazi has consulting/grants from Baxter, Nuwellis, Medtronic, Bioporto, Seastar, Portero. He is the Chief Scientific Officer and Founder of Zorro-Flow Inc. Stuart Goldstein receives royalties from Vigilanz Corporation for licensing the NINJA application.

**Corresponding Author:** Christine, N, Stoops, https://www.ncbi.nlm.nih.gov/myncbi/christine.stoops.1/bibliography/public/

**References**

1. Salerno SN, et al. J Pediatr. 2021; 228: 213-219. DOI: 10.1016/j.jpeds.2020.08.035

2. Stoops C, et al. J Pediatr. 2019; 215:223-8.e6. DOI: 10.1016/j.jpeds.2019.08.046

3. Taksande AM, et al. Indian J Pediatr. 2005; 72, 751-753. DOI: 10.1007/BF02734146

4. Cassidy H, et al. Biochim Biophys Acta Mol Basis Dis. 2019;1865(12):165532. DOI: 10.1016/j.bbadis.2019.165532

5. Goldstein SL. Pediatr Nephrol. 2021;36(7):1915-21. DOI: 10.1007/s00467-020-04898-5

**Table 1**. Predictive power of maximum urinary neutrophil gelatinase-associated lipocalin (NGAL) values for acute kidney injury (AKI)

**Table:** 

**uNGAL**	**Sensitivity**	**Specificity**	**PPV**	**NPV**	**LR+**	**LR-**	**AUC**
≥150 ng/mL	70.0% (41.6-98.4%)	53.6% (45.3-61.9%)	9.9% (2.9-16.8%)	96.1% (91.8-100%)	1.51 (0.84-2.18)	0.56 (0.02-1.10)	0.62 (0.46-0.77)
≥250 ng/mL	70.0% (41.6-98.4%)	65.9% (58.0-73.9%)	13.0% (4.0-21.9%)	96.8% (93.3-100%)	2.06 (1.09-3.02)	0.46 (0.02-0.89)	0.68 (0.52-0.83)
≥300 ng/mL	70.0% (41.6-98.4%)	69.6% (61.9-77.2%)	14.3% (4.5-24.1%)	97.0% (93.6-100%)	2.30 (1.20-3.40)	0.43 (0.02-0.84)	0.70 (0.54-0.85)
≥400 ng/mL	70.0% (41.6-98.4%)	74.6% (67.4-81.9%)	16.7% (5.4-27.9%)	97.2% (94.0-100%)	2.76 (1.39-4.13)	0.40 (0.02-0.78)	0.72 (0.57-0.88)
≥1000 ng/mL	50.0% (19.0-81.0%)	85.5% (79.6-91.4%)	20.0% (4.3-35.7%)	95.9% (79.6-91.4%)	3.45 (0.90-6.00)	0.59 (0.22-0.95)	0.68 (0.51-0.84)
≥2000 ng/mL	40.0% (9.6-70.4%)	91.3% (86.6-96.0%)	25.0% (3.8-46.2%)	95.5% (91.9-99.0%)	4.60 (0.31-8.89)	0.66 (0.32-0.99)	0.66 (0.49-0.82)

PPV = positive predictive value; NPV = negative predictive value; LR = likelihood ratio; AUC = area under the curve

**Abstract 021**

**Neonatal Extracorporeal Life Support: Associations between Continuous Renal Replacement Therapy, Thrombocytopenia, and Outcomes**

Lauren R. Walker^1*^, MD, W. Michael Southgate^1^, MD, David T. Selewski^1^, MD, MSCR, Laura Hollinger^1^, MD, Jeffrey E. Korte^1^, PhD, Mathew Gregoski^1^, PhD, Heidi J. Steflik^1^, MD, MSCR

^*1*^ *Medical University of South Carolina, Charleston, South Carolina, USA*

**Background:** The incidence of thrombocytopenia in neonates receiving extracorporeal life support (ECLS) with and without concurrent continuous renal replacement therapy (CRRT) and associated complications have not been well described. We aimed to evaluate associations between CRRT, severe thrombocytopenia (platelets <50,000), and outcomes in neonates receiving ECLS and identify predictors of severe thrombocytopenia.

**Methods:** We conducted a single-center chart review of neonates who received ECLS 07/01/14 - 03/01/20. Provider discretion dictated CRRT use in the pediatric and cardiac intensive care units (PICU and PCICU, respectively); all patients in the neonatal ICU (NICU) received CRRT. We evaluated associations between CRRT, severe thrombocytopenia, and outcomes (ECLS duration, length of stay (LOS), and mortality) using Fisher’s exact, Chi-squared, Student’s t, and independent-samples median tests, as appropriate. Exploratory classification and regression tree (CART) analysis was performed to identify optimal predictors of severe thrombocytopenia including characteristics that differed between those with and without severe thrombocytopenia (CRRT, birthweight, ICU location).

**Results:** Fifty-two neonates received ECLS; 35 (67%) received concurrent CRRT. Severe thrombocytopenia occurred in 27 (52%) neonates and in 21 (50%) CRRT-receivers. Underlying diagnosis (+CRRT 37% respiratory, -CRRT 94% cardiac; p<0.01), ECLS mode (+CRRT 66% venoarterial ECLS, -CRRT 100% venoarterial ECLS; p=0.01), care unit (+CRRT 83% NICU, -CRRT 100% PCICU; p<0.01) and moderate/severe hemolysis (+CRRT 23%, -CRRT 0%; p=0.04) differed between those who did and did not receive CRRT. CRRT-receivers experienced shorter hospital stays (+CRRT: median 33 IQR [22-49] days, -CRRT: 50 [35-78] days; p<0.05) than CRRT non-receivers, but ECLS duration, length of ICU stay, and mortality did not differ between groups. CRRT receipt was associated with severe thrombocytopenia (p<0.01). When hospital LOS was examined by CRRT receipt and severe thrombocytopenia, no differences were detected. CART analysis yielded a learning AUC of 0.87 and a test AUC of 0.72 (Figure).

**Conclusions:** In our cohort, CRRT use during ECLS was associated with severe thrombocytopenia, and patients who received ECLS with CRRT experienced shorter hospital stays than those who did not receive CRRT. Exploratory CART analysis suggests CRRT use, birthweight, and ICU location are all predictors of interest for severe thrombocytopenia and worthy of further investigations in larger studies.

**Keywords:** extracorporeal membrane oxygenation, continuous kidney support therapy, thrombocytopenia

**Disclosures:** HS receives grant funding from Baxter. The other authors have no disclosures or conflicts of interest to report.

**Figure. Exploratory Classification and Regression Tree (CART) Analysis Evaluating Predictors of Severe Thrombocytopenia in Neonates Receiving ECLS** 
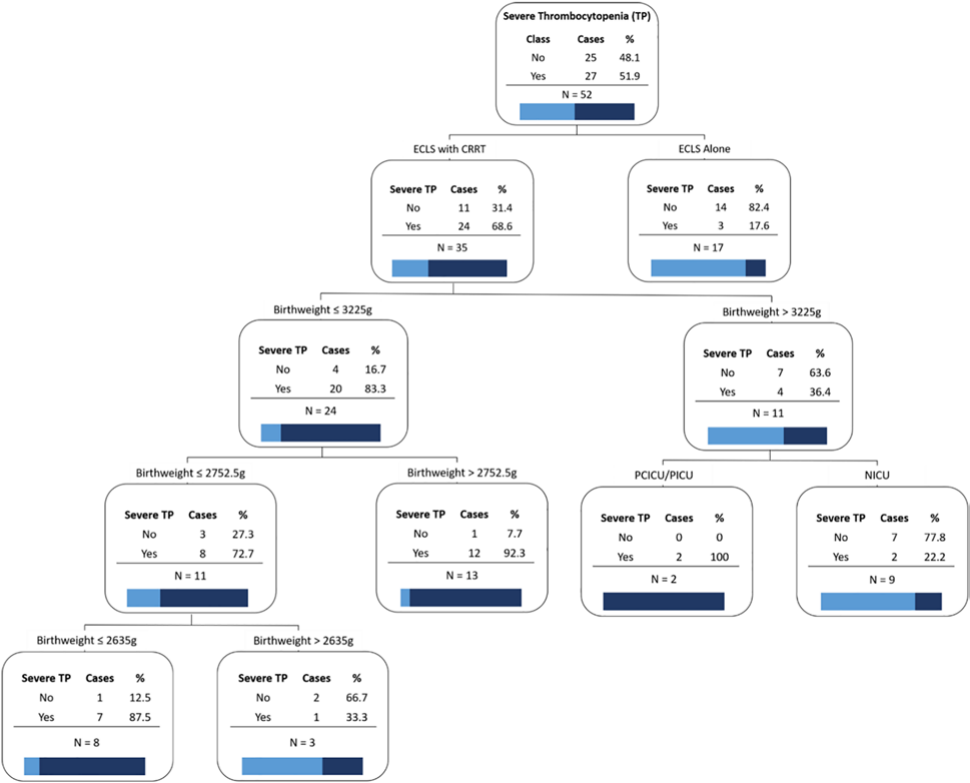


*TP,* thrombocytopenia; *ECLS,* extracorporeal life support; *CRRT,* continuous renal replacement therapy; *PCICU, pediatric cardiology* intensive care unit; *PICU, pediatric* intensive care unit; *NICU,* neonatal intensive care unit; g, grams

**Abstract 022**

**Warm Ischemia Time during Pediatric Kidney Transplant does not correlate with Urine Acute Kidney Injury Biomarkers**

Kyle A. Merrill^1*^ MD, Cassie Kirby^2^, Lakhmir Chawla^3^, MD, David K. Hooper^2^ MD, MS, Stuart L. Goldstein^2^, MD

^*1*^ *University of Iowa Stead Family Children’s Hospital, Iowa City, IA, USA;* ^*2*^*Cincinnati Children's Hospital Medical Center, Cincinnati, OH, USA;* ^*3*^*VA San Diego Healthcare System, San Diego, CA, USA*

**Background:** Ischemia-reperfusion injury (IRI) is a mechanism of acute kidney injury (AKI). Like cardiopulmonary bypass, kidney transplantation is a form of controlled IRI resulting in acute tubular necrosis and possibly delayed graft function (DGF). Therefore, we sought to investigate kidney transplantation in pediatric patients as a clinical model of AKI, focusing on warm ischemia time (WIT) and novel AKI urine biomarkers.

**Methods:** Prospective study of patients aged 3 months to 26 years who received a kidney transplant from 7/2020 and 11/2021. Urine was collected daily for 7 days post-transplant. Biomarkers tested were neutrophil gelatinase-associated lipocalin (NGAL), kidney injury molecule 1 (KIM-1), interleukin 18 (IL-18), and hepcidin. DGF was defined as receiving kidney replacement therapy within 7 days post-transplant. Wilcoxon rank sum test was used to compare cohorts. Linear regression was used to compare biomarker concentrations to WIT on post-operative days 0-2. The optimal cutoff for NGAL to predict lack of DGF was determined using negative predictive value (NPV) and a Youden’s index.

**Results:** 4/30 patients developed DGF with a longer WIT of 55 minutes (IQR 52-57) vs. 38 minutes (IQR 32-45). Thus, we compared biomarker concentrations in patients with WIT <45 vs. ≥45 minutes. NGAL, KIM-1 and Hepcidin did not differ between cohorts. IL-18 was higher on day 1 in the ≥45-minute WIT cohort. A positive linear relationship was noted between WIT and NGAL on day 2 and a negative relationship with hepcidin on day 0. The optimal Youden’s index was 0.6 for an NGAL cutoff of 150 ng/mL on either day 0 or 1 post-transplant with a NPV of 95.7% (95% CI 80-99%).

**Conclusions:** We found no consistent correlation between WIT and biomarkers suggesting that AKI may be more complex than simply IRI. NGAL <150 ng/mL on post-operative day 0 or 1 correlated to a high NPV for development of DGF.

**Keywords:** Pediatric, Kidney Transplant, Ischemia-Reperfusion Injury, Biomarkers

**Disclosures:** SLG has relevant disclosures that were submitted to the conference. The remainder of authors have no relevant financial disclosures or competing interests.

**Corresponding Author:** Kyle A Merrill, https://orcid.org/0000-0001-8238-7939

**Table 1:** Analysis of urine biomarker levels post kidney transplant related to warm ischemia time.

**Table:** 

	Warm Time < 45 minutes N=19	Warm Time > 45 minutes N=11	p value	Linear Regression		
				Estimate (95% CI)	p value	R^2^
NGAL (ng/mL)						
POD 0*	92.9 (26.8 - 283)	N=10 300 (93.7 -430)	0.18	19.63 (-46.4 - 85.7)	0.547	0.014
POD 1*	N=18 37.8 (22.7 - 96.6)	58.5 (31.1 - 258)	0.41	31.65 (-2.2 - 65.5)	0.0657	0.12
POD 2*	35.7 (17.2 - 109)	135 (51.8 - 214)	0.085	33.9 (1.5 - 66.4)	**0.041**	0.14
KIM-1 (ng/mL)						
POD 0*	0.84 (0.26 - 2.0)	N=10 0.52 (0.26 - 1.0)	0.40	-0.02 (-0.11 - 0.07)	0.65	0.008
POD 1*	N=18 3.1 (0.9 - 8.1)	3.9 (2.4 - 9.0)	0.28	0.10 (-0.04 - 0.25)	0.17	0.07
POD 2*	2.66 (0.75 - 5.3)	3.5 (2.0 - 8.2)	0.29	0.09 (-0.05 - 0.23)	0.21	0.055
IL-18 (pg/mL)						
POD 0*	15.6 (15.6 - 72.1)	N=10 15.6 (15.6 - 34.1)	0.70	-0.93 (-2.8 - 0.92)	0.31	0.038
POD 1*	15.6 (15.6 - 20.1)	34 (24.5 - 79.2)	**0.016**	-0.52 (-7.7 - 6.7)	0.89	0.0008
POD 2*	15.6 (15.6 - 20.3)	20.5 (15.6 - 35.8)	0.13	0.72 (-0.26 - 1.7)	0.15	0.07
Hepcidin (ng/mL)						
POD 0*	457 (302 - 553)	N=10 249 (51.4 - 453)	0.07	-9.8 (-18.7 - -0.85)	**0.033**	0.16
POD 1*	241 (50.9 - 427)	130 (104 - 243)	0.87	-0.6 (-10.6 - 9.35)	0.90	0.0006
POD 2*	164 (56.6 - 282)	141 (108 - 259)	0.73	4.99 (-4.46 - 14.4)	0.29	0.04

*Median(IQR), POD – post-operative day

**Abstract 023**

**Patient level factors increase risk of acute kidney disease in hospitalized children with acute kidney injury**

Mital Patel^1*^, Christoph Hornik^1^, Clarissa Diamantidis^1^, David Selewski^2^, Rasheed Gbadegesin^1^

^*1*^ *Duke University, Durham, NC, USA;* ^*2*^*Medical University of South Carolina, Charleston, SC, USA*

**Background:** Studies of AKI in adults have shown that persistent kidney dysfunction ≥7-90 days, termed acute kidney disease (AKD), increases chronic kidney disease (CKD) and mortality risk. However, in the pediatric population, little is known about the transition of AKI to AKD. The aim of this study is to evaluate the risk factors for progression of AKI to AKD in hospitalized children and quantify the associated incidence of CKD.

**Methods:** We quantified AKD risk using a retrospective cohort of 528 children age ≤ 18 admitted with AKI to Pediatric Intensive care units and general wards at a tertiary care children’s hospital between 2015-2019. AKI and AKD were defined using Kidney Disease Improving Global Outcomes criteria. CKD was defined as new estimated glomerular filtration rate of < 60 ml/min/1.73m2 persisting for >3 months at >90 days after AKI. Exclusion criteria included insufficient creatinine values to evaluate for AKD, chronic dialysis, or previous kidney transplant. We used a stepwise selection (forward inclusion p <0.05; backward elimination p >0.1) of biologically plausible predictor variables to build a parsimonious logistic regression model predicting probability of AKD in children with AKI. We conducted standard assumption diagnostics and report model parameters in odds ratios (OR) with 95% confidence intervals (CI).

**Results:** In this cohort, 297 (56.3%) hospitalized AKI survivors developed AKD. A multivariable logistic regression model identified preexisting conditions, iatrogenic factors, AKI stage, and duration of kidney injury as risk factors for AKD (Table 1). Among children with AKD, 23.6% developed CKD compared to 13.4% in the group without AKD (OR 2.9, 95% CI 1.75-4.77).

**Conclusions:** Our data shows that AKD is common among hospitalized children with AKI and multiple modifiable patient level risk factors are associated with AKD. In addition, AKI survivors with AKD are at higher risk of developing CKD than those without AKD.

**Keywords:** Acute kidney injury, acute kidney disease

**Disclosures:** None

**Table 1: Multivariable logistic regression model for development of AKD in children with AKI**

**Table:** 

**Covariate**	**OR**	**95% CI**	**P-value**
Age at diagnosis (months)	1.01	1.00-1.01	0.004
Hospital location at AKI diagnosis- PICU relative to general ward	1.71	0.89-3.28	0.109
Hospital location at AKI diagnosis- PCICU relative to general ward	3.53	1.60-7.79	0.002
Hospital location at AKI diagnosis- NICU relative to general ward	3.12	1.22-7.97	0.018
Prematurity ≤ 36 weeks	2.29	1.16-4.49	0.016
Malignancy	2.60	1.17-5.78	0.019
Bone marrow transplant	8.18	3.26-20.51	<0.001
Previous AKI before study period	3.01	1.41-6.44	0.004
Mechanical ventilation at AKI diagnosis	2.32	1.36-3.97	0.002
Pressor support at AKI diagnosis	0.49	0.28-0.87	0.014
Duration of AKI (days)	1.25	1.12-1.39	<0.001
AKI stage 2	1.96	1.10-3.50	0.023
AKI stage 3	3.48	1.86-6.51	<0.001
Renal replacement therapy during AKI period	3.52	1.60-7.77	0.002

**Abstract 024**

**In-vivo Assessment of a Manual Single Lumen Alternating Micro-Batch Hemodiafiltration (mSLAMB) System**

Sabrina Lanker^1^, Christopher J. Pino^1^, H. David Humes^1^, Lakhmir Chawla^2^, Kimberly A. Johnston^1^

^*1*^ *Innovative BioTherapies Inc, Ann Arbor, MI, USA;* ^*2*^*Veterans Affairs Medical Center, San Diego, USA*

**Background:** The manual single lumen alternating micro-batch hemodiafiltration (mSLAMB) system is a closed-loop dialysis system designed to provide kidney support in emergency situations (e.g., fluid overload, hyperkalemia, acidemia). If done repeatedly in small batches at high flow rates, this system can achieve clearance levels comparable to traditional renal replacement therapy (RRT)^1^. The purpose of this system is to help patients with acute kidney injury (AKI) in austere environments at a low cost (<$25). The manual circuit requires no electricity or batteries and uses a more modest vascular access than traditional modalities.

**Methods:** Nephrectomized pigs (n=4, 14-16kg) were treated with SLAMB manual dialysis to assess removal of uremic toxins and an exogenous florescent tracer. Various RRT modalities were attempted including hemofiltration with and without predilution as well as hemodiafiltration with variations on dialysate flow based on adjusting height of circuit elements. Eight to sixteen cycles using batch volumes of 100-150ml were completed on each animal. Samples were taken periodically to analyze clearance of toxins from blood as well as quantify cleared molecules in dialysate.

**Results:** All analytes were shown to be removed across the dialyzer with each micro batch evaluated. Potassium clearances of 4 to 9 ml/min were achieved. The exogenous florescent tracer was shown to be cleared from the systemic blood with subsequent cycles. High dialysate flows yielded higher clearances as well as passive fluid removal. With this configuration we removed up to 250mL of fluid and 15 mmol of potassium with eight cycles, approximately an hour of treatment.

**Conclusion:** Electrolyte derangements and volume overload remain life threating emergencies in low resource settings. With the mSLAMB system, micro-batch processing was successful at removing a significant fluid volume and also effective at clearing uremic toxins and the exogenous florescent tracer. These studies demonstrate proof of concept for efficacy of mSLAMB in treatment of AKI by providing removal of potassium as well as excess fluid, such that additional stabilizing therapies, such as isotonic bicarbonate solutions can safely be administered.

**Keywords:** Single lumen alternating micro-batch (SLAMB), renal replacement therapy (RRT), acute kidney injury (AKI), dialysis

**Disclosures:** KAJ, CJP, SLL, HDH are employees of Innovative BioTherapies (IBT). HDH owns IBT. LC is a shareholder of Stavro Medical.

**Corresponding Author:** KAJ, email: kjohnston@innbio.com

**References:** Single Lumen Alternating Micro-Batch Hemodiafiltration (SLAMB-HDF): A Device for Minimally Invasive Renal Replacement Therapy. Lakhmir S. Chawla. Kidney360 Sep 2020, 1 (9) 969-973; DOI: 10.34067/KID.0001462020 
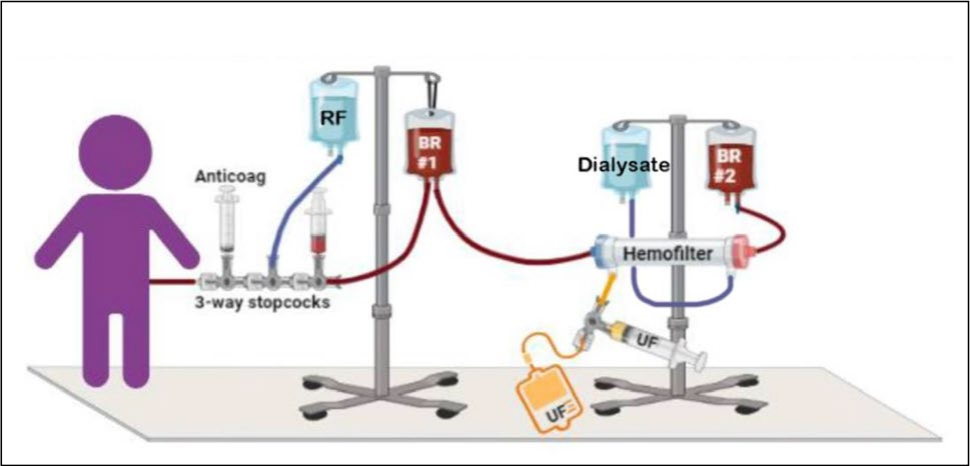


**Figure 1**. Illustration of the Manual Single Lumen Alternating Micro-Batch Hemodiafiltration (mSLAMB) system. A batch of blood is removed from the patient manually using a syringe then moved through the closed loop system using gravity flow by adjusting the height of the reservoir bags. Blood is returned by performing maneuvers in reverse order. Ultrafiltration can be performed manually or can be passively achieved during dialysate flow. BR=blood reservoir, RF=replacement fluid, UF=ultrafiltrate

**Abstract 025**

**Manual Single-Lumen Alternating Micro Batch (mSLAMB) dialysis achieves safe and reliable clearance via diffusion**

Giovanni Ceschia^1*^, Apaara K Chawla^2^, Jolyn Morgan^3^, James Rose^3^ and Denise C Hasson^3^

^*1*^ *University of Padua, Padua, Italy;* ^*2*^*Francis Parker High School, San Diego, CA;* ^*3*^*Cincinnati Children’s Hospital Medical Center, Cincinnati, OH*

**Background:** Acute kidney injury (AKI) remains a cause of preventable deaths in low resource settings due to prohibitive costs and lack of basic dialysis access. A single lumen alternating micro-batch (SLAMB) dialysis technique performs kidney replacement therapy (KRT) using single lumen access, a low-cost set of bags and tubing, premade fluids, and a dialysis filter. We have previously shown that a manual variation of SLAMB (mSLAMB) works without electricity, a battery, or a pump. We hypothesized that mSLAMB can perform diffusive clearance efficiently, and a simple, reliable, and safe protocol can bring dialysis to a population who previously did not have it.

**Methods:** Bags of expired packed red blood cells mixed with crystalloid solution were made to desired sizes (0.5-2L) and hematocrits (low vs normal) to simulate infant, toddler, and pediatric blood volumes. Bags were spiked with 1-5 grams of urea and anticoagulated with heparin. A Static diffusion Technique (with 5 second flushes of dialysis fluid before each filter pass) and a Dynamic diffusion Technique (with varying volumes of dialysis fluid running through the filter during a pass) were trialed and urea and potassium clearance were measured. The difference between the 200mL batch volume and volume returned to the blood bag per cycle represented passive ultrafiltration.

**Results:** 5 cycles routinely achieved urea reduction rates (URR) between 17-67% and potassium clearance of 18-60%. The main factor causing these wide ranges in rates was the proportion of batch volume dialyzed to patient volume: 0.5L bags achieved 40-67% URR, 0.8-1.45L bags had 26-56% URR, and 2L bags had 17-31% URR (Figure 1). The Dynamic Technique appears to have had a slightly better URR compared to the Static Technique but required more dialysis fluid and time to change/make effluent and dialysis fluid bags, respectively. Passive ultrafiltration occurred in both Static and Dynamic techniques and the filtered volume depended on the height of the bags from the filter.

**Conclusion:** mSLAMB dialysis performed diffusive clearance and passive ultrafiltration with few resources and manpower.

**Keywords:** Acute kidney injury, kidney replacement therapy, austere medical environment, low resource dialysis.

**Disclosures:** Stuart L Goldstein reports receiving personal fees from Baxter Healthcare, BioPorto Inc., CHF Solutions, Fresenius, MediBeacon, and Medtronic. Jolyn Morgan is a consultant for Medtronic.

**Corresponding Author:** Giovanni Ceschia, Department of Women’s and Children’s Health, University of Padua, Via Giustiniani, 3, Padua, 35128, Italy, +39 3297191464, giovanni.ceschia@aopd.veneto.it 
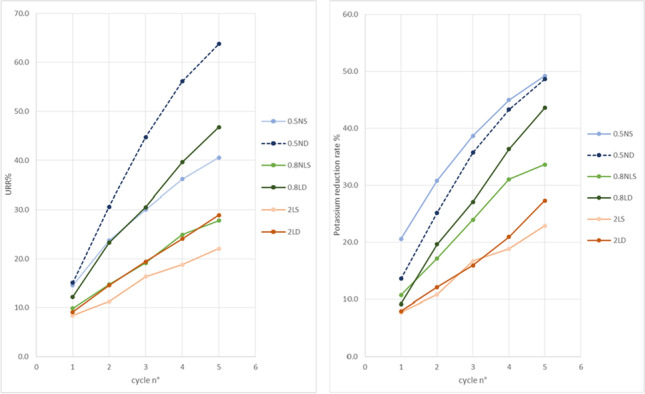


***Figure 1A and 1B.*** *URR and K clearance by blood bag size and technique (means).*

**Abstract 026**

**Assessment of fluid balance after neonatal cardiac surgery: intake/output- vs weight-based methods**

Tara M Neumayr^1*^, MD, Jeffrey A Alten^2^, MD, David K Bailly^3^, DO, Priya N Bhat^4^, MD, MS, Katie L Brandewie^2^, MD, J. Wesley Diddle^5^, MD, Muhammad Ghbeis^6^, MD, Catherine D Krawczeski^7^, MD, Kenneth E Mah^8^, MD, MS, Tia T Raymond^9^, MD, Garrett Reichle^10^, MS, Huaiyu Zang^2^, PhD, David T Selewski^11^, MD, NEPHRON Investigators^**^

^*1*^ *Washington University School of Medicine, St. Louis, MO, USA;* ^*2*^*University of Cincinnati College of Medicine, Cincinnati Children’s Hospital Medical Center, Cincinnati, OH, USA;* ^*3*^*University of Utah, Salt Lake City, UT, USA;* ^*4*^*Baylor College of Medicine, Texas Children’s Hospital, Houston, TX, USA;* ^*5*^*Children’s National Hospital, Washington, DC, USA;* ^*6*^*Harvard Medical School, Boston Children’s Hospital, Boston, MA, USA;* ^*7*^*The Ohio State University College of Medicine, Nationwide Children’s Hospital, Columbus, OH, USA;* ^*8*^*Stanford University School of Medicine, Palo Alto, CA, USA;* ^*9*^*Medical City Children’s Hospital, Dallas, TX, USA;* ^*10*^*University of Michigan, Ann Arbor, MI, USA;* ^*11*^Medical University of South Carolina, Charleston, SC, USA.^**^NEPHRON Investigators (Contributing Authors): *The following individuals served as collaborators and site investigators for the NEPHRON study and are collaborators on this manuscript and should be indexed in PubMed as collaborators on this manuscript:*

Parthak Prodhan^a^, MD, Xiomara Garcia^a^, MD, Shannon Ramer^a^, BSN, RNC, Mindy Albertson^a^, RN, Michael Gaies^b^, MD, MPH, MS, David S. Cooper^b^, MD, MPH, Zahidee Rodriquez^b^, MD, Mary Lukacs^b^, Dominic Zanaboni^c^, MD, Joan Sanchez de Toledo^d^, MD, PhD, Yuliya A. Domnina^d^, MD, Lucas Saenz^d^, MD, Tracy Baust^d^, BA, Jane Kluck^e^, RN, BSN, Linda Duncan^e^, RN, BSN, Joshua D. Koch^f^, MD, Joshua Freytag^g^, Amanda Sammons^g^, Hideat Abraha^g^, John Butcher^g^, Jun Sasaki^h^, MD, Rebecca A. Bertrandt^i^, MD, Jason R. Buckley^j^, MD, Luke Schroeder^j^, MD, Aanish Raees^k^, MBBS, Lisa J. Sosa^l^, ARNP, Natasha S. Afonso^m^, MD, MPH, Erika R. O’Neal^m^, MD, Javier J. Lasa^m^, MD, Patrick A. Phillips^n^, Amy Ardisana^o^, Kim Gonzalez^o^, Tammy Doman^o^, Suzanne Viers^o^, Wenying Zhang^p^, MS, Kristal M. Hock^q^, MSN, RN, CNL, Santiago Borasino^q^, MD, MPH, and Joshua J. Blinder^r^, MD

^*a*^ *Arkansas Children’s Hospital, University of Arkansas for Medical Sciences, Little Rock, Arkansas;* ^*b*^*Division of Pediatric Cardiology, The Heart Institute, Department of Pediatrics, Cincinnati Children’s Hospital Medical Center, University of Cincinnati College of Medicine, Cincinnati, Ohio;* ^*c*^*Division of Cardiac Critical Care Medicine, Department of Anesthesia/Critical Care, Children’s Hospital of Philadelphia, University of Pennsylvania Perelman School of Medicine, Philadelphia, Pennsylvania;* ^*d*^*Department of Critical Care Medicine and Pediatrics, UMPC Children’s Hospital of Pittsburg, University of Pittsburg School of Medicine, Pittsburg, Pennsylvania;* ^*e*^*Children’s Hospital of Wisconsin, Milwaukee, Wisconsin;* ^*f*^*Division of Critical Care Medicine, Department of Pediatrics, Children’s Medical Center of Dallas, University of Texas Southwestern Medical School, Dallas, Texas;* ^*g*^*Cincinnati Children’s Hospital Medical Center, Cincinnati, Ohio;* ^*h*^*Division of Cardiac Critical Care Medicine, Nicklaus Children’s Hospital, Miami, Florida;* ^*i*^*Division of Critical Care, Department of Pediatrics, Children’s Wisconsin, Medical College of Wisconsin, Milwaukee, Wisconsin;* ^*j*^*Medical University of South Carolina Children’s Hospital, Charleston, South Carolina;* ^*k*^*Monroe Carell Jr. Children’s Hospital at Vanderbilt, Vanderbilt, Tennessee;* ^*l*^*Nicklaus Children’s Hospital, Miami, Florida;* ^*m*^*Pediatric Critical Care, Texas Children’s Hospital, Baylor College of Medicine, Houston, Texas;* ^*n*^*Children’s of Alabama, University of Alabama at Birmingham, Birmingham, Alabama;* ^*o*^*CS Mott Children’s Hospital, University of Michigan, Ann Arbor, Michigan;* ^*p*^*Center for Health Outcomes and Policy, University of Michigan, Ann Arbor, Michigan; and* ^*q*^*Section of Cardiac Critical Care Medicine, Department of Pediatric Cardiology, University of Alabama at Birmingham, Birmingham, Alabama.* ^*r*^*Lucile Packard Children’s Hospital Stanford, Palo Alto, California*

**Background:** Excessive fluid accumulation associates with poor outcomes after neonatal cardiac surgery, but consensus does not exist as to the most clinically relevant method of measuring fluid balance (FB). While weight-based FB (FB-W) is standard in neonatal intensive care units, weighing infants after cardiac surgery may be challenging. We aimed to identify patient characteristics associated with obtaining weights and to understand how intake/output-based FB (FB-IO) and FB-W compare in the early postoperative period in this population.

**Methods:** Observational retrospective study of 2235 neonates undergoing cardiac surgery from 22 hospitals comprising the Neonatal and Pediatric Heart and Renal Outcomes Network (NEPHRON) database.

**Results**: Of the 2235 patients, 45% (n = 998) were weighed on postoperative day (POD) 2, varying from 2 – 98% among centers. In multivariable analysis, the odds of being weighed on POD 2 were lower for Society of Thoracic Surgeons-European Association for Cardio-Thoracic Surgery Congenital Heart Surgery (STAT) categories 4 and 5 (OR 0.72; 95% CI 0.53-0.98), cardiopulmonary bypass (0.59; 0.42-0.83), delayed sternal closure (0.27; 0.19-0.38), prophylactic peritoneal dialysis use (0.58; 0.34-0.99), and mechanical ventilation on POD 2 (0.23; 0.16-0.33). Correlation between FB-IO and FB-W was weak for every POD 1 – 6 and within the entire cohort (correlation coefficient 0.15; 95% CI 0.12-0.17). FB-W measured higher than paired FB-IO (mean bias 12.5%; 95% CI 11.6%-13.4%) with wide 95% limits of agreement (-15.4%-40.4%).

**Conclusions:** Weighing neonates early after cardiac surgery is uncommon, with significant variation in practice among centers. Patients with increased severity of illness are less likely to be weighed. FB-W and FB-IO have weak correlation. Comparison of studies using different measures of cumulative FB must be approached with caution. Efforts to increase proportion of neonates weighed in the early postoperative period may be warranted, and further study is needed to determine which cumulative FB metric most associates with adverse outcomes.

**Key words:** fluid overload, cardiac surgery, neonate, weight

**Disclosures:**

Financial Support: Castin’ ‘N Catchin’ Charity Organization; Cincinnati Children’s Hospital Medical Center Heart Institute Research Core

Financial disclosures/conflicts of interest: None

**Fluid balance on POD2 by FB-IO and FB-W in patients weighed on both POD1 and POD2.** 
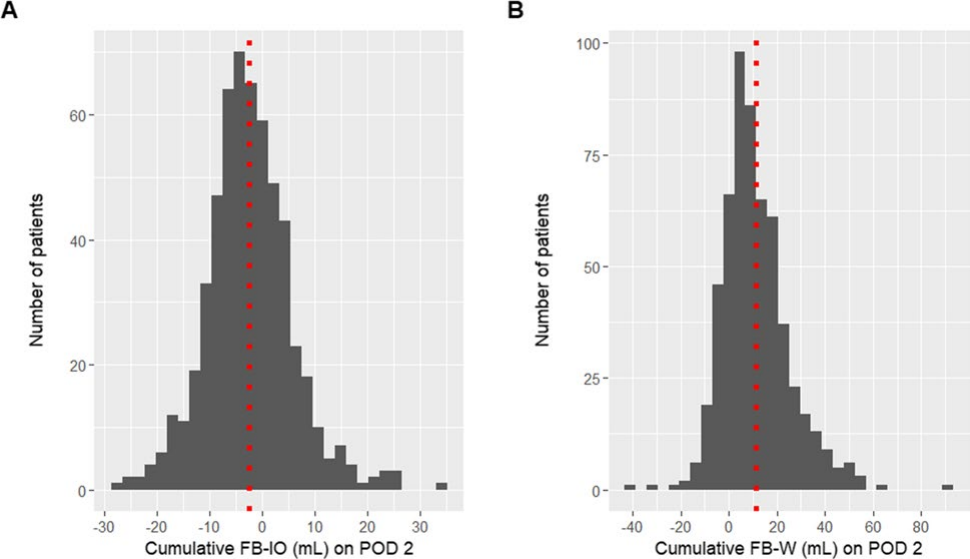


Mean fluid balance on POD2 for both FB-IO and FB-W is denoted by the dotted red line. Fluid balance assessed by the FB-IO method showed a modestly negative fluid balance on POD2 for the cohort, while paired assessments with the FB-W method demonstrated overall positive fluid balance at the same time point.

**Abstract 027**

**Costs associated with acute kidney injury in the neonatal intensive care unit: analysis of data from the pediatric health information system (PHIS) database**

Heidi J. Steflik^1*^, MD, MSCR, Daniel L. Brinton^1^, PhD, Corinne Corrigan^1^, PharmD, David T. Selewski^1^, MD, MSCR

^*1*^ *Medical University of South Carolina, Charleston, SC, USA*

**Background:** Between 30-70% of neonates in the neonatal intensive care unit (NICU) develop acute kidney injury (AKI) [1]. Despite this high incidence, the economic burden of AKI remains understudied.

We aimed to compare estimated costs of hospitalization between neonates in the NICU who did and did not develop AKI and identify predictors of AKI-associated costs. We hypothesized neonates who developed AKI would amass significantly more costs than those who did not, and these increased costs would be driven by premature birth, length of hospitalization (LOH), and renal replacement therapy (RRT) receipt.

**Methods:** Using data from the Children’s Hospital Association’s (CHA) Pediatric Health Information System (PHIS) database, we conducted a retrospective cohort study of neonates in the NICU between 01/01/2015-12/31/2021.

Baseline and demographic data were compared between those who did and did not develop AKI using Wilcoxon-Mann Whitney and Chi-squared tests, as appropriate. The adjusted, marginal total cost of hospitalization between those who did and did not develop AKI was estimated using a gamma-distributed log-transformed link function generalized linear model. Costs were inflation-adjusted to March 2022 US dollars using the Consumer Price Index The final model was adjusted for factors that might bias cost comparisons including race, ethnicity, gestational age (GA), Feudtner pediatric complex chronic conditions classification (CCC) status [2], and RRT.

**Results:** Data from 304,725 neonates, 8,774 (3%) with AKI and 295,951 (97%) without AKI, were included. Significant differences were found in every demographic and baseline characteristic between those who did and did not develop AKI (Table).

Significant predictors of costs (all p<0.01) included race, ethnicity, GA, Feudtner CCCs, and RRT. Neonates with AKI had, on average, $41,699 greater costs (95% CI: $39,923-43,513) than those without AKI after adjusting for these predictors (AKI: $97,542, 95% CI $95,577-99,547, No AKI: $55,843, 95% CI: $55,654-56,034).

**Conclusions:** In this analysis of the economic burden of AKI in the NICU, AKI is independently associated with increased hospital costs. Key drivers of costs of hospitalization among those who developed AKI included (in order of importance): LOH, Feudtner cardiovascular disease, GA, and RRT. Knowledge of these drivers can help in identifying high-value practices for cost mitigation strategies.

**Keywords:** costs, acute kidney injury, economic burden, cost mitigation

**Disclosures:** HJS receives grant funding from Baxter. The other authors declared no competing interests.

**Corresponding author:** Heidi J. Steflik, https://orcid.org/0000-0003-2168-4926

**References**

Jetton JG, et al. *Lancet Child Adolesc Health* 2017 Nov;1(3):184-194. http://doi.org/10.1016/S2352-4642(17)30069-X.

Feudtner et al. *BMC Pediatrics* 2014, 14:199. http://doi.org/10.1186/1471-2431-14-199.

**Table.** Demographic and Baseline Characteristics of Neonates with and without AKI

**Table:** 

**Demographics and Characteristic**		**Acute Kidney Injury**		***p*** **value**
		**No** **(n=295,951)**	**Yes** **(n=8774)**	
Sex	Male	159,097 (53.8)	5,257 (59.9)	<0.0001
	Female	136,620 (46.1)	3,505 (40.0)	
Race	Asian/Pacific Islander/ American Indian	13,879 (4.7)	383 (4.4)	<0.0001
	Black	52,046 (17.6)	1,870 (21.3)	
	White	160,661 (54.3)	4,657 (53.1)	
	Other	37,236 (12.6)	1,176 (13.4)	
	Unknown	32,129 (10.9)	688 (7.8)	
Ethnicity	Hispanic or Latino	45,503 (15.4)	1,838 (20.9)	<0.0001
	Not Hispanic or Latino	217,324 (73.4)	6,315 (72.0)	
	Unknown	33,124 (11.2)	621 (7.1)	
Birthweight	<500 grams	1,000 (0.3)	140 (1.6)	<0.0001
	>500 - <1,000 grams	13,403 (4.5)	1,894 (21.6)	
	>1,000 - <1,500 grams	17,893 (6.0)	566 (6.5)	
	>1,500 - <2,500 grams	67,768 (22.9)	1,238 (14.1)	
	>2,500 grams	161,976 (54.7)	3,955 (45.1)	
Gestational Age	22-26 weeks	9,991 (3.4)	1,682 (19.2)	<0.0001
	27-30 weeks	17,278 (5.8)	669 (7.6)	
	31-34 weeks	45,878 (15.5)	764 (8.7)	
	35-37 weeks	60,955 (20.6)	1,433 (16.3)	
	>38 weeks	94,963 (32.1)	2,475 (28.2)	
Discharge Year	2015	43,370 (14.7)	931 (10.6)	<0.0001
	2016	47,727 (16.1)	1,227 (14.0)	
	2017	51,324 (17.3)	1,398 (15.9)	
	2018	45,749 (15.5)	1,293 (14.7)	
	2019	44,308 (15.0)	1,483 (16.9)	
	2020	35,445 (12.0)	1,378 (15.7)	
	2021	28,028 (9.5)	1,064 (12.1)	
Renal Replacement Therapy		167 (0.1)	243 (2.8)	<0.0001
Feudtner† Pediatric Complex Chronic Conditions (CCC) Classifications	Any CCC?	144,471 (48.8)	8,261 (94.2)	<0.0001
	# of CCC’s	0.9 ± 1.4	2.9 ± 1.9	<0.0001
	Cardiovascular	40,942 (13.8)	3,839 (43.8)	<0.0001
	Congenital or Genetic	20,450 (6.9)	1,278 (14.6)	<0.0001
	Gastrointestinal	29,877 (10.1)	2,960 (33.7)	<0.0001
	Hematologic or Immunologic	7,061 (2.4)	704 (8.0)	<0.0001
	Malignancy	10,724 (3.6)	213 (2.4)	<0.0001
	Metabolic	14,858 (5.0)	1,293 (14.7)	<0.0001
	Neonatal	70,608 (23.9)	6,187 (70.5)	<0.0001
	Neurologic or Neuromuscular	21,110 (7.1)	1,594 (18.2)	<0.0001
	Renal	16,362 (5.5)	2,498 (28.5)	<0.0001
	Respiratory	16,040 (5.4)	1,383 (15.8)	<0.0001
	Medical Technology (i.e device dependency)	27,051 (9.1)	3,298 (37.6)	<0.0001
	Transplantation	5,510 (1.9)	217 (2.5)	<0.0001

Continuous variables presented as mean ± standard deviation; Categorical variables presented as counts (percentages). †Feudtner et al. BMC Pediatrics 2014, 14:199.

**Abstract 028**

**Durational Phenotype of Cardiac Surgery-associated Acute Kidney Injury after the Norwood Procedure**

Denise C Hasson^1*^, Rebecca A Bertrandt^2^, Huaiyu Zang^3^, David Selewski^4^, Garrett Reichle^5^, David Bailly^6^, Sarah Tabbutt^7^, Catherine Krawczeski^8^, David Winlaw^3^, Stuart L Goldstein^3^, Jeffrey A Alten^3^, Katja M Gist^3^ on behalf of the Neonatal and Pediatric Heart and Renal Outcomes Network (NEPHRON) Investigators

^*1*^ *New York University Langone Health, Hassenfeld Children’s Hospital, New York, NY;* ^*2*^*Medical College of Wisconsin, Children’s Wisconsin, Milwaukee, WI;* ^*3*^*Cincinnati Children’s Hospital Medical Center, Cincinnati, OH;* ^*4*^*Medical University of South Carolina, Charleston, SC;* ^*5*^*University of Michigan Medical School, Ann Arbor, MI;* ^*6*^*Primary Children’s Hospital, Salt Lake City, UT;* ^*7*^*University of California San Francisco, San Francisco, CA;* ^*8*^*Nationwide Children’s Hospital, Columbus, OH*

**Introduction:** Cardiac Surgery-associated Acute Kidney Injury (CS-AKI) is common and associated with adverse outcomes. Prior work from the Neonatal and Pediatric Heart and Renal Outcomes Network (NEPHRON) demonstrated that only stage 3 AKI was associated with mortality. Given persistent AKI has been associated with worse outcomes in this population, we hypothesize that phenotyping CS-AKI based on duration after the Norwood procedure (NP) will associate with morbidity and mortality.

**Methods:** Multicenter retrospective cohort study from NEPHRON of consecutive neonates undergoing the NP. Patients supported by pre- or post-operative ECMO were excluded. CS-AKI was defined using the modified neonatal Kidney Disease: Improving Global Outcomes serum creatinine or urine output (UOP) criteria. UOP criteria were only used in the presence of an indwelling bladder catheter. Transient CS-AKI was defined as resolved by POD 3, and persistent CS-AKI as still present on/after POD 3. Severe CS-AKI was stage 2 or 3.

**Results:** Three-hundred forty-six patients were included. Of 211 (61.0%) with CS-AKI, 127 (36.7%) were transient, 29 (8.4%) severe transient, 78 persistent (22.5%) and 23 (6.6%) severe persistent. Only 5 (1.4%) had delayed onset CS-AKI and were not included in comparative analysis. On univariate analysis, patients that developed persistent CS-AKI had lower intensive care unit admit systolic blood pressure, higher vasoactive inotrope score, higher % fluid overload on POD 0, 1, and more major post-op complications. Of the 15% who underwent prophylactic peritoneal dialysis, 30/51 (58.9%) had transient and 10/51 (19.6%) had persistent CS-AKI. Transient and persistent CS-AKI occurred less frequently in patients exposed to preoperative feeds (Table 1A). Patients with persistent CS-AKI had 3.5 times higher mortality than no AKI. However, on multivariable analysis, persistent CS-AKI was not statistically associated with mortality, respiratory support free days at 28 days and hospital free days at 60 days (Table 1B).

**Conclusion:** After the Norwood Procedure, neither transient nor persistent CS-AKI are associated with important outcomes in this multicenter high-risk cohort after controlling for other risk factors and postoperative complications. Exploring other definitions of clinical CS-AKI that associate with outcomes in this high-risk population is paramount to improving patient care.

**Keywords:** acute kidney injury, Norwood Procedure, AKI phenotype, persistent AKI, transient AKI

**Disclosures:** The authors have no relevant disclosures to report.

**Corresponding Author:** Denise C Hasson, https://orcid.org/0000-0001-6172-4756

**Table 1A, B: Univariate and Multivariate Analyses of Risk Factors and Outcomes for CS-AKI by Durational CS-AKI Phenotype.**

**A.**

**Table:** 

	Overall n=341	No CS-AKI n=136	Transient CS-AKI n=127	Persistent CS-AKI n=78	P value
Pre-op Feeding	127 (37%)	67 (49%)	41 (32%)	19 (24%)	<0.001
Admit Systolic Blood Pressure	68.0 (60.0-75.0)	70.0 (62.0-75.0)	68.0 (62.0-76.0)	65.0 (57.2-70.0)	0.005
Prophylactic peritoneal dialysis in 1^st^ 24 postoperative hours	51 (15%)	11 (8.1%)	30 (24%)	10 (13%)	0.002
1^st^ Negative Fluid Balance POD 0-1	263 (78%)	115 (86%)	100 (79%)	48 (62%)	<0.001
Major Post-op Complications	89 (26%)	25 (18%)	30 (24%)	34 (44%)	<0.001
Post-op Mechanical Ventilation (hours)	140.9 (90.4-218.0)	119.0 (71.0-190.8)	140.9 (88.2-193.3)	185.0 (100.0-378.6)	<0.001
Intensive Care Unit length of Stay (days)	16.1 (10.9-27.9)	14.5 (9.9-23.9)	18.8 (11.5-28.4)	18.2 (11.9-31.1)	0.041
Days Free of Respiratory Support	18.0 (7.0-22.0)	20.0 (13.8-23.0)	18.0 (7.5-22.0)	14.0 (0.0-20.0)	<0.001
Hospital Free Days	28.0 (4.0-39.0)	29.0 (9.0-39.2)	28.0 (9.0-39.5)	18.5 (0.0-38.0)	0.083
Mortality	18 (5.3%)	5 (3.7%)	3 (2.4%)	10 (13%)	0.003

Categorical variables n (%) and Continuous variable median (IQR); P-values calculated using chi-square test or Kruskal–Wallis test. Op-operative, POD-post-operative day.

**B.**

**Table:** 

	No CS-AKI	Transient CS-AKI	Persistent CS-AKI
Mortality	Reference	0.53 (0.09-3.14)	1.72 (0.44-6.75)
Days Free of Respiratory Support	Reference	0.65 (0.41-1.26)	0.72 (0.41-1.26)
Hospital Free Days	Reference	1.01 (0.62-1.65)	1.08 (0.62-1.89)

Persistent and Transient CS-AKI Compared to no CS-AKI. Logistic and ordinal regression models used to calculate odds ratio (95% confidence intervals). Model was adjusted for chromosomal anomalies, needing either pre-operative inotropes or mechanical ventilation, post-operative vasoactive inotrope score >15 on post-operative day 0, 1st post-operative day of negative fluid balance, BTTS as source of pulmonary blood flow, and having major post-op complications or infection.

**Abstract 029**

**Early Detection of Acute Kidney Injury in Neonates after Cardiopulmonary Bypass**

Tennille N. Webb^1*,2^, MD, MSPH, Santiago Borasino^1,2^, MD, MPH, Kristal M. Hock^1^, MSN, RN, Inmaculada Aban^1^, PhD, David Askenazi^1*,2^, MD, MSPH

^*1*^ *The University of Alabama at Birmingham, Birmingham, AL, USA;* ^*2*^*Children’s of Alabama, Birmingham, AL, USA*

**Background:** Prophylactic peritoneal dialysis (PD) in neonates undergoing cardiac surgery with cardiopulmonary bypass (CPB) has proven to be safe and improve outcomes. Understanding which neonates would benefit from prophylactic PD is needed to optimize care. We sought to: 1) determine which neonates would most benefit from prophylactic PD after CPB based on patient-specific characteristics via retrospective analysis; 2) implement a new prophylactic PD protocol based on our retrospective analysis; and 3) determine the effectiveness of our new protocol.

**Methods:** First, we retrospectively evaluated neonates requiring cardiac surgery with CPB from October 2012 through June 2016. We categorized neonates as those who “needed PD” and those who “did not need PD.” We defined “needed PD” as those who had a PD catheter placed in the OR that was used for >48 hours, or those who did not have a PD catheter placed in the OR but in retrospect would have benefited from PD based on predetermined clinical findings. Of the variables examined, only pre-operative serum creatinine ≥ 0.8 mg/dL, pre-operative weight ≤ 2.5 kg, or having an open chest post-operatively were independently associated with “needed PD.” Next, beginning March 2019 we implemented a new prophylactic PD protocol so that only those who met at least one of the three criteria had a PD catheter placed in the OR. Finally, we analyzed the second era experience.

**Results:** In the first era, of the 67 neonates in the “needed PD” group, 9/67 (13.4%) did not have a PD catheter placed in the OR while of the 81 neonates in the “did not need PD” group, 41/81 (50.6%) had a PD catheter placed. Alternatively, in the second era, of the 28 neonates in the “needed PD” group, 0/28 (0%) did not have a PD catheter placed in the OR while of the 69 neonates in the “did not need PD” group, 18/69 (26.1%) had a PD catheter placed.

**Conclusion:** We successfully developed and implemented an evidence-based prophylactic PD protocol. This risk-based protocol has improved our ability to provide prophylactic PD in neonates requiring CPB. Larger prospective studies are needed to further validate our findings.

**Keywords:** acute kidney injury (AKI), cardiopulmonary bypass (CPB), peritoneal dialysis (PD), neonates

**Disclosures:** David Askenazi: consultant and/or receives funding for education/research from Baxter, Nuwellis, Medtronic, Seastar, Bioporto, and Portero; CSO and founder of Zorro-Flow Inc.

**Table 1. 2x2 table of Era 1 and Era 2 need for PD**

**Table:** 

**Era 1**	**Needed PD**	**Did NOT need PD**	**Total**
PD catheter placed (YES)	58 (Group 1)	41 (Group 2)	99
PD catheter placed (NO)	9 (Group 3)	40 (Group 4)	49
Total	67	81	148
**Era 2**	**Needed PD**	**Did NOT need PD**	**Total**
PD catheter placed (YES)	28 (Group 1)	18 (Group 2)	46
PD catheter placed (NO)	0 (Group 3)	51 (Group 4)	51
Total	28	69	97

PD (peritoneal dialysis)

**Abstract 030**

**Peritoneal Dialysis in the Cardiac ICU Throughout the Years…a Descriptive Analysis**

Tennille N. Webb^1*,2^, MD, MSPH, Santiago Borasino^1,2^, MD, MPH, Jessica Potts^1^, RN, BSN, Patrick Phillips^1^, David Askenazi^1*,2^, MD, MSPH

^*1*^ *The University of Alabama at Birmingham, Birmingham, AL, USA;* ^*2*^*Children’s of Alabama, Birmingham, AL, USA*

**Background:** Peritoneal dialysis (PD) is a common modality of kidney support therapy (KST) in smaller patients, especially infants. It has been shown that PD after cardiac surgery improves outcomes; however, most pediatric hospitals do not have protocols in place for managing AKI and fluid overload post-operatively with PD.

**Methods:** In this descriptive analysis, we reviewed children requiring cardiac surgery in the cardiac ICU from October 2012 to December 2020 who received PD. Our PD program practices were examined, including PD patient days per year and PD complications. We also evaluated patient-specific characteristics including age, STAT (Society of Thoracic Surgeons-European Association for Cardio-Thoracic Surgery) score, need for cardiopulmonary bypass (CPB), open chest and volume status.

**Results:** Our analysis revealed PD patient days ranged from 45 to 228, with 228 PD patient days in 2013 and 139 PD patient days in 2020. Once on PD, most patients required PD for a duration of 2-7 days. There was minimal PD catheter dysfunction with the most issues in 2013 with 4 complications per 100 patient days (6 failed to fill/drain, 2 peritonitis and 1 leaking), 2019 with 3 complications per 100 patient days, and years 2018 and 2020 with <1 complication per 100 patient days. After receiving 48 hours of PD the majority of patients had <10% fluid overload and only one patient with >20% fluid overload. Most of the patients who required PD were neonates with 89.7% in 2013 and 96.6% neonates in 2020. The majority of patients with PD had STAT 4 scores from 2013 through 2017 and STAT 5 scores from 2018 through 2020. As for cardiopulmonary bypass (CPB), 80% of PD patients required CPB. Lastly, 40% of PD patients had an open chest post-operatively.

**Conclusion:** We described nine years of PD patient data in our cardiac ICU. There were very minimal complications associated with performing PD at our center. As cardiac surgical complexity increases so will the need for PD in these patients. Developing strong relationships between the nephrologists, cardiac intensivists and cardiac surgeons has played a significant role in the success of our PD program.

**Keywords:** peritoneal dialysis (PD), cardiac surgery, complications

**Disclosures:** David Askenazi: consultant and/or receives funding for education/research from Baxter, Nuwellis, Medtronic, Seastar, Bioporto, and Portero; CSO and founder of Zorro-Flow Inc.

**Figure 1. PD Patient Days per Year** 
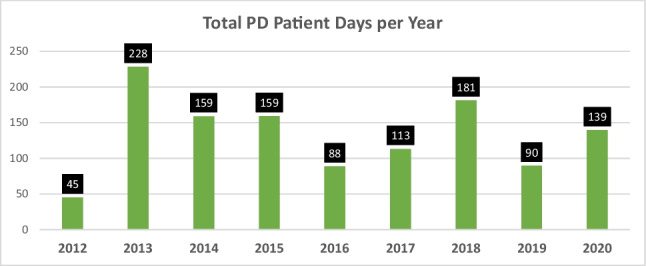


**Abstract 031**

**Urine Cystatin C Predicts Acute Kidney Injury following Pediatric Cardiopulmonary Bypass**

Susan D. Martin^1^ MD, Michael F. Swartz^2^ PhD, Maher Abadeer^3^ MD, Alison L. Kent^1,4^ MD, George J. Schwartz^1^ MD, Patrick Brophy^1^ MD, George M. Alfieris^2^ MD, Jill M. Cholette^1^ MD

^*1*^ *Department of Pediatrics;* ^*2*^*Department of Surgery, Golisano Children’s Hospital, University of Rochester Medical Center, Rochester, NY, USA;* ^*3*^*Department of Pediatrics, Children’s Medical Center Dallas, University of Texas Southwestern, Dallas, TX, USA;* ^*4*^*College of Health and Medicine, Australian National University, Canberra, ACT, Australia*

**Background:** Early Acute Kidney Injury (AKI) following cardiopulmonary bypass (CPB) is often diagnosed on post-operative day one and is associated with an increased morbidity and mortality. Cystatin C (CysC), is a biomarker synthesized by all nucleated cells with limited catabolism during AKI. Although data in adults suggest Urine (U)CysC is an effective biomarker to predict AKI, if UCysC can predict early AKI in children following CPB is not known. We hypothesized that UCysC measured at 6 hours after CPB would better predict early AKI than standard measures of Urine Output (UOP) and Serum Creatinine (SCr).

**Methods:** Prospective observational study of children (< 18 years) requiring CPB during cardiac surgery. UCysC was measured at 6 hours following CPB initiation. Clinical variables including serum creatinine (SCr), CysC, and urine output (UOP) were measured at ICU admission and during the post-operative day 0. Subjects were divided into two groups: AKI and non-AKI based upon the Kidney Disease Improving Global Outcomes (KDIGO) classification. A Receiver Operating Characteristic (ROC) curve examined the association between SCr, UOP, and 6-hour UCysC and the development of early AKI by measuring the area under the curve (AUC).

**Results:** Among 70 children, AKI occurred in 28.5% (n=20) by post-operative day one. Pre-operative demographics and baseline renal function were similar between groups. The serum creatinine measured at ICU admission following surgery was similar between groups (AKI:0.31 ± 0.1mg/dL vs NonAKI:0.36 ± 0.2 mg/dL; p=0.4). Urine output during post-operative day 0 was significantly lower within the children who developed early AKI (AKI:1.1 (IQR:0.8, 2.1 cc/kg/hr) vs NonAKI:1.8 (IQR:1.3, 2.6 cc/kg/hr) p < 0.01). Six-hour UCysC was significantly greater within the AKI group (Figure 1). ROC curves demonstrated that 6 hour UCysC had the greatest predictive ability (AUC=0.745; p=0.001) in identifying early AKI. Multivariate analysis confirmed that 6-hour UCysC (Odds Ratio:0.99, 95% CI:0.99, 1.001; p=0.03) was independently associated with early AKI when comparing SCr and UOP.

**Conclusions:** Six-hour UCysC independently predicts early AKI in children following CPB and may allow for earlier treatment and diagnoses of kidney injury in this critically ill population. 
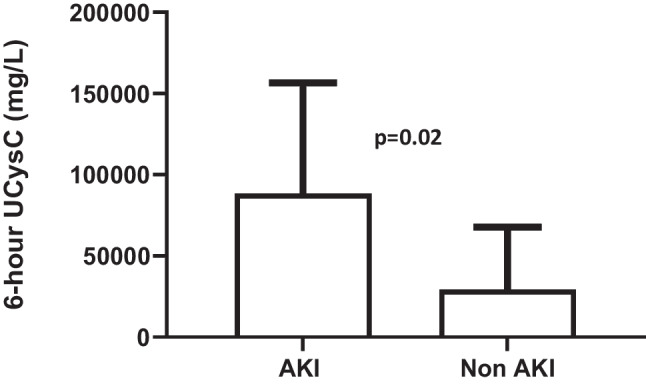


**Figure Legend**: Six-hour Urine Cystatin C (UCysC) levels in children who developed Acute Kidney Injury (AKI).

**Keywords:** kidney injury, pediatric, biomarker, cardiac surgery

**Disclosures:** Maher Abadeer and Jill Cholette received partial financial support from the Clausen and Bradford Fellowship Award, University of Rochester Medical Center, Rochester, NY

Maher Abadeer and Alison Kent received partial financial support from bioMérieux Nephrocheck Funding. bioMérieux SA.

The authors listed have no conflicts of interest in relation to this research

**References:** None

**Abstract 032**

**Increasing Incidence of AKI and AKI requiring dialysis in a National Cohort**

Alexander Kula^1^, MD, MHS, Shina Menon^2^ MD

^*1*^ *Ann and Robert H. Lurie Children’s Hospital of Chicago and Northwestern University, Chicago, IL;* ^*2*^*Seattle Children’s Hospital and University of Washington, Seattle, WA*

**Background:** Acute Kidney Injury (AKI) is common during pediatric hospitalizations and associated with adverse short-term outcomes, including need for dialysis. There are limited epidemiologic data on the rate of AKI requiring dialysis (AKI-D) over the years. The purpose of this study was to characterize rates of pediatric AKI and AKI-D across the United States.

**Methods:** Longitudinal analysis of the Kids Inpatient Database from 1997 to 2016 was done. AKI events were identified using International Classification of Diseases Clinical Modification (ICD-CM) codes, Ninth Revision (1997-2012) and Tenth Revision (2016). Use of dialysis during hospitalization was assessed using ICD-CM procedure codes. Individuals with end-stage kidney disease (ESKD) were identified using a validated combination of ICD-CM diagnosis and procedure codes. Study data was weighted for estimates of yearly incidence totals and rates. For more accurate estimation of rate of hospitalizations complicated by AKI or requiring dialysis, we excluded pediatric hospitalizations for uncomplicated newborn birth.

**Results:** Over the study period, there were an estimated 49,228,048 pediatric discharges. After excluding all hospitalizations that represented uncomplicated newborn births, a total of 22,059,242 hospitalizations were included in this analysis. Nationwide, the yearly incidence of AKI and AKI-D increased from 1997 (AKI**:** 5,536 cases/year, AKI-D: 1,062 cases/year) to 2016 (AKI: 42,644 cases/year, AKI-D: 2,290 cases/year). The rates of hospitalizations with AKI and AKI-D also increased from 1997 (AKI: 19 cases per 10000 discharges, AKI-D: 3.7 per 10000 discharges) to 2016 (AKI: 171 per 10000 discharges, AKI-D: 9.2 per 10000 discharges).

**Conclusion:** This preliminary analysis from the Kids Inpatient Database shows an increase in the absolute number, and the rate of pediatric hospitalizations complicated by AKI and AKI-D 1997 to 2016. While some of the increase in rates of AKI may be explained by an increase in awareness and documentation, there is near 3-fold increase in rates of AKI-D. More detailed analysis is needed to evaluate the reasons behind such an increase. Given our understanding of the long-term impact of pediatric AKI, it would be important to assess for preventable causes, and to optimize care for young individuals who experience these outcomes.

**Keywords:** Epidemiology, Acute Kidney Injury, Acute Dialysis

**Disclosures:** The authors declare no competing interests.

**Corresponding Author:** Alex Kula

**Figure 1:** The cumulative yearly incidence of pediatric hospitalizations complicated by AKI or AKI-D per 10,000 discharges from 1997 to 2016 
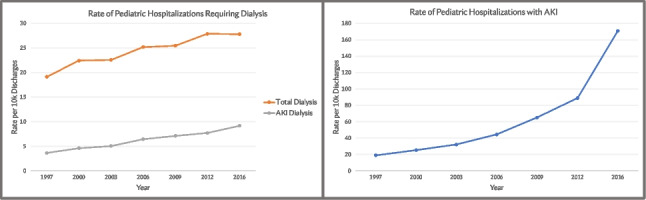


**Abstract 033**

**Incidence of Recurrent Acute Kidney Injury (AKI) in Children with Previous Critical Illness and Accompanying AKI: Natural History and Outcomes**

Hollis Johnson^1*^, Rebekah Wakeman^2^, Julia Steinke^3^, Freddie Hildreth^4^, Richard Hackbarth^3^

^*1*^ *Spectrum Health/Michigan State University Pediatric Residency, Grand Rapids, MI, USA;* ^*2*^*MyMichigan Health, Midland, MI, USA;* ^*3*^*Helen DeVos Children’s Hospital, Grand Rapids MI, USA;* ^*4*^*Spectrum Health Office of Research, Grand Rapids, MI, USA*

**Background:** AKI contributes significantly to short and long-term outcomes following critical illness in children. More than a quarter of pediatric intensive care unit (PICU) patients experience AKI. The initial insult of AKI in PICU patients may predispose them to recurrence in future hospitalizations but literature is limited on this potential association. Our study aimed to explore this potential association and possible risk factors for subsequent kidney injury.

**Methods:** A retrospective, single-center, observational study was conducted comparing patients with AKI during first admission to the PICU to a control group without AKI on first admission. The study included patients less than 19 years old on initial admission between January 1, 2014 to December 31, 2020. Patients with pre-existing kidney disease, death during first admit, or no readmissions were excluded. Kidney Disease Improving Global Outcomes criteria were used for staging of AKI. Pediatric Risk of Mortality (PRISM) scores classified severity of illness and Nephrotoxic Injury Negated by Just in time Action (NINJA) criteria were used to define nephrotoxic drug exposure.^1^ There were 91 study patients and 94 age-matched controls. Analyses of quantitative and qualitative data were done by t-test and chi-squared test respectively.

**Results:** There was a significant association between AKI during the initial PICU stay and future recurrence(s) of AKI (p <.0001) (see Table 1 for details). The AKI positive group were more likely to develop chronic kidney disease (CKD) (p <.0001). This group had a higher proportion of cardiovascular disease (p <.0001) and higher PRISM scores (p <.0001).

**Conclusions:** Patients with AKI during their first PICU admission are at significantly increased risk for recurrence and for development of CKD. Patients admitted for cardiovascular diagnoses and with an elevated PRISM score are more likely to develop AKI. The results suggest these patients may benefit from nephrology follow up and kidney protective strategies during future hospitalizations.

**Keywords: **pediatric, critical illness, recurrent AKI, chronic kidney disease

**Disclosures: **No competing interests or financial disclosures for any of the authors.

**Corresponding Author:** Hollis M. Johnson, https://orcid.org/0000-0003-4621-6280

**References**

1. Goldstein SL, *et al*. A sustained quality improvement program reduces nephrotoxic medication-associated acute kidney injury. *Kidney International* 2016; 90:212-221. doi: 10.1016/j.kint.2016.03.031

**Table 1. Demographic and Acute Kidney Injury (AKI) Recurrence Outcome Data**

**Table:** 

**Variable**	**AKI Positive (n=91 patients)**	**AKI Negative (n=94 patients)**	**P-value**
Patient Sex			
Female	38(41.76)*	40(42.55)	0.9548
Male	52(57.14)	52(55.32)	
Non-Binary	1(1.10)	2(2.13)	
AKI Recurrence One or more episodes			
No	43(47.25)	85(90.43)	<0.0001
Yes	48(52.75)	9(9.57)	
AKI Recurrence More than 2 episodes			
No	58(63.74)	91(96.81)	<0.0001
Yes	33(36.26)	3(3.19)	
Continuous Variable	**Mean** **+** **SD**	**Mean** **+** **SD**	
Age(years)	6.09 ± 6.70	5.53 ± 5.18	0.5245
Height(cm)	101.30 ± 49.29	106.50 ± 38.17	0.4251
Weight(kg)	27.53 ± 29.88	23.97 ± 21.23	0.3533

*Percentages expressed in parentheses

**Abstract 034**

**Incidence, Risk Factors, and Outcomes Associated with Recurrent Acute Kidney Injury in Neonates: A Report from the AWAKEN study**

Austin D. Rutledge^1^, DO, Russell L. Griffin^2^, PhD, Katherine Vincent^1^, DNP, NNP, David J. Askenazi^2^, MD, MPH, Jeffrey L. Segar^3^, MD, Juan C. Kupferman^4^, MD, MPH, David T. Selewski^1^, MD, MSCR, Heidi J. Steflik^1^, MD, MSCR; On behalf of the Neonatal Kidney Collaborative (NKC)

^*1*^ *Medical University of South Carolina, Charleston, SC, USA;* ^*2*^*University of Alabama at Birmingham, Birmingham, Alabama, USA;* ^*3*^*Medical College of Wisconsin, Milwaukee, WI, USA;* ^*4*^*Maimonides Medical Center, Brooklyn, NY, USA*

**Background:** Multiple episodes of neonatal acute kidney injury (AKI) in a single patient, “recurrent acute kidney injury” (rAKI), is understudied. Using a multicenter cohort, we sought to determine the incidence, risk factors, and clinical outcomes associated with rAKI in critically ill neonates. We hypothesized that rAKI would be associated with increased mortality and longer length of stay (LOS) compared to neonates with a single AKI (sAKI) or no AKI episodes.

**Methods:** A secondary analysis of the AWAKEN multicenter retrospective cohort study was performed using the neonatal modified KDIGO criteria to identify rAKI. Determination of each rAKI episode required a complete return to baseline serum creatinine. Comparisons were made between those with rAKI, sAKI, and no AKI to identify risk factors for rAKI. Associations between rAKI and mortality as well as LOS were evaluated using multivariable, general estimating equation logistic and linear regression, respectively, to account for clustering by study center. Models were stratified by gestational age (GA) to assess potential effect modification.

**Results:** Of the 605 infants with AKI in AWAKEN (2,162 enrolled), 133 (22%) developed rAKI. Risk factors for rAKI included ethnicity, GA, birthweight, and APGAR scores. Compared to infants without AKI, infants with rAKI had higher mortality (3.28, 95% CI 1.19-9.04) and longer LOS (rAKI: 36.7±20.4 days vs. no AKI: 21.7±17.6 days, adjusted p-value <0.0001). When comparing infants with rAKI to those with sAKI, there was no difference in overall adjusted odds of mortality, but infants with rAKI stayed on average 11.7 days longer (95% CI 6.1-17.4) after adjusting for multiple potential confounders. The association between rAKI and mortality, compared to non-AKI infants, was highest among ≥36 weeks GA (OR 9.54, 95% CI 3.27-27.89) while LOS was consistently highest among the rAKI infants across GA strata (Table).

**Conclusion:** rAKI was independently associated with longer LOS and increased mortality when compared to no AKI, particularly for full-term infants. The results support our hypothesis and suggest that recurrence of AKI is particularly detrimental, warranting further study. rAKI is likely an important clinical distinction and careful monitoring for recurrence after an initial AKI episode is warranted.

**Keywords:** neonatal AKI, recurrent, AWAKEN, Neonatal Kidney Collaborative

**Disclosures:** DA consults and/or receives funding for education/research from Baxter, Nuwellis, Medtronic, Seastar, Bioporto, Portero, CSO and founder of Zorro-Flow, Inc. HJS received a grant from Baxter.

**Corresponding author:** Austin D. Rutledge, ORCID iD 0000-0002-9276-6956

**Table.** Crude and Adjusted Odds Ratios (OR) and 95% Confidence Intervals (CIs) for the Association between No Acute Kidney Injury (AKI), single AKI, and recurrent AKI and Death as well as Length of Stay

**Table:** 

	**Death** **(%)**	**Crude OR** **(95% CI)**	**p-value**	Adjusted* OR **(95% CI)**	**p-value**
**MORTALITY**					
**Overall**					
No AKI (n=1,557)	24 (1.5)	Referent		Referent	
Single AKI (n=472)	43 (9.1)	**6.40 (3.44-11.93)**	**<0.0001**	**4.16 (2.04-8.48)**	**<0.0001**
Recurrent AKI (n=133)	16 (12.0)	**8.74 (3.98-19.15)**	**<0.0001**	**3.28 (1.19-9.04)**	**0.0217**
**GA <28 weeks**					
No AKI (n=145)	10 (6.9)	Referent		Referent	
Single AKI (n=85)	22 (25.9)	**4.71 (1.89-11.76)**	**0.0009**	**3.77 (1.34-10.58)**	**0.0118**
Recurrent AKI (n=46)	8 (17.4)	**2.84 (1.08-7.50)**	**0.0349**	1.55 (0.48-5.01)	0.4674
**GA 28-35 weeks**					
No AKI (n=790)	7 (0.9)	Referent		Referent	
Single AKI (n=139)	12 (8.6)	**10.57 (5.00-22.36)**	**<0.0001**	**8.54 (3.42-21.32)**	**<0.0001**
Recurrent AKI (n=29)	1 (3.4)	4.00 (0.45-35.39)	0.2134	2.18 (0.16-30.38)	0.5636
**GA 36+ weeks**					
No AKI (n=622)	7 (1.1)	Referent		Referent	
Single AKI (n=248)	9 (3.6)	**3.31 (1.30-8.40)**	**0.0119**	2.27 (0.88,5.82)	0.0886
Recurrent AKI (n=58)	7 (12.1)	**12.06 (4.07-35.76)**	**<0.0001**	**9.54 (3.27,27.89)**	**<0.0001**
	**Mean LOS** **(SD)**	**Crude β** **(95% CI)**	**p-value**	Adjusted* β **(95% CI)**	**p-value**
**LENGTH OF STAY**					
**Overall**					
No AKI (n=1,476)	21.7 (17.6)	Referent		Referent	
Single AKI (n=410)	21.7 (18.5)	0.04 (-3.00,3.10)	0.977	**2.38 (0.34,4.43)**	**0.0224**
Recurrent AKI (n=80)	36.7 (20.4)	**14.97 (10.50,19.44)**	**<0.0001**	**14.13 (8.28,19.97)**	**<0.0001**
**GA <28 weeks**					
No AKI (n=79)	48.6 (21.9)	Referent		Referent	
Single AKI (n=42)	37.2 (26.2)	**-11.37 (-22.13,-0.60)**	**0.0385**	-10.17 (-20.78,0.45)	0.0606
Recurrent AKI (n=15)	44.1 (24.7)	-4.45 (-15.35,6.46)	0.4239	-2.35 (-14.54,9.83)	0.705
**GA 28-35 weeks**					
No AKI (n=776)	25.5 (16.4)	Referent		Referent	
Single AKI (n=130)	27.2 (20.2)	1.74 (-1.75,5.24)	0.3286	**4.30 (0.82,7.78)**	**0.0154**
Recurrent AKI (n=20)	42.1 (20.6)	**16.61 (6.26,26.95)**	**0.0017**	**13.71 (4.23,23.18)**	**0.0046**
**GA 36+ weeks**					
No AKI (n=621)	13.5 (12.9)	Referent		Referent	
Single AKI (n=238)	16.0 (12.5)	2.49 (-0.06,5.04)	0.056	**3.05 (0.42,5.68)**	**0.0229**
Recurrent AKI (n=45)	31.7 (17.7)	**18.24 (12.42,24.07)**	**<0.0001**	**18.34 (12.53,24.15)**	**<0.0001**

*Models adjusted for length of stay adjusted for gestational age (unless stratified by gestational age), maternal ethnicity, birthweight, 5-minute APGAR, admission for prematurity, admission for respiratory failure, admission for sepsis evaluation, amniotic fluid levels, maternal diabetes, maternal pre-eclampsia, multiple gestations, maternal steroid use, and maternal hypertension medication use.

**Abstract 035**

**Safety and Timeliness of Remote Initiation of Continuous Renal Replacement Therapy using Telemedicine**

Michelle C. Starr^1*,2^, Kathleen E. Altemose^1^, Jessalynn Parsley^1^, Daniel T. Cater^1^, David S. Hains^1^, Danielle E. Soranno^1^

^*1*^ *Indiana University School of Medicine, Indianapolis, IN, USA;* ^*2*^*Center for Pediatric and Adolescent Comparative Effectiveness Research, Indiana University, Indianapolis, IN, USA*

**Background:** Our acute dialysis program transitioned some continuous renal replacement therapy (CRRT) initiations to telemedicine to improve timeliness and to minimize COVID-19 transmission risk. For CRRT initiations during nights or weekends, the decision to remote start is made by the nephrology and critical care physicians on a patient-by-patient basis. Using a secure video telemedicine platform, nephrology providers oversee the entire initiation process in collaboration with the in-person critical care physician. While the demand and introduction of telemedicine would appear appropriate and acceptable for many clinical settings, the safety and timeliness of remote initiation of CRRT is undescribed.

**Methods:** We conducted a retrospective analysis of a single-center process improvement project. Information on patient characteristics and CRRT runs were extracted from the electronic health record. Provider attitudes and practices were assessed using survey.

**Results:** Since January 2021, there have been 76 CRRT circuit initiations in patients not previously receiving CRRT, with 13% (22/76) initiated remotely. Patient characteristics, including patient age, weight at initiation, underlying diagnosis and treatment location did not differ between in-person CRRT initiation and remote initiation. CRRT prescription, including modality of CRRT and anticoagulation did not differ. Only Prismaflex starts occurred remotely; no Aquadex starts occurred remotely (**Table 1).** CRRT remote initiations were timelier, occurring on average 3.6 hours after decision to initiate therapy compared to 5.3 hours for night and weekend in-person CRRT starts (*p*=0.014). The complication rate did not differ between telemedicine and in-person starts (16% vs. 22%, *p*=0.72). Remote initiations were widely acceptable to providers. Of 38 respondents to our survey (15 critical care physicians, 10 pediatric nephrologists and 13 critical care CRRT nurses), remote starts were considered equally safe to in-person starts (4.4/5), nephrology was thought to be accessible during initiation (4.5/5) while improving workforce wellness and decreasing burnout (4.7/5).

**Inclusion:** In appropriately selected patients, remote initiation of CRRT using telemedicine is a timely and safe option for initiating dialytic therapy. Further standardization of remote initiation of CRRT should be considered in appropriate patients to improve the timely delivery of CRRT and may improve nephrology workforce wellness.

**Keywords:** continuous renal replacement therapy, dialysis, telemedicine, critical care nephrology

**Disclosures:** The authors declared no competing interests.

**Corresponding Author:** Michelle C. Starr, https://orcid.org/0000-0001-9412-8950

**References:** None

**Table 1.** Patient characteristics and CRRT details presented by remote start status.

**Table:** 

	Remote Starts (n=22)	In-Person Starts (n=54)	In-Person Starts (Nights and Weekends) (n=27)	p-value^1^	p-value^2^
**Patient Characteristics**					
Age, (years), mean (SD)	12.1 (7)	10.1 (9)	9.9 (9)	.33	.38
Male, n (%)	15 (68)	28 (52)	12 (44)	.19	.10
Primary Disease, n (%)					
Oncologic	7 (32)	8 (15)	5 (19)	.53	.45
Pulmonary/Respiratory Failure	2 (8)	8 (15)	4 (16)		
Congenital Heart Disease	4 (16)	3 (6)	2 (8)		
Bone Marrow Transplant	1 (4)	8 (15)	4 (16)		
Liver failure	3 (12)	4 (7)	1 (4)		
Kidney disease	2 (8)	10 (18)	6 (22)		
Other	3 (12)	13 (24)	5 (19)		
Weight at initiation, n (%)					
<10kg	5 (23)	10 (18)	5 (18)	.84	.63
10-20kg	2 (8)	7 (14)	5 (18)		
>20kg	15 (68)	37(68)	17 (64)		
Fluid overload at initiation, n (%)					
<10%	8 (36)	22 (41)	12 (44)	.51	.83
10-20%	9 (41)	15 (28)	9 (33)		
>20%	5 (23)	17 (31)	6 (22)		
Location, n (%)					
PICU	18 (82)	50 (93)	25 (93)	.17	.25
CVICU	4 (18)	4 (7)	2 (7)		
**CRRT Characteristics**					
Machine, n (%)					
Prismaflex	22 (100)	44 (81)	21 (78)	.12	.048
Aquadex	0	10 (19)	6 (22)		
Modality, n (%)					
SCUF	2 (8)	5 (9)	2 (7)	.88	.99
CVVH	10 (46)	28 (52)	14 (52)		
CVVHD	0	1 (2)	0		
CVVHDF	10 (46)	20 (37)	11 (41)		
Filter, n (%)					
HF-1000	15 (68)	29 (54)	15 (56)	.37	.39
HF-20	5 (23)	14 (26)	6 (22)		
UF-500	0	10 (18)	6 (22)		
H-50 (ECMO)	2 (8)	1 (2)	0		
Anticoagulation, n (%)					
Citrate	18 (82)	40 (74)	23 (85)	.65	.95
Heparin	3 (12)	8 (15)	3 (11)		
None	0	6 (11)	1 (4)		
**Initiation Information**					
Time to Initiation (hours), mean (SD)	3.6 (1.9)	5.6 (2.6)	5.3 (2.5)	.002	.014
Complications, n (%)	4 (16)	8 (15)	6 (22)	.71	.72
Normal saline bolus	2 (8)	7 (14)	4 (16)		
Increased vasopressors	2 (8)	4 (7)	3 (11)		
Calcium bolus	0	1 (2)	1 (4)		
Vasopressor Bolus	0	0	0		
**Outcomes**					
Filter Life (hours), mean (SD)	38.7 (30)	37.0 (24)	41.2 (23)	.80	.74
Filter Life >60 hours, n (%)	5 (23)	15 (28)	8 (30)	.65	.59
CRRT Treatment Time (hours), mean (SD)	168 (19)	293 (47)	343 (97)	.16	.08
Death, n (%)	8 (36)	21 (39)	11 (41)	.64	.78

^1.^Comparing remote starts to all in-person starts

^2.^Comparing remote starts to night and weekend in-person starts

**Abstract 036**

**Ultrafiltration Achieved Using the Manual Single Lumen Alternating Micro-batch Device**

Apaara K Chawla^1*^, Giovanni Ceschia^2^, Jolyn Morgan^3^, James Rose^3^, Michael Santoro^4^, and Denise C Hasson^3^

^*1*^ *Francis Parker High School, San Diego, CA;* ^*2*^*University of Padua, Padua, Italy;* ^*3*^*Cincinnati Children’s Hospital Medical Center, Cincinnati, OH;* ^*4*^*Miami University, Oxford, OH*

**Background:** Fluid overload remains associated with worse outcomes in critically ill patients. This impact is exacerbated in low resource areas with limited dialysis access. A single lumen alternating micro-batch (SLAMB) dialysis device performs ultrafiltration and renal replacement therapy (RRT) using single lumen access, a low-cost set of bags and tubing, premade fluids, and a dialysis filter. A manual variation of the SLAMB (mSLAMB) has been shown to work without the use of electricity, batteries, or a pump. We hypothesize that mSLAMB can perform effective ultrafiltration via progressive manual fluid removal.

**Methods:** Four *in vitro* experiments were conducted wherein expired human packed red blood cells were diluted with 0.9% saline to a hematocrit of 20± 3%. mSLAMB was connected to a polyflux 6H filter, but no dialysate/hemofiltration fluids were utilized. In each cycle, 200mL batches were run forward and reverse through the filter. Different percentages of batch volume were removed with a syringe: 20% on reverse pass only, 15% per pass, and 20% per pass. Eight to 11 cycles were conducted per experiment, and the total fluid removed, hematocrit, and potassium were measured.

**Results:** We removed 40-80 mL of fluid via active manual ultrafiltration per cycle, and 0-20 mL per cycle of passive ultrafiltration occurred as well. There was a generally linear relationship between the increase in cumulative ultrafiltration and hematocrit, as evidence by a slow rise in hematocrit with each cycle (Figure 1). The hematocrit difference after 10 cycles was 2.4%, 9%, 10.5%, and 13.5% (the latter after only 8 cycles) with 20%, 30%, 30%, and 40% batch volume removed. Actual hematocrits obtained with 30% batch volume removal closely mirrored predicted hematocrit rise with each cycle.

**Conclusion:** In these pilot experiments, mSLAMB demonstrated effective and accurate ultrafiltration. In patients with fluid overload as their sole indication for dialysis, mSLAMB may increase availability and access to life preserving fluid removal treatment in low-resource settings.

**Keywords:** Acute kidney injury, fluid overload, ultrafiltration, low resource areas, renal replacement therapy

**Disclosures:** Jolyn Morgan is a consultant for Medtronic.

**Corresponding Author:** Apaara K Chawla, Francis Parker High School, San Diego, CA, 92111 
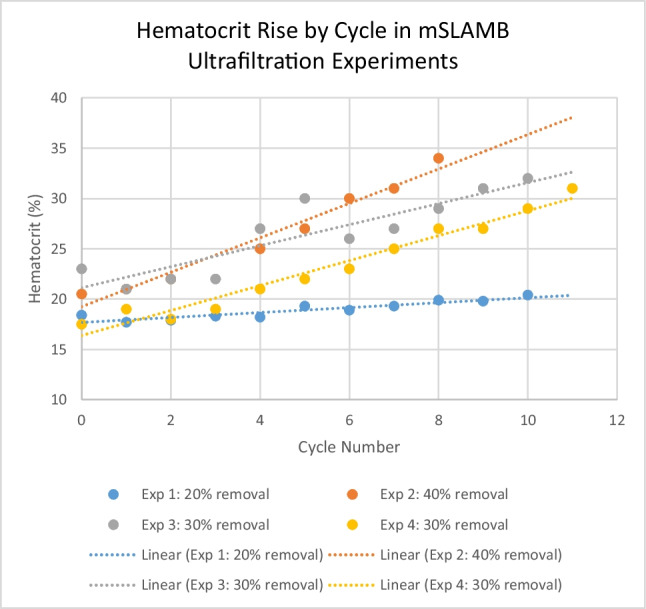


**Abstract 037**

**Improving clinician confidence in performing neonatal dialysis through the Neonatal and Infant Course for Kidney Support**

Kara Short^1*^ MSN, CRNP, CPNP-PC, Daryl Ingram^1^ BSN, RN, CDN, Jessica Potts^1^ BSN, RN, Carrie Norwood^1^ MSHI, BSN, RN, CPN, CHSE, Chrystal Rutledge^1^ MD, Nancy Tofil^1^ MD, David Askenazi^1^ MD, MsPH

^*1*^ *Children’s of Alabama, University of Alabama at Birmingham, Birmingham, Alabama*

**Background:** Since 2012, the Pediatric and Infant Center for Acute Nephrology at Children’s of Alabama (COA), has cared for 415 patients requiring extra-corporeal Continuous Kidney Support Therapy (CKST) over 7906 patient days. We have performed CKST on 184 babies in our PICU, NICU and CVICU over 3619 patient days. Of those 184 babies <10kg, 11 patients were <2kg, 58 were 2-3kg, 49 were 3-4kg, and 66 were 4-10kg. In addition, we have performed peritoneal dialysis on 255 infants for 1083 patient days in our CVICU.

In the last few years, other institutions have begun developing their own programs and our team was contacted with questions rather frequently. We put together a course to share our knowledge about kidney support therapy and named it the Neonatal and Infant Course for Kidney Support (NICKS). We debuted virtually in July 2020. Using didactic sessions, hands-on skills sessions and simulations over one and a half days, the overarching goal for NICKS is to help programs acquire the knowledge and skills to be successful.

**Methods:** We have held eight courses since July 2020. We have hosted 340 participants from 90 cities in 14 countries and 32 US states. Of those participants, 35% were nurses, 10% were advanced practice providers and 55% were physicians, with 49% specializing in nephrology, 30% in neonatology and 21% in intensive care. We assessed comfortability with initiating KST on a 4kg, 2kg, and 1kg baby. 283 participants responded to our pretest and 276 participants responded to our posttests.

**Results:** The results of the pretest and posttest confirm that our participants are more comfortable with starting babies on continuous kidney support therapy after attending our course.

**Conclusions:** The NICKS course has provided education for 340 participants using a multidisciplinary approach. The level of comfort for caring for neonates increases after the course. We plan to continue this course in the future.

**Keywords:** Continuous Kidney Support Therapy (CKST), Neonatal and Infant Course for Kidney Support (NICKS), Comfortability initiating KST

**Disclosures:** D. Askenazi is consultant and/or receives education/research funds from Baxter, Nuwellis, Medtronic, Seastar, Bioporto, Portero. He is the CSO and founder of Zorro-Flow Inc. D. Ingram is a consultant for Nuwellis.

**Figure 1.** Pretest and posttest comfortability in 4kg, 2kg and 1kg patients. 
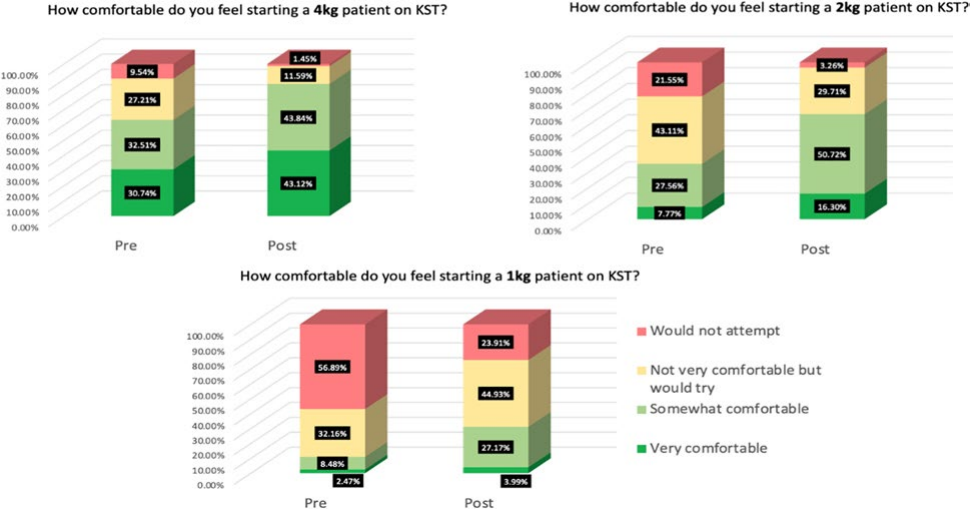


**Abstract 038**

**The threshold of viability in neonates with congenital kidney failure has changed: A case series of four neonates who received extracorporeal membrane oxygenation in the first postnatal week**

Kara Short^1*^ MSN, CRNP, CPNP-PC; Martha McBride^1^ MSN, CRNP, NNP, Scott Anderson^1^ MD, Joe Esparaz^1^ MD, MPH, Kathleen Palmer^1^ MSN, CRNP, NNP, Rachel Miller^1^ MSN, CRNP, FNP, NNP, Daryl Ingram^1^ BSN, RN, CDN, Traci Henderson^1^ RPh, Janelle Schirmer^1^ RD, LD, Carl Coghill^1^ MD, Brian Sims^1^ MD, PhD, David Askenazi^1^ MD, MsPH

^*1*^ *Children’s of Alabama, University of Alabama at Birmingham, Birmingham, Alabama*

**Background:** Neonates born with congenital kidney failure can have severe pulmonary hypoplasia and pulmonary hypertension. Historically, extracorporeal membrane oxygenation (ECMO) has been avoided in patients with congenital kidney failure and associated pulmonary hypoplasia due to concerns regarding irreversibility. Since 2016, 31 neonates have been admitted to the Neonatal Intensive Care Unit at Children’s of Alabama with congenital kidney failure and pulmonary hypoplasia requiring kidney support therapy. Four of these patients were placed on ECMO for pulmonary hypoplasia/hypertension unresponsive to conventional interventions in the first postnatal week. The primary aim of this case series is to describe the clinical courses and long-term outcomes in this unique patient group.

**Methods:** This case series highlights the clinical courses of four neonates with congenital kidney failure, severe pulmonary hypoplasia/hypertension refractory to conventional therapies who were supported with ECMO. Pre and postnatal diagnoses, ECMO course details, kidney support therapy methods, complications, procedures, and long-term outcomes were evaluated.

**Results:** Of the four patients, one was diagnosed with posterior urethral valves (PUV), one with bilateral renal dysplasia, and two with autosomal recessive polycystic kidney disease (ARPKD). Gestational age ranged from 35 weeks 6 days to 37 weeks 6 days. Birth weight range was 2740 grams to 3140 grams. Total days on ECMO ranged from 4 to 23 days. All patients received kidney support therapy by one week of life. Comorbidities and long-term outcomes are detailed in the full case study. All survived to discharge and are still living today. Pulmonary hypertension has resolved in all patients. Two of the four require no oxygen support, and the younger two patients are on nocturnal oxygen only. Two of these patients have received a kidney transplant, one patient remains on the waiting list for transplant, and one patient is awaiting transplant evaluation.

**Conclusions:** Patients with congenital kidney failure with pulmonary hypoplasia and severe pulmonary hypertension are compatible with life, even if they require organ support with ECMO. The multidisciplinary team that can work together to provide respiratory, kidney and comprehensive medical support is necessary to have favorable long-term outcomes. Further investigation into strategies to achieve best outcomes is needed.

**Keywords:** extracorporeal membrane oxygenation (ECMO), kidney support therapy (KST), pulmonary hypertension, pulmonary hypoplasia, outcomes

**Disclosures:** D. Askenazi is consultant and/or receives education/research funds from Baxter, Nuwellis, Medtronic, Seastar, Bioporto, Portero. He is the CSO and founder of Zorro-Flow Inc. D. Ingram is a consultant for Nuwellis.

**Table 1.** Comprehensive medical treatment summary of cohort

**Table:** 

	Patient 1	Patient 2	Patient 3	Patient 4
Baseline diagnosis	PUV	Renal dysplasia	ARPKD	ARPKD
Gestational age at birth	37 weeks 0 days	35 weeks 6 days	37 weeks 1 days	37 weeks 0 days
Birth weight	2970	2740	2840	3140
APGAR 1 min	7	7	4	-
APGAR 5 min	8	7	5	8
Surfactant given	Yes	Yes	Yes	Yes
DOL started on ECMO	1	1	2	7
Total days on ECMO	6	4	11	23
DOL started on KST	6	4	6	5
Days on CVVH via modified Aquadex	161	189	144	74
Days on ventilator	66	60	51	70
Days on high frequency oscillatory ventilation (HFOV)	2	1	1	20
Days on oxygen	202	673	Nocturnal only	Nocturnal only
Days on inhaled nitric oxide	105	22	1	50
Days on vasopressor support	23	27	6	41
Days on antihypertensives	0	0	11	72
Days on sedation drips	75	75	28	83
Days on paralytic drips	2	2	0	0
DOL of pulmonary hypertension resolution	109	200	69	36
Blood products given	66	59	60	83
Intraventricular hemorrhage	No	No	No	No
Days requiring TPN	173	183	154	89
Day of life to tolerance of full enteral feeds	179	185	190	93
Chronic dialysis mode patient was transitioned to after stabilization	Peritoneal dialysis	Intermittent hemodialysis	Peritoneal dialysis	Peritoneal dialysis
Days in hospital	210	235	201	113
Age at discharge	210 days	235 days	201 days	113 days
Outcome at discharge	Alive	Alive	Alive	Alive
Kidney transplant	Yes	Yes	Listed	Not yet listed due to size
Age at transplant	3 years 1 month 15 days	1 year 11 months 26 days		
Age now	4 years 0 months	3 years 9 months	3 years 0 months	1 year 1 month

**Abstract 039**

**Multicenter epidemiology of cardiac surgery-associated acute kidney injury in neonates undergoing the Norwood operation**

Rebecca Bertrandt^1*^, Denise Hasson^2^, Huaiyu Zang^3^, Garrett Reichle^4^, Catherine Krawczeski^5^, David Winlaw^3^, David Bailly^6^, Stuart Goldstein^3^, David Selewski^7^, Sarah Tabbutt^8^, Katja Gist^3^, and Jeffrey Alten^3^

^*1*^ *Children’s Wisconsin, Medical College of Wisconsin, Milwaukee, WI, USA;* ^*2*^*New York University Langone Health, New York University Grossman School of Medicine, New York, NY, USA;* ^*3*^*Cincinnati Children's Hospital Medical Center, University of Cincinnati College of Medicine, Cincinnati, OH, USA;* ^*4*^*University of Michigan Medical School, Ann Arbor, Michigan, USA;* ^*5*^*Nationwide Children’s Hospital, Columbus, OH, USA;* ^*6*^*Primary Children’s Hospital, University of Utah, Salt Lake City, UT, USA;* ^*7*^*Medical University of South Carolina, Charleston, SC, USA;* ^*8*^*Benioff Children’s Hospital, University of California San Francisco, San Francisco, CA, USA*

**Background:** Cardiac surgery-associated acute kidney injury (CS-AKI) is associated with adverse outcomes in congenital heart disease patients. Single center studies suggest CS-AKI rate is high after the Norwood procedure (NP)^1-5^, the cardiac surgical cohort with presumed highest risk for postoperative complications. Here we present the first data describing the multicenter epidemiology of neonatal CS-AKI after NP, including outcomes associations.

**Methods:** Retrospective cohort study of neonates ≤ 30 days in the Neonatal and Pediatric Heart and Renal Outcomes Network who underwent NP. Neonatal modification of The Kidney Disease Improving Global Outcomes (KDIGO) criteria was used. Perioperative predictors of CS-AKI and its association with morbidity and mortality were examined using serum creatinine SCr, urine output (UOP), and combined SCr/UOP criteria. Sensitivity analysis excluding patients who received prophylactic peritoneal dialysis (PD) was performed.

**Results:** Of 347 patients who underwent NP, CS-AKI occurred in 231 (67%); 141 (41%) incurred maximum Stage 1, 51 (15%) maximum Stage 2, and 39 (11%) maximum Stage 3. Stage 1 CS-AKI peaked on POD0; severe CS-AKI (stages 2 and 3) peaked on POD1. In univariate analysis, prematurity, source of pulmonary blood flow, cardiopulmonary bypass or cross clamp time, use of modified ultrafiltration, and delayed sternal closure were not associated with severe CS-AKI. In multivariable analysis, preoperative feeding was protective (OR=0.48, 0.27-0.86) while prophylactic PD (OR=3.67, 1.88-7.19) was associated with severe CS-AKI (Table 1); though prophylactic PD was not associated with increased SCr. Major postoperative complication or infection (OR=5.05, 1.48-17.18) and high inotrope need (OR=3.54, 1.14-11.03) were associated with mortality, while severe CS-AKI was not. Severe CS-AKI was also not associated with days free of respiratory support or hospital-free days. Multivariate results were similar when patients who received prophylactic PD were excluded.

**Conclusions:** CS-AKI, as defined by KDIGO, occurs frequently in neonates after the NP, but does not independently drive postoperative outcomes in this surgical cohort. Further work is needed to create definitions that identify clinically important phenotypes of neonatal CS-AKI after the NP, particularly in the context of confounding factors such as center-specific practice of prophylactic PD, which may simply represent low UOP rather than true kidney injury.

**Keywords:** acute kidney injury; Norwood procedure; hypoplastic left heart syndrome; congenital heart surgery

**Disclosures:** Authors have no disclosures.

**References:**

1. Blinder JJ, Goldstein SL, Lee VV, et al: Congenital heart surgery in infants: Effects of acute kidney injury on outcomes. J Thorac Cardiovasc Surg 2012; 143:368–374

2. Garcia RU, Natarajan G, Walters HL, et al: Acute kidney injury following first-stage palliation in hypoplastic left heart syn-drome: Hybrid versus Norwood palliation. Cardiol Young 2018; 28:261–268

3. Wong, JH, Selewski, DT, Yu, S, et al. Severe acute kidney injury following stage 1 norwood palliation: effect on outcomes and risk of severe acute kidney injury at subsequent surgical stages. Pediatr Crit Care Med 2016; 17: 615–623

4. Goldstein, BH, Goldstein, SL, Devarajan, P, et al. First-stage palliation strategy for univentricular heart disease may impact risk for acute kidney injury. Cardiol Young 2018; 28: 93–100

5. Alten JA, Cooper DS, Blinder JJ, et al. Epidemiology of Acute Kidney Injury After Neonatal Cardiac Surgery: A Report from the Multicenter Neonatal and Pediatric Heart and Renal Outcomes Network. Crit Care Med. 2021 Oct 1;49(10):e941-e951

**Table 1.** Logistic Regression: Mortality

**Table:** 

	**Outcome: Mortality**
**Variable**	**OR (95% CI)**
Severe CS-AKI (Stage 2-3 vs 0-1)	2.57 (0.81-8.11)
Chromosomal anomaly	1.27 (0.23-7.13)
Preop inotropes or mechanical ventilation	0.86 (0.28-2.66)
Vasoactive inotropic score >15 on postop day 0	**3.54 (1.14-11.03)**
Time to 1st day of negative fluid balance	0.69 (0.19-2.52)
Blalock-Thomas-Taussig shunt	0.67 (0.17-2.61)
Major postop complication or infection	**5.05 (1.48-17.18)**

Odds ratio (OR) and 95% confidence interval (CI) obtained by multivariable logistic regression model for mortality.

**Abstract 040**

**Recurrent neonatal acute kidney injury: Incidence, predictors, and outcomes in the neonatal intensive care unit**

Katherine Vincent^1*^, DNP, NNP, Austin Rutledge^1^, DO, Zegilor Laney^1^, MD, Jill C. Newman^1^, MS, David T. Selewski^1^, MD, MSCR, Heidi J. Steflik^1^, MD, MSCR

^*1*^ *Medical University of South Carolina, Charleston, SC, USA*

**Background:** A single published manuscript specifically examines recurrent AKI (rAKI) in the neonatal intensive care unit (NICU) but was limited to those born <28 weeks of gestational age (WGA) [1]. We aimed to determine the incidence and identify predictors of rAKI and investigate associations between rAKI and clinical outcomes in neonates of all gestational ages in a NICU with protocolized care of neonatal AKI.

**Methods:** We conducted a single-center, retrospective cohort study including neonates cared for in the NICU between 01/01/2020-06/30/2021. The modified neonatal KDIGO criteria were used to identify and stage AKI. Each AKI episode ended with serum creatinine returning to baseline; rAKI episodes were diagnosed based on KDIGO criteria. Outcome measures included survival, length of mechanical ventilation, length of NICU stay, length of hospitalization (LOH), mortality, and the presence of hypertension (HTN) at discharge. Comparisons were made between no AKI, single AKI (sAKI), and rAKI groups using Chi Squared and Kruskal-Wallis tests. Multivariable linear and logistic regression models examining associations between rAKI and outcomes are in development.

**Results:** Of the 869 neonates included, 705 (81.1%) had no AKI, 100 (11.5%) had sAKI, and 64 (7.4%) had rAKI. Compared to infants without AKI, infants with rAKI had lower birthweight (no AKI 2167±910 grams, sAKI 1755±957 grams, rAKI 1639±1098 grams; p<0.001) and gestational age (no AKI 33±4 WGA, sAKI 31±5 WGA, rAKI 30±6 WGA; p<0.001). The distribution of AKI by KDIGO stage did not differ between neonates with sAKI and rAKI. All outcomes, except HTN, differed between groups, and infants with rAKI experienced longer durations of mechanical ventilation, NICU stay, and LOH. Survival also differed by group (Table).

**Conclusion:** Preliminary findings suggest infants with rAKI in our large, single center cohort cared for in a NICU where AKI detection and care is standardized did not experience more severe AKI but did have longer durations of mechanical ventilation, NICU stay, LOH, and decreased survival than those with no or sAKI episodes. Further models comparing outcomes in those with sAKI and rAKI are in process. These findings suggest rAKI is a clinically important occurrence that warrants special attention and further study.

**Keywords:** acute kidney injury, neonatal intensive care unit, recurrent

**Disclosures:** HJS employer received a grant from Baxter. The other authors declare no conflicts of interest.

**Corresponding author:** Katherine Vincent, DNP

**References:**

1. Adegboyega OO, et al. *Pediatr Res*. 2021;online ahead of print. http://doi.org/10.1038/s41390-021-01740-y.

**Table.** Associations between no Acute Kidney Injury (AKI), single AKI, and recurrent AKI and Clinical Outcomes

**Table:** 

**Clinical Outcomes**	**No AKI** (n=705)	**sAKI** (n=100)	**rAKI** (n=64)	**p-value**
Length of mechanical ventilation (days)	0 [0-1]	2 [0-15]	21 [8.5-47]	**<0.001**
Length of NICU stay (days)	21 [11-37]	44 [17-81.5]	80 [52.5-115]	**<0.001**
Length of hospitalization (days)	21 [11-37]	44 [17-81.5]	98 [54.5-127]	**<0.001**
Survival to hospital discharge	697 (98.9)	86 (86.0)	57 (89.1)	**<0.001**
Hypertension at hospital discharge	14 (2.0)	3 (3.0)	1 (1.6)	0.761

Categorical variables presented as count (percentage). Continuous data presented as median [interquartile range].

**Abstract 041**

**Clinical characteristics of patients receiving continuous kidney replacement therapy: Preliminary report from the Worldwide Exploration of Renal replacement Outcomes Collaborative in Kidney Disease (WE-ROCK)**

Shina Menon^1*^ MD, Ayse A Arikan^2^ MD, Dana Y Fuhrman^3^ DO, MSc, Kelli Krallman^4^ MD, Theresa Mottes^5^ NP, Zaccaria Ricci^6^ MD, David Selewski^7^ MD, MSc, Natalja Stanski^4^ MD, Danielle E Soranno^8^ MD, Michelle C Starr^8^ MD, MPH, Michael Zappitelli^9^ MD, MSc, Katja M Gist^4^, DO, MSc, On behalf of WE-ROCK

^*1*^ *University of Washington and Seattle Children’s Hospital, Seattle, WA;* ^*2*^*Texas Children’s Hospital and Baylor College of Medicine, Houston, TX;* ^*3*^*Children’s Hospital of Pittsburgh, University of Pittsburgh School of Medicine, Pittsburgh PA;* ^*4*^ *Cincinnati Children’s Hospital Medical Center, Cincinnati OH;* ^*5*^*Lurie Children’s Hospital, Chicago, IL;* ^*6*^*Bambino Gesu Hospital, Rome, Italy;* ^*7*^*Medical University of South Carolina, Charleston, SC;* ^*8*^*Indiana University School of Medicine, Indianapolis, IN, USA;* ^*9*^*Hospital for Sick Children, Toronto, Canada*

**Background:** Continuous kidney replacement therapy (CKRT) is the most frequently used modality of dialysis in critically ill patients with hemodynamic instability. There remains significant variability in how CKRT is prescribed and delivered. The Worldwide Exploration of Renal replacement Outcomes Collaborative in Kidney Disease (WE-ROCK) was established in 2021 to evaluate characteristics and clinical and patient centered outcomes among children receiving CKRT.

**Methods:** WE-ROCK data includes 32 centers spanning 6 countries from 2013-2021. Data collection: demographics, pre-CKRT initiation data, daily CKRT data (7 days), data for the first 7 days following CKRT liberation, and discharge data (vital status, CKRT dependence, functional outcomes). Exclusion criteria: end stage kidney disease, received dialysis during the same ICU admission prior to CKRT initiation, received CKRT for a non-renal indication, or concurrent ECMO.

**Results:** 994 patients were included. Ages were newborn to 25 years and weights from 1.9 to 179 kg. The most common reason for ICU admission was shock/infection/major trauma (n=377, 37.7%) followed by respiratory failure (196, 19.6%), and the median PRISMIII score was 14 (IQR: 9, 18). Patients spent a median of 2 d in the ICU before CKRT (range 0 to 285). Sepsis at CKRT initiation was present in 433 (43.5%). Most patients were initiated with a non-tunneled (n=684, 68.8%) catheter in the internal jugular vein (n=583, 58.6%). Continuous Veno-venous Hemodiafiltration (76%) was the most common modality. The initial prescribed dose of CKRT was 2068 (IQR 1686, 2694) mL/1.73m^2^/hour, and citrate anticoagulation was used in 61.5%. In the first 28 days**,** liberation was not attempted in 318 (34%), 305 (33%) failed to liberate and 312 (34%) successfully liberated from CKRT. Overall, 623 (62.6%) and 600 (60.3%) survived to ICU and hospital discharge respectively. Major adverse kidney events (composite of death, need of RRT, or worsened kidney function) at 90 days were seen in 606 (60.9%).

**Conclusion:** We describe preliminary results of demographics and clinical characteristics of critically ill patients receiving CKRT. Major adverse kidney events at 90 days are seen in the majority of those who receive CKRT. More detailed analyses are needed to determine the factors associated with worse outcomes.

**Keywords:** continuous renal replacement therapy, dialysis, critical care nephrology

**Disclosures:** The authors declared no competing interests.

**Corresponding Author:** Shina Menon

**References:** None

**Table 1. Patient and clinical characteristics of patients in the Worldwide Exploration of Renal replacement Outcomes Collaborative in Kidney Disease (WE-ROCK) cohort**

**Table:** 

Characteristic	N=994
**Female**	448 (45.1)
**Reason for Admission to ICU**	
Shock / Infection / Major Trauma	377 (37.7)
Respiratory Failure	196 (19.6)
AKI and/or fluid overload	66 (6.6)
Primary Cardiac: Congenital Heart Disease	32 (3.2)
Primary Cardiac: Heart Failure and/or Cardiomyopathy	34 (3.4)
Primary Cardiac: Post-Surgical Congenital Heart disease	48 (4.8)
Other	142 (14.2)
Post-surgical / Minor Trauma	53 (5.3)
Central Nervous System Dysfunction	38 (3.8)
Pain / Sedation Management	8 (0.8)
**Sepsis at admission**	459 (46.1)
**Sepsis at CRRT**	433 (43.5)
**Age, years**	8.7 (1.5, 14.9)
**PRISM3**	14 (9, 18)
**Weight, kg**	26 (10.70, 54.42)
**Vasoactive Inotropic Score within 24 hours of CKRT**	4.6 (0, 19.7)
**CKRT modality**	
CVVH	113 (11.3)
CVVHD	101 (10.2)
CVVHDF	755 (76)
CVVH with Aquadex	9 (0.9)
SCUF	12 (1.2)
**Blood flow (mL/min)**	100 (60, 150)
**CKRT Dose, mL/1.73m** ^**2**^ **/hour**	2068 (1686, 2694)
**CKRT Dose, mL/kg/hour**	41.67 (30.5, 58.9)
**Anticoagulation**	
Regional Citrate	612 (61.2)
Heparin	252 (252)
Prostacyclin	41 (4.1)
None	72 (7.2)
Other	13 (1.3)
**Initial Catheter (N=830)**	
Non-tunneled	684 (68.8)
Tunneled	146 (14.6)
**Site of Catheter placement (N=854)**	
Femoral	236 (23.6)
Internal Jugular	583 (58.6)
Other	19 (1.9)
Subclavian	16 (1.6)
**Liberation from CKRT (N=954)**	
Failed liberation	305 (30.5)
Successful liberation	312 (31.4)
Never attempted	318 (31.9)
**ICU Length of Stay**	22 (10, 43
**Ventilation Days**	9 (3, 21)
**Survival to ICU discharge**	623 (62.6)
**Hospital Length of Stay**	42 (22, 81)
**Survival to Hospital Discharge**	600 (60.3)
**RRT dependence at hospital discharge**	103 (10.3)
**MAKE90**	60.9 (60.9)

**Abstract 042**

**Characteristics of liberation patterns in critical ill children receiving continuous kidney replacement therapy: An analysis of the Worldwide Exploration of Renal replacement Outcomes Collaborative in Kidney Disease (WE-ROCK)**

Shina Menon^1*^ MD, Ayse A Arikan^2^ MD, Dana Y Fuhrman^3^ DO, MSc, Kelli Krallman^4^ MD, Theresa Mottes^5^ NP, Zaccaria Ricci^6^ MD, David Selewski^7^ MD, MSc, Natalja Stanski^4^ MD, Danielle E Soranno^8^ MD, Michelle C Starr^8^ MD, MPH, Michael Zappitelli^9^ MD, MSc, Katja M Gist^4^, DO, MSc, On behalf of WE-ROCK

^*1*^ *University of Washington and Seattle Children’s Hospital, Seattle, WA;* ^*2*^*Texas Children’s Hospital and Baylor College of Medicine ,Houston, TX;* ^*3*^*Children’s Hospital of Pittsburgh, University of Pittsburgh School of Medicine, Pittsburgh PA;* ^*4*^ *Cincinnati Children’s Hospital Medical Center, Cincinnati OH;* ^*5*^*Lurie Children’s Hospital, Chicago, IL;* ^*6*^*Bambino Gesu Hospital, Rome, Italy;* ^*7*^*Medical University of South Carolina, Charleston, SC;* ^*8*^*Indiana University School of Medicine, Indianapolis, IN, USA;* ^*9*^*Hospital for Sick Children, Toronto, Canada*

**Background:** The burden of acute kidney injury is high, affecting more than 25% of critically ill children. Approximately 3-7% require continuous kidney replacement therapy (CKRT). The Worldwide Exploration of Renal replacement Outcomes Collaborative in Kidney Disease (WE-ROCK) was established in 2021 to evaluate characteristics and clinical and patient-centered outcomes among critically ill children receiving CKRT. The purpose of this study was to describe the liberation patterns and outcomes in children receiving CKRT.

**Methods:** WE-ROCK encompasses data from 32 centers spanning 6 countries from 2013-2021. Data collection included baseline demographics, pre-CKRT initiation data, daily CKRT data (first 7 days), including prescription, data for 7 days following CKRT liberation, and discharge data (vital status, CKRT dependence, functional outcomes). Successful liberation was defined as >48 hours off CKRT. Exclusion criteria: end stage kidney disease, received peritoneal dialysis during the same ICU admission prior to CKRT initiation, received CKRT for a non-renal indication (i.e. toxin removal, liver failure), or concurrent ECMO.

**Results:** 935 patients were included (median age 8.7 years [IQR:1.56, 14.98]). Continuous VenoVenous Hemodiafiltration (76%) was the most common modality. In the first 28 days, liberation from CKRT was not attempted in 318 (34%), 305 (33%) failed to liberate and 312 (34%) patients liberated from CKRT (Figure 1). There was no difference in age and PRISMIII score among the 3 groups. Vasoactive inotrope score within 24 hours of CKRT initiation was higher among those in which liberation was not attempted (p=0.002). Among those, in whom liberation was not attempted, only 23% (n=75) and 22% (n=70) survived to ICU and hospital discharge respectively. Major adverse kidney events (the composite of death, need of RRT, or worsened kidney function) at 90 days were significantly higher among those in which liberation was not attempted (93%), compared to those who failed to liberate (63%) and successful liberated (36%)

**Conclusion:** We describe preliminary results of liberation patterns among critically ill children receiving CKRT. Outcomes are significantly worse among those who in which liberation was not attempted or failed to liberate. More detailed analyses are needed to determine the factors associated with failed liberations and outcomes.

**Keywords:** continuous renal replacement therapy, dialysis, critical care nephrology

**Disclosures:** The authors declare no competing interests.

**Corresponding Author:** Shina Menon

**References:** None

**Figure 1. Liberation patterns among children receiving Continuous kidney replacement therapy** 
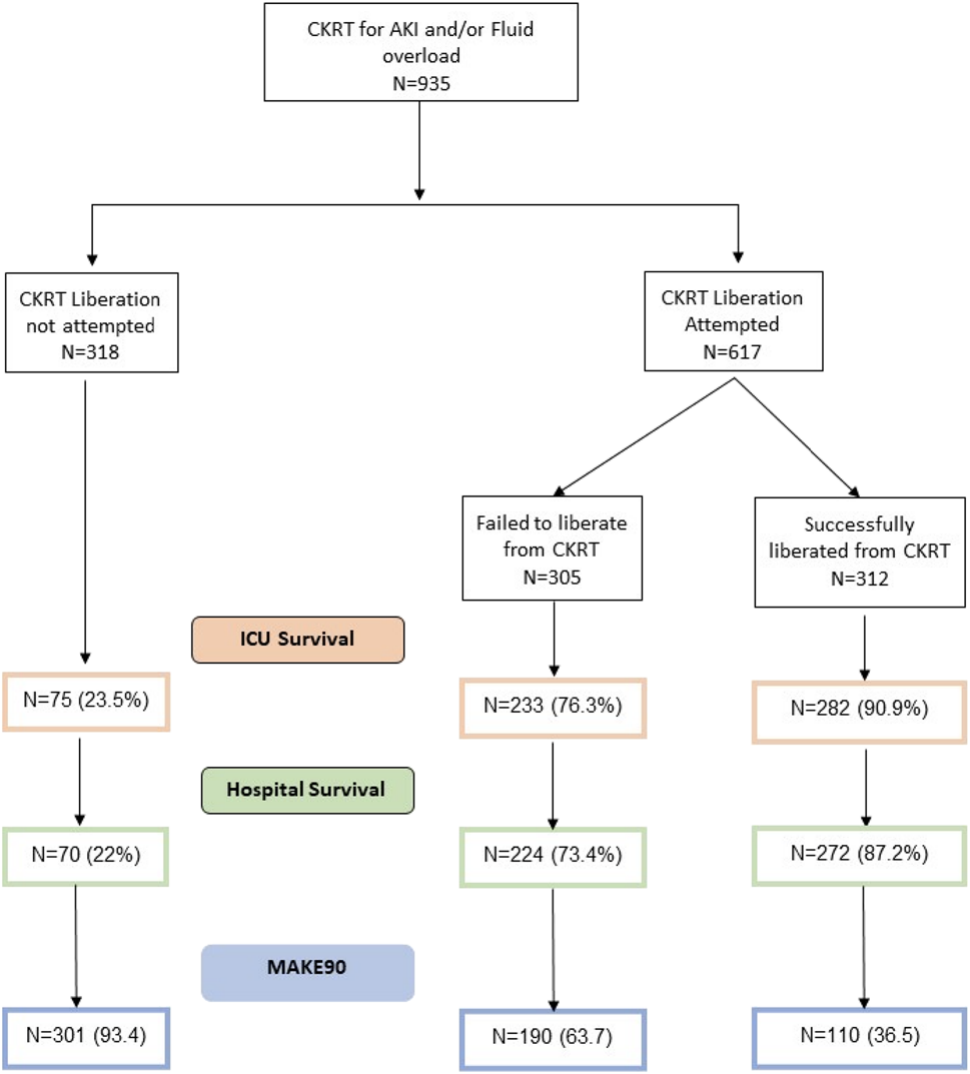


**Abstract 043**

**Zorro-Flow™: an external urine collection device designed for small neonates and infants**

David Askenazi MD, Elizabeth Dechant RN, Jessica Potts RN, Martin Holland

**Introduction: **The ability to collect urine in ICU patients is standard of care because urine alerts the physician about end-organ perfusion. failure to account for oliguria will miss 20-30% of acute kindey injury (AKI), appropriate fluid provision requires knowledge of urine output, and urine analysis is important to diagnose urinary tract infection, electrolyte abnormalities, and nephrolithiasis. In addition, novel urine tests to diagnose AKI are being incorporated into clinical care algorithms. Unfortunately, safe and effective urine collection devices for neonates and small children are not available. Urine catheters are difficult to use and traumatic. Diaper weights are misleading (if they quantify stool). Cotton balls absorb electrolytes and proteins and can’t be used to test for a urinary tract infection. In small children, urine collection bags are poor urine collectors, and the tape can damage the fragile skin of premature neonates.

**Methods: **Using an iterative process, we developed an external urine collection device designed with the female neonates in mind. Each cycle included designing, printing, testing and evaluation. For each cycle, 3-5 female participated after informed consent from the family. During one cycle, we used wall suction of 20 mmHg. Silicone adhesive tape/gel were used to adhere the device to the skin. Testing of skin color, temperature, turgor, moisture and integrity were performed before, immediately after removal and at 24 hours after removal.

**Results: **We tested 82 female neonates and infants with 8 different 3-D printed devices. The average weight was 2.42 kg. The corrected gestational age ranged from 24 weeks to 72 weeks. The 8^th^ design (MK48) is ergonomic and has a ramp to limit urine leak. We did not find any major issues with skin integrity based on formal skin tests, except during the cycle where we used wall suction (4/5 had mild transient skin changes). The design is pictured in the figure below.

**Conclusions: **Zorro-Flow™ is an external urine collection device design for the smallest neonates. Current plans are underway to manufacture a sterile device which will include tubing, a 3-way stopcock, and a urine reservoir bag.

**Disclosures and Funding: **This project was supported by the Tolwani Innovation Grant in Nephrology from UAB, and the i6 Grant.

DA, ED, and MH are listed as inventors in the filed international patents. DA is CSO and founder of Zorro-Flow Inc. DA serves as consultant and /or receives grant funding from Baxter, Medtronic, Nuwellis, Seastar, Bioporto and Portero.

**Figure: Zorro-Flow™ Device**